# Mitochondria-mediated ferroptosis induced by CARD9 ablation prevents MDSCs-dependent antifungal immunity

**DOI:** 10.1186/s12964-024-01581-2

**Published:** 2024-04-02

**Authors:** Zhiyong Zhang, Pengfei Li, Ying Chen, Yuxi Chen, Xiuzhu Wang, Sunan Shen, Yue Zhao, Yanan Zhu, Tingting Wang

**Affiliations:** 1 Department of Endodontic, Nanjing Stomatological Hospital, Affiliated Hospital of Medical School, Research Institute of Stomatology, Nanjing University, Nanjing, 210008 China; 2 The State Key Laboratory of Pharmaceutical Biotechnology, Chemistry and Biomedicine Innovation Center (ChemBIC), Division of Immunology, Medical School, Nanjing University, Nanjing, 210093 China; 3 Jiangsu Key Laboratory of Molecular Medicine, Division of Immunology, Medical School, Nanjing University, Nanjing, 210093 China

**Keywords:** CARD9, Disseminated candidiasis, MDSCs, SLC7A11, Ferroptosis, Mitochondrial OXPHOS

## Abstract

**Background:**

Caspase Recruitment Domain-containing protein 9 (CARD9) expressed in myeloid cells has been demonstrated to play an antifungal immunity role in protecting against disseminated candidiasis. Hereditary CARD9 ablation leads to fatal disseminated candidiasis. However, the myeloid cell types and molecular mechanisms implicated in CARD9 protecting against disseminated candidiasis remain wholly elusive.

**Methods:**

The role of CARD9 ablation in exacerbating disseminated candidiasis was determined in vivo and in vitro. The molecular mechanism by which CARD9 ablation promotes acute kidney injury in disseminated candidiasis was identified by RNA-sequencing analysis. The expression of mitochondrial proteins and ferroptosis-associated proteins were measured by Quantitative real-time PCR and western blot.

**Results:**

CARD9 ablation resulted in a reduced proportion of myeloid-derived suppressor cells (MDSCs) and a substantially lower expression of solute carrier family 7 member 11 (SLC7A11) in the kidneys, which increased susceptibility to acute kidney injury and renal ferroptosis during disseminated *Candida tropicalis* (*C. tropicalis*) infection. Moreover, CARD9-deficient MDSCs were susceptible to ferroptosis upon stimulation with *C. tropicalis*, which was attributed to augmented mitochondrial oxidative phosphorylation (OXPHOS) caused by reduced SLC7A11 expression. Mechanistically, C-type lectin receptors (CLRs)-mediated recognition of *C. tropicalis* promoted the expression of SLC7A11 which was transcriptionally manipulated by the Syk-PKCδ-CARD9-FosB signaling axis in MDSCs. FosB enhanced SLC7A11 transcription by binding to the promoter of SLC7A11 in MDSCs stimulated with *C. tropicalis*. Mitochondrial OXPHOS, which was negatively regulated by SLC7A11, was responsible for inducing ferroptosis of MDSCs upon *C. tropicalis* stimulation. Finally, pharmacological inhibition of mitochondrial OXPHOS or ferroptosis significantly increased the number of MDSCs in the kidneys to augment host antifungal immunity, thereby attenuating ferroptosis and acute kidney injury exacerbated by CARD9 ablation during disseminated candidiasis.

**Conclusions:**

Collectively, our findings show that CARD9 ablation enhances mitochondria-mediated ferroptosis in MDSCs, which negatively regulates antifungal immunity. We also identify mitochondria-mediated ferroptosis in MDSCs as a new molecular mechanism of CARD9 ablation-exacerbated acute kidney injury during disseminated candidiasis, thus targeting mitochondria-mediated ferroptosis is a novel therapeutic strategy for acute kidney injury in disseminated candidiasis.

**Supplementary Information:**

The online version contains supplementary material available at 10.1186/s12964-024-01581-2.

## Background

The incidence and mortality of human disseminated candidiasis are escalating all over the world [1–3]. As an opportunistic pathogenic fungal pathogen in humans, *Candida tropicalis* (*C. tropicalis*) has been identified as a significant risk factor to disseminated candidiasis in immunocompromised patients with neutropenia, Acquired Immune Deficiency Syndrome, and cancer [4]. Although many antifungal drugs have been accessible, their clinical effectiveness is very poor, resulting in drug resistance, unexpected side effects, and a high morbidity and mortality of patients with fungal infections [2, 5, 6]. As a result, interest in immunotherapy is growing and there is a compelling need to increase our knowledge of the underlying molecular mechanisms of host antifungal immunity, which is indispensable for the creation of alternative innovative therapies.

Primarily the innate and adaptive immune systems are responsible for host defense against *Candida* infections. C-type lectin receptors (CLRs), expressed on the surface of myeloid cells including neutrophils, dendritic cells (DC), macrophages and myeloid-derived suppressor cells (MDSCs) exert antifungal effects [7–10]. CLRs mainly consist of Dectin-1, Dectin-2, Dectin-3, and Mincle, which can recognize β-glucan, α-mannan and other components on the fungal cell wall [10–12]. Once stimulated by the fungal cell wall, CLRs signals trigger the activation of Syk-PKCδ-CARD9 signaling pathway, which induces intracellular signaling cascade to stimulate antifungal immune responses [10, 13, 14]. CARD9 is an adaptor protein mainly expressed in myeloid cells and is involved in CLRs-induced antifungal immunity [15, 16]. Evidence suggests that CARD9 knockout leads to impaired antifungal immunity and aggravated *Candida* infection [15, 17, 18]. In humans, CARD9 loss-of-function mutations are closely linked to susceptibility to fungal infections [19–22]. However, the cellular and molecular basis of CARD9 deficiency-aggravated disseminated *C. tropicalis* infection is not well understood.

MDSCs are defined as a heterogeneous group of innate immune cells that includes immature myeloid-derived cells and myeloid progenitor cells [23]. MDSCs have strong immunosuppressive properties and play a vital biological role in infectious diseases, inflammation and tumors [24, 25]. Opportunistic pathogenic fungi have been reported to induce the production of MDSCs, which are resistant to systemic fungal infections [26]. We have previously observed that *C. tropicalis* increases MDSCs accumulation and immunosuppressive activity [8, 27]. Nevertheless, it remains unclear that whether invasive *C. tropicalis* infection aggravated by CARD9 deficiency is associated with decreased MDSCs accumulation.

Ferroptosis is defined as a regulated kind of non-apoptotic cell death characterized by iron-dependent phospholipid peroxidation [28]. This atypical type of cell death is induced by loss of the antioxidant system SLC7A11/xCT-glutathione (GSH)-glutathione peroxidase 4 (GPX4), excessive accumulation of free iron ions and lipid reactive oxygen species (ROS), as well as the metabolites of lipid peroxidation including 4-hydroxynonenal (4HNE), malondialdehyde (MDA) [29]. SLC7A11, the cystine/glutamate antiporter, also referred to as xCT, is responsible for the extracellular export of glutamate and intracellular import of cystine for the biosynthesis of intracellular GSH. GPX4 subsequently utilizes GSH to perform an antioxidant function and prevent ferroptosis [30, 31]. Previous studies have revealed that ferroptosis is implicated in the pathological processes of a wide range of illnesses, including acute kidney injury, ischemia-reperfusion injury, carcinogenesis, neurodegenerative diseases, fungal infections [30, 32–35]. Recent research has reported that ferroptosis worsens renal immunopathology and injury during disseminated candidiasis [36]. In addition, emerging data demonstrate that MDSCs are susceptible to ferroptosis in the tumour microenvironment [37]. However, it is incompletely clear whether CARD9 deficiency causes ferroptotic cell death of MDSCs in the kidneys, resulting in reduced accumulation of MDSCs in the kidneys during invasive *C. tropicalis* infection.

In this work, we intend to explore the myeloid cell types and molecular mechanisms involved in CARD9-dependent antifungal immunity during disseminated *C. tropicalis* infection. We demonstrate that CARD9 deficiency-aggravated acute kidney injury and renal ferroptosis in disseminated *C. tropicalis* infection results from decreased MDSCs accumulation and SLC7A11 expression in the kidneys. Furthermore, CARD9 deficiency promotes mitochondria-mediated ferroptosis in MDSCs upon *C. tropicalis* stimulation, which is attributed to reduced expression of SLC7A11. Finally, inhibition of mitochondrial OXPHOS or ferroptosis alleviates CARD9 deficiency-aggravated acute kidney injury during disseminated *C. tropicalis* infection. These data provide new insight into how CARD9 deficiency-induced mitochondria-mediated ferroptosis negatively modulates antifungal immunity.

## Methods

### Reagents

Ferrostatin-1 (Fer-1) (SML0583-25MG), Laminarin (LAM) obtained from Laminaria digitata (L9634-100MG), GW5074 (G6416-5MG), Dimethyl 2-oxoglutarate (α-KG) (349,631), metformin hydrochloride (PHR1084-500MG) were purchased from Sigma. R406 (HY-12,067), Rottlerin (HY-18,980), Sulfasalazine (HY-14,655), Erastin (HY-15,763), FCCP (HY-100,410) were purchased from MedChemExpress. Oligomycin (ab141829) was obtained from Abcam.

### Mice

*Card9*^−/−^ and *Clec4d*^*−/−*^ mice on the C57BL/6J genetic background were generously given by Dr. Xin Lin (Tsinghua University, Beijing, China). 6- to 8-week-old female wild-type (WT) mice on the C57BL/6J genetic background were obtained from Jiangsu Huachuang Xinnuo Pharmaceutical Technology Co., Ltd. (Taizhou, China). These animals were bred in a specific pathogen-free environment at Medical School of Nanjing University. All mouse studies were conducted according to the NIH “Guide for the Care and Use of the Laboratory Animal”. Additionally, all animal experiments were reviewed and approved by the Institutional Animal Care and Use Committee (IACUC-D2202134) of Nanjing University.

### Culture of *C. tropicalis *strain

The *C. tropicalis* strain (W4162870) was provided by Dr. Xin Lin (Tsinghua University, Beijing, China). A single colony of *C. tropicalis* was inoculated into Liquid Sabourand Medium with 4% glucose and 1% mycological peptone, and cultured at 30 ℃ for 18–24 h. Then *C. tropicalis* yeast cells were washed in PBS.

### Murine disseminated candidiasis model

6- to 8-week-old female mice were intravenously injected with 2×10^5^ CFU of *C. tropicalis* in 100 µL sterile PBS. The mice were monitored daily for weight and survival following injection. On the fifth day, the mice were euthanized and the serum, kidney, spleen and bone marrow (BM) were removed and collected. And serum collection did not affect flow cytometry panel. For activation and inhibition of mitochondrial OXPHOS experiments, infected WT mice were intraperitoneally injected with PBS (Vehicle) or α-KG (500 mg/kg) per day. Infected *Card9*^−/−^ mice were treated with PBS (Vehicle) or metformin (200 mg/kg) by oral gavage daily. For ferroptosis inhibition experiments, infected *Card9*^−/−^ mice were intraperitoneally injected with a single dose of 20 µL ferrostatin-1 (Fer-1, 10 mg/kg) every day. As a control, 20 µL DMSO (purity: ≥99.9%) was employed. Generally, the proportion of DMSO in mice below 5% is tolerable. Therefore, the dosage of DMSO in our study was not toxic to mice.

### In vitro culture of MDSCs

Generation of bone marrow-derived MDSCs were prepared according to the previous description [7, 8]. In brief, bone marrow cells were extracted from the tibias and femurs of mice. Erythrocytes were lysed with ACK Lysis Buffer (Beyotime Biotechnology, C3702-500 ml). Then, the cells were grown in complete RPMI-1640 medium containing 40 ng/ml murine IL-6 (Miltenyi Biotec, 130-096-682) and 40 ng/ml murine GM-CSF (Miltenyi Biotec, 130-095-742) for 4 days. During the 4 days of cell culture, the cells were always placed in an incubator at 37 ℃ and 5% CO_2_. After 4 days of culture, the cells were removed from the incubator, and the suspended cells were carefully collected, discarding the adherent cells. Follow-up experiments were performed using the collected suspended cells.

### Histopathology analysis

The kidney tissues were fixed in 4% paraformaldehyde solution, and embedded in paraffin. Then, the sections of paraffin-embedded kidney tissues were stained with periodic-acid-Schiff (PAS) and hematoxylin and eosin (H&E). A Digital Slide Scanner (PANNORAMIC SCAN II, 3D HISTECH) was used to scan the stained slides. The inflammatory score and fungal burden of the kidneys were assessed as previously reported [38–40].

For immunohistochemistry (IHC) assay, the renal tissues sections were stained with the indicated primary antibodies. The stained sections were scanned by a Digital Slide Scanner. The ImageJ software was used to quantify the proportion of positive cells. The primary antibodies used were as follows: SLC7A11 (xCT) (Abcam, ab307601, 1:500), GPX4 (Abcam, ab125066, 1:100), Ly-6G (Abcam, ab238132, 1:2000), F4/80 (Cell Signaling Technology, 70076T, 1:250) and CD11c (Abcam, ab219799, 1:100).

### Immunofluorescence

The slides of paraffin-embedded kidney tissue were incubated with anti-4-Hydroxynonenal (4HNE) (Novus Biologicals, NBP2-59353-25ug) primary antibody at a 1:50 dilution overnight. Then the slides were incubated with Goat Anti-Mouse IgG H&L (Alexa Fluor® 647) (Abcam, ab150115) secondary antibody at a 1:500 dilution for 1 h at room temperature in the dark. Cell nuclei were labeled with DAPI (Working concentration: 5 µg/ml) as a counterstain. A Digital Slide Scanner was implemented to observe the stained sections.

For the determination of mitochondrial mass, MDSCs were pre-incubated with 200 nM MitoTracker Green (ThermoFisher Scientific, M7514). The cells were then adhered to poly-D-lysine-coated slides. Next, 4% paraformaldehyde was used to fix the cells, and 0.1% Triton X-100 was used to permeabilize them. Cell nuclei were labeled with DAPI (Working concentration: 5 µg/ml) as a counterstain. Finally, a laser scanning confocal microscope was used to examine the stained slides and obtain immunofluorescence images.

### Terminal deoxynucleotidyl transferase dUTP nick-end labeling (TUNEL) assay

The cell death in infected kidneys was determined by TUNEL Detection Kit (Beyotime Biotechnology, C1088) according to the instructions of manufacturer. In brief, kidney tissue sections were incubated with proteinase K (Working concentration: 20 µg/ml) for 30 min at 37 ℃. Subsequently, the slides were incubated with the TUNEL reaction mixture for 60 min at 37 ℃. The stained slides were scanned by a Digital Slide Scanner.

### Quantitative real‑time PCR

Total RNA was isolated from MDSCs or kidneys using TRIzol Reagent. Then cDNA was reverse-transcribed from the extracted RNA using HiScript III RT SuperMix for qPCR Kit following the protocol of manufacturer. Quantitative real-time PCR (qPCR) was performed using SYBR green PCR master mix on an ABI Vii 7 sequence detection system or a Step One Plus sequence detection system (Applied Biosystems, Thermo Fisher Scientific, US). The relative expression of genes was normalized to β-actin and was calculated through the the 2^−ΔΔCT^ method. The primers for qPCR are shown in Table. S1.

### Western blotting analysis

RIPA Lysis Buffer was used to lyse kidney tissue and MDSCs. The protein concentration was determined by BCA Protein Assay Kit (Beyotime Biotechnology, P0010). SDS-PAGE was used to separate the equivalent quantities of protein from the total cell lysate and transfer it to polyvinylidene difluoride (PVDF) membranes. The PVDF membranes were incubated with the prescribed primary antibodies overnight at 4 ℃ and secondary antibodies for 1.5 h at room temperature. Then the protein bands were visualized using an enhanced chemiluminescence kit and quantified by Image J software. The primary antibodies used were shown below: β-actin (Cell Signaling Technology, 8457S, 1:1000), SLC7A11 (xCT) (Cell Signaling Technology, 98051S, 1:1000), Glutathione Peroxidase 4 (GPX4) (Abcam, ab125066, 1:5000), 4-Hydroxynonenal (4HNE) (Novus Biologicals, NBP2-59353-25ug, 1:1000), Phospho-PKCdelta (Tyr311) (Cell Signaling Technology, 2055S, 1:1000), PKCδ (Cell Signaling Technology, 9616T, 1:1000), FosB (Cell Signaling Technology, 2251S, 1:1000), GLUD1 (Proteintech, 14299-1-AP, 1:10000), NDUFB8 (Proteintech, 14794-1-AP, 1:10000), SDHB (Proteintech, 10620-1-AP, 1:10000), UQCRC2 (Proteintech, 14742-1-AP, 1:10000), COX IV (Cell Signaling Technology, 4850T, 1:1000), ATP5A1 (Proteintech, 14676-1-AP, 1:10000), Tom20 (Abcam, ab186735, 1:5000), Tim23 (Proteintech, 11123-1-AP, 1:3000). All fully unprocessed and uncropped membrane blots are presented in Supplementary Material 2.

### Flow cytometry

Kidneys, spleens and bone marrow were collected and single-cell suspensions were prepared. To determine the number of MDSCs, the single-cell suspensions were stained with fluorescent conjugated antibodies: PE/Cy7 anti-mouse CD45 (BioLegend, 103,114, 2.5 µg/ml), FITC anti-mouse/human CD11b (BioLegend, 101,206, 2.5 µg/ml) and APC anti-mouse Ly-6G/Ly-6C (Gr-1) (BioLegend, 108,412, 2.5 µg/ml) at 4 °C for 30 min in the dark. For the detection of neutrophils in the kidneys, the single-cell suspensions were stained with fluorescent conjugated antibodies: PE/Cy7 anti-mouse CD45 (BioLegend, 103,114, 2.5 µg/ml), FITC anti-mouse/human CD11b (BioLegend, 101,206, 2.5 µg/ml) and APC anti-mouse Ly-6G (BioLegend, 164,505, 5 µg/ml). For the detection of macrophages in the kidneys, the single-cell suspensions were stained with fluorescent conjugated antibodies: PE/Cy7 anti-mouse CD45 (BioLegend, 103,114, 2.5 µg/ml), FITC anti-mouse/human CD11b (BioLegend, 101,206, 2.5 µg/ml) and APC-conjugated anti-mouse F4/80 (BioLegend, 157,305, 5 µg/ml). For the detection of DCs in the kidneys, the single-cell suspensions were stained with fluorescent conjugated antibodies: PE/Cy7 anti-mouse CD45 (BioLegend, 103,114, 2.5 µg/ml), FITC-conjugated anti-mouse CD11c (BioLegend, 117,305, 2.5 µg/ml) and APC anti-mouse I-A/I-E (MHC class II) (BioLegend, 107,613, 2.5 µg/ml). For 7AAD viability staining of BM-derived MDSCs, single-cell suspensions in 0.5 mL of Cell Staining Buffer were incubated with 7AAD Viability Staining Solution (BioLegend, 420,404) for 5–10 min in the dark. The cells were washed with PBS and detected using a FACSCalibur flow cytometer (BD Biosciences). The collected data were analyzed with FlowJo software version V10.

### RNA sequencing

The renal tissues of *C. tropicalis*-infected mice were collected. Total RNA was isolated using TRIzol Reagent (Invitrogen). The libraries were constructed using VAHTS Universal V6 RNA-seq Library Prep Kit. The RNA sequencing and analysis were performed by OE Biotech Co., Ltd. (Shanghai, China).

HISAT2 [41] was utilized to map the clean reads to the mouse reference genome (GRCm39). DESeq2 was used for differential expression analysis. The threshold for significantly differential expression genes (DEGs) was established at P value < 0.05 and fold change > 2 or fold change < 0.5. Based on the hypergeometric distribution, GO and KEGG pathway enrichment analysis of DEGs were conducted to screen the significant enriched term using R (v 3.2.0), respectively. GSEA software was used for Gene Set Enrichment Analysis (GSEA). The analysis was employed a predefined gene set, and the genes were ranked in accordance with the degree of differential expression in the two types of samples. The predefined gene set was next checked to see if it was enriched at the top or bottom of the ranking list. First, the enrichment score (ES) was calculated. Starting with the first gene in the list where the gene set was located, a cumulative statistic was calculated along the list. The ES was finally defined as the maximum peak of the cumulative statistic. ES > 0 indicated that gene set was enriched at the top of the ranking list. ES < 0 indicated that gene set was enriched at the bottom of the ranking list. Then the significance of ES was evaluated. False discovery rate (FDR) < 0.05 was considered statistically significant. ES > 0 and FDR < 0.05 indicated that the signaling pathways were significantly up-regulated. ES < 0 and FDR < 0.05 indicated that the signaling pathways were significantly down-regulated.

### Chromatin immunoprecipitation (ChIP) assay

ChIP assay was conducted by using a SimpleChIP® Plus Enzymatic Chromatin IP Kit (CST, 9004) according to the guidelines of the manufacturer. In brief, ChIP was implemented by utilizing the antibodies against FosB (CST, 2251S, 1:50) and Rabbit IgG control (CST, 2729, 1:50), and 30 µL ChIP-Grade Protein G Agarose Beads. qPCR analysis was conducted to determine the ChIP-enriched DNA fragments.

### Detection of mitochondrial function and mass, mitochondrial membrane potential

MDSCs were incubated with 200 nM MitoTracker Green (ThermoFisher Scientific, M7514) or 500 nM MitoTracker Orange (ThermoFisher Scientific, M7510) for 15 min. Then flow cytometry was performed by using a FACSCalibur flow cytometer (BD Biosciences). The data were analyzed with software FlowJo V10.

### Metabolic assays

An Agilent Seahorse XFe96 Extracellular Flux Analyzer (Seahorse Bioscience) was employed to detected the Oxygen consumption rate (OCR) of MDSCs. MDSCs were seeded (2×10^5^ cells/well) onto cell culture microplates precoated with poly-D-lysine. The OCR was measured with Seahorse XF Cell Mito Stress Test Kit by the consecutive treatment with oligomycin (1.5 µM), FCCP (1 µM), rotenone + antimycin A (0.5 µM). And the OCR was detected in real time following each drug injection.

### Determination of glutamate, cystine uptake, GSH, MDA, α-KG

Cystine uptake, the levels of glutamate, GSH, MDA and α-KG were measured using Cystine Uptake Assay Kit (DOJINDO, UP05), Glutamate Assay Kit (Colorimetric) (Cell Biolabs, Inc, MET-5080), GSH-Glo™ Glutathione Assay Kit (Promega, V6911), MDA Assay Kit (DOJINDO, M496), α-Ketoglutarate Assay Kit (Sigma, MAK054) according to the instructions of manufacturer. All of the findings were normalized to number of cells.

### Serum TREM1 and NGAL measurement

The levels of serum TREM1 and NGAL were determined with Mouse sTREM-1 ELISA Kit (ZCI BIO, ZC-38,426) and Mouse NGAL ELISA Kit (ZCI BIO, ZC-39,020) following manufacturer’s protocols.

### Detection of blood urea nitrogen (BUN) and serum creatinine (sCr)

Blood urea nitrogen (BUN) and serum creatinine (sCr) concentrations were detected using BUN Assay Kit (ZCI BIO, ZC-S0480) and sCr Assay Kit (ZCI BIO, ZC-A1191) in accordance with manufacturer’s instructions.

### Cell viability assay

The relative cell viability of MDSCs was detected by CCK-8 kit according to protocols of manufacturer. MDSCs were seeded onto 96-well plates. Blank group was set up: culture medium without cells and CCK-8 solution. Negative control group was set up: culture medium containing cells and CCK-8 solution, without drugs. Positive control group was set up: culture medium containing cells, CCK-8 solution and drugs. Following treatment, 10 µl CCK-8 solution was added into each well, and then MDSCs were cultured for 1–2 h at 37 ℃. At 450 nm, a microplate reader was used to detect the absorbance.

### siRNA transfection

siRNA oligonucleotides specifically targeting mouse *Slc7a11* and negative control siRNAs (NC) were obtained from RiboBio (Guangzhou, China). MDSCs were grown in six-well plates and then transfected with 50 nm siRNA utilizing Lipofectamine™ RNAiMAX Transfection Reagent (13778030, Invitrogen) for 24 h following manufacturer’s protocols. The sequences of siRNA targeting *Slc7a11* is listed in Table. S2.

### Construction of recombinant overexpressed adenovirus vector and adenoviral transfection

The Slc7a11-overexpressing adenoviral GV314 vector (CMV-MCS-3FLAG-SV40-EGFP) and control adenoviral vector were constructed and purchased from GeneChem (Shanghai, China). MDSCs were transfected with Slc7a11-overexpressing adenoviral GV314 vector and control adenoviral vector at an MOI of 100 for 24 h according to the guidelines of manufacturer.

### Statistical analysis

Data with error bars were expressed as mean ± SEM from at least three independent experiments. Two-tailed unpaired Student’s *t* test was employed to compare significant differences between the two groups. And statistical analysis among multiple groups was determined by one-way or two-way analysis of variance (ANOVA) with Bonferroni’s multiple comparisons test for post-hoc test. Survival analysis was performed using the log-rank (Mantel-Cox) test. Statistical significance was defined as *P* < 0.05. ns (not significant), *P* > 0.05; *P < 0.05, **P < 0.01, ***P < 0.001.

## Results

### CARD9 ablation facilitates acute kidney injury and ferroptosis during disseminated candidiasis

To decode the molecular mechanism by which CARD9 mediates antifungal immunity, *Card9*^−/−^ mice and WT mice were used to establish disseminated candidiasis model. Histopathology examination indicated elevated renal inflammation, renal injury and fungal burden in the kidneys of *Card9*^−/−^ mice after *C. tropicalis* infection (Fig. S1A and B). In addition, *Card9*^−/−^ mice had reduced numbers of MDSCs in the spleen, kidney and bone marrow (BM) compared with WT mice (Fig. S1C and D). However, we found comparable proportion of neutrophils, macrophages and DCs in the kidney between *C. tropicalis*-infected *Card9*^−/−^ and WT mice, which was further confirmed by immunohistochemical analysis (Fig. S1E and F). Disseminated candidiasis causes systemic inflammation resembling sepsis and kidney damage [36, 42]. Therefore, we detected soluble triggering receptor expressed on myeloid cells (TREM1), a sepsis marker and the indicators of acute kidney injury, neutrophil gelatinase-associated lipocalin (NGAL), serum creatinine (sCr) and blood urea nitrogen (BUN) in serum of mice. Compared to WT mice, the levels of TREM1, NGAL, sCr and BUN were significantly increased in serum of *Card9*^−/−^ mice (Fig. S1G).

**Fig. 1:**
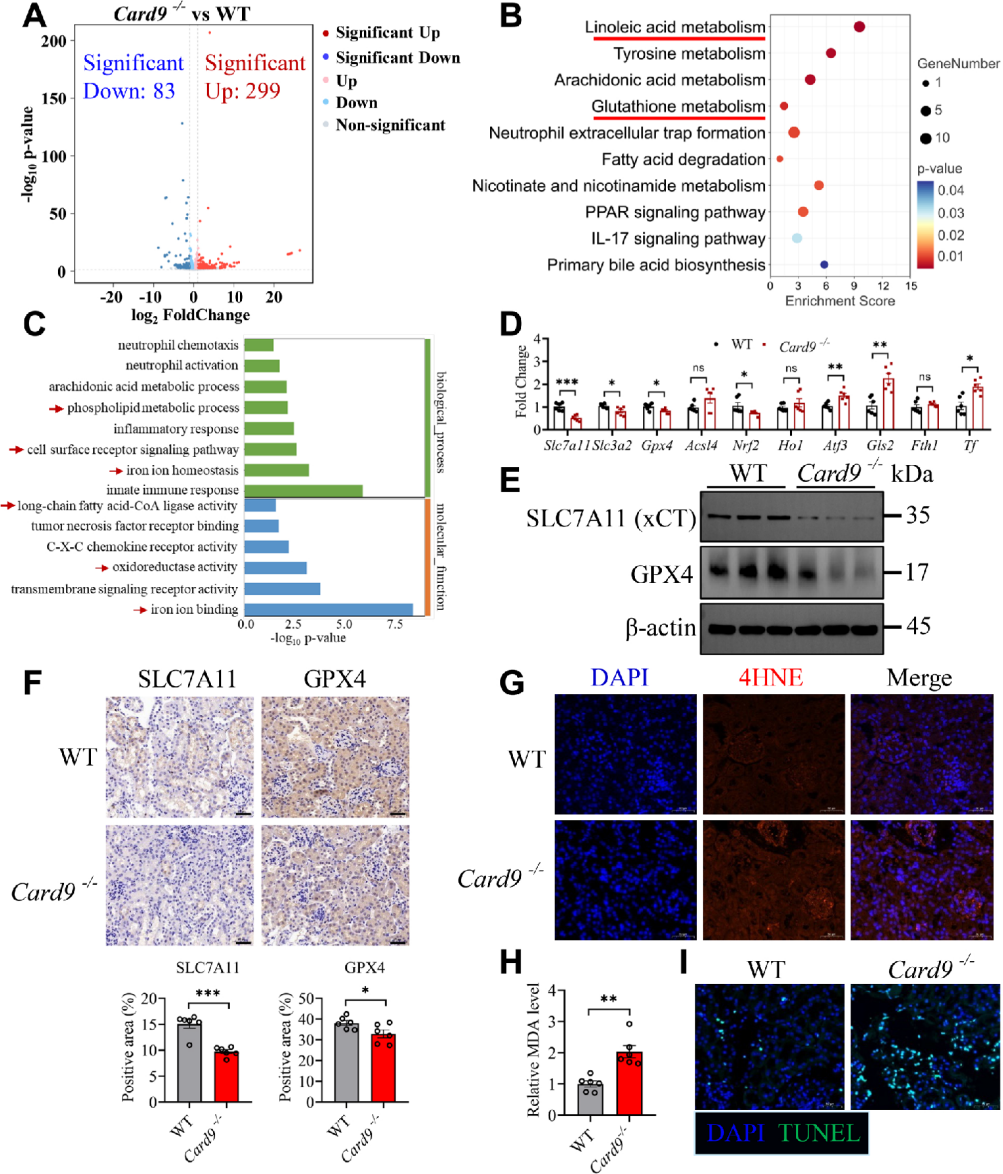
CARD9 ablation promotes acute kidney injury and renal ferroptosis during disseminated candidiasis. **(A-C)** RNA-sequencing analysis was performed with the kidneys of WT and *Card9*^−/−^ mice (*n* = 6 for each group) infected with *C. tropicalis*. The volcano map showed all the differentially expressed genes **(A)**. KEGG and GO pathway enrichment analysis **(B** and **C)**. **(D)** The expression of the indicated genes in the kidneys of WT and *Card9*^−/−^ mice infected with *C. tropicalis* were detected by qPCR. **(E)** Western blot analysis of SLC7A11 and GPX4 in the kidneys of WT and *Card9*^−/−^ mice infected with *C. tropicalis*. **(F)** The protein levels of SLC7A11 and GPX4 in infected kidneys were measured by IHC. Scale bar: 50 µM. The percentages of SLC7A11-positive and GPX4-positive were calculated. **(G)** 4HNE protein expression in infected kidneys was determined by immunofluorescence. 4HNE shown in red. DAPI (blue) was used to stain the nuclei. Scale bar: 50 µM. **(H)** The relative MDA level in infected kidneys were detected by MDA Assay Kit. **(I)** The cell death in infected kidneys was determined by TUNEL staining. TUNEL staining shown in green. DAPI (blue) was used to stain the nuclei. Scale bar: 50 µM. Data with error bars are expressed as mean ± SEM, *n* = 6. Each panel shows six independent biological replicates from a typical experiment. Statistical analysis was determined by two-way ANOVA with Bonferroni’s multiple comparisons test for post-hoc test **(D)** and the unpaired Student’s *t*-test **(F** and **H)**. ns (not significant), *P* > 0.05; *P < 0.05, **P < 0.01, ***P < 0.001

Next, RNA-sequencing analysis was performed on the kidneys of WT and *Card9*^−/−^ mice infected with *C. tropicalis*. Compared with WT mice, the expression of 83 genes were significantly downregulated and 299 genes were significantly upregulated in *Card9*^−/−^ mice (Fig. 1A). KEGG and GO pathway enrichment analysis indicated that the differentially expressed genes were mainly enriched in glutathione metabolism, iron ion homeostasis, oxidoreductase activity, iron ion binding, linoleic acid metabolism, long-chain fatty acid-CoA ligase activity, cell surface receptor signaling pathway and phospholipid metabolic process (Fig. 1B and C), suggesting that CARD9 deficiency potentially resulted in ferroptosis and peroxidation in the kidneys. To verify above results, we measured the expression of proteins associated with ferroptosis in the kidneys. We found that the expression of *Slc7a11*, *Slc3a2*, *Gpx4* and *Nrf2* mRNA were reduced, while the expression of *Atf3*, *Gls2* and transferrin (*Tf*) mRNA were increased in *Card9*^−/−^ mice compared with WT mice (Fig. 1D). Among them, *Slc7a11* revealed the most notable distinction in mRNA expression. Furthermore, immunoblotting and IHC showed that CARD9 deficiency resulted in decreased protein levels of SLC7A11 and GPX4 (Fig. 1E and F). SLC7A11 still showed the greatest difference in protein levels. Furthermore, a significant elevation in 4HNE, MDA and cell death was observed in the kidneys of *Card9*^−/−^ mice (Fig. 1G-I). In conclusion, our findings manifest that CARD9 plays an essential role in suppressing acute kidney injury and ferroptosis during disseminated candidiasis.

### CARD9-deficient MDSCs are susceptible to ferroptosis upon stimulation with *C. tropicalis*

As above results suggested that CARD9 deficiency reduced the accumulation of MDSCs in the kidney and promoted renal ferroptosis during *C. tropicalis* infection, we hypothesized that CARD9 deficiency might facilitate ferroptosis of MDSCs. Therefore, we first analyzed previously published data on RNA-sequencing and metabolomics [8]. It was found that *C. tropicalis* stimulation markedly upregulated the expression of *Slc7a11* and *Slc3a2* genes in MDSCs under in vitro condition, a finding that qPCR validated (Fig. S2A and C). And the levels of intermediate metabolites of glutathione metabolism, Cysteinylglycine, γ-glutamylcysteine, glutathione (GSH) and oxidized glutathione (GSSG) were also increased in MDSCs after *C. tropicalis* stimulation (Fig. S2B). Immunoblotting analysis further validated that *C. tropicalis* increased the protein level of SLC7A11 in MDSCs (Fig. S2D).

**Fig. 2:**
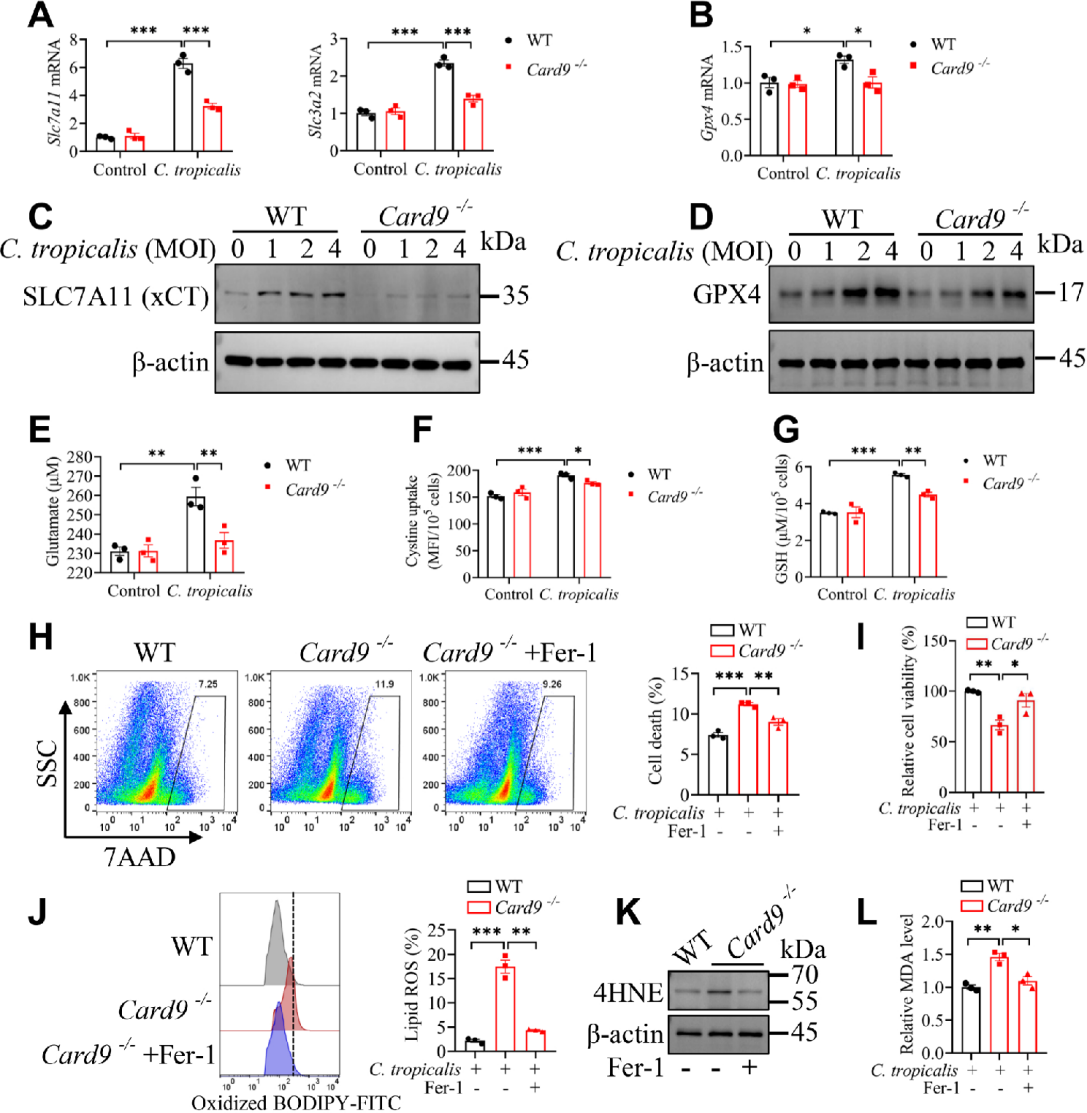
Decreased SLC7A11 expression augments the susceptibility of CARD9-deficient MDSCs to ferroptosis during *C. tropicalis* stimulation. **(A and B)** WT and *Card9*^−/−^ MDSCs were stimulated with *C. tropicalis* (MOI = 1) for 6 h. The expression of *Slc7a11*, *Slc3a2* and *Gpx4* mRNA were detected by qPCR. **(C and D)** WT and *Card9*^−/−^ MDSCs were stimulated with *C. tropicalis* (MOI = 1, 2, 4) for 24 h. The protein levels of SLC7A11 and GPX4 were measured by western blot. **(E-G)** WT and *Card9*^−/−^ MDSCs were stimulated with *C. tropicalis* (MOI = 1) for 24 h. The glutamate in medium supernatant, intracellular cystine and GSH were determined by the corresponding assay kit. **(H-L)** WT and *Card9*^−/−^ MDSCs were stimulated with *C. tropicalis* (MOI = 1), meanwhile, only CARD9-deficient MDSCs were treated with vehicle (DMSO) or ferrostatin-1 (Fer-1, 10 µM). After 24 h, the proportion of dead MDSCs was measured by 7AAD **(H)**. The relative cell viability of MDSCs was detected by CCK-8 kit **(I)**. The Lipid ROS of MDSCs was measured by BODIPY 581/591 C11 staining **(J)**. The protein level of 4HNE was determined by western blot **(K)**. The relative MDA level in MDSCs was detected by MDA Assay Kit **(L)**. Data with error bars are expressed as mean ± SEM, *n* = 3. Each panel shows at least three independent biological replicates from a typical experiment. Statistical analysis was determined by one-way or two-way ANOVA with Bonferroni’s multiple comparisons test for post-hoc test. ns (not significant), *P* > 0.05; *P < 0.05, **P < 0.01, ***P < 0.001

To define how CARD9 regulate glutathione metabolism and ferroptosis in MDSCs during *C. tropicalis* stimulation, WT and CARD9-deficient BM-derived MDSCs were stimulated with *C. tropicalis*. The qPCR assay revealed that CARD9-deficient MDSCs exhibited lower expression of *Slc7a11*, *Slc3a2* and *Gpx4* than WT MDSCs upon *C. tropicalis* stimulation (Fig. 2A and B). We further confirmed that CARD9 deficiency resulted in reduced SLC7A11 and GPX4 protein levels in MDSCs stimulated with *C. tropicalis*(Fig. 2C and D). It was worth noting that consistent with the results in vivo, SLC7A11 also displayed the most notable distinction in expression. We also discovered that *C. tropicalis* observably increased the levels of extracellular glutamate, cystine uptake and GSH, and deficiency of CARD9 in MDSCs absolutely abolished these effects **(**Fig. 2E-G). To verify whether deletion of CARD9 induces cell death of MDSCs through ferroptosis, WT and CARD9-deficient MDSCs were stimulated with *C. tropicalis*, meanwhile, only CARD9-deficient MDSCs were treated with or without ferrostatin-1 (Fer-1), an inhibitor of ferroptosis. We found that CARD9 deletion augmented the cell death of MDSCs, lipid ROS production, 4HNE expression and MDA level, which was rescued by Fer1 (Fig. 2H-L). Taken together, the data suggest that CARD9 block ferroptosis of MDSCs by upregulating SLC7A11 during *C. tropicalis* stimulation.

### CLRs-CARD9-FosB signaling axis manipulates SLC7A11 expression

During fungal recognition, C-type lectin receptors (CLRs) triggers downstream signaling pathways, activating Syk-PKCδ-CARD9 and non-classical Raf-1 signaling pathways [11, 13, 43]. Therefore, we used *Clec4d* (Dectin-3) gene knockout to eliminate the effect of Dectin-3, and selective pharmacological inhibitors to block Dectin-1, Syk, PKCδ, and Raf-1, respectively in MDSCs. As shown in Fig. S3A, *Clec4d* gene knockout and inhibitors of Dectin-1, Syk and PKCδ impaired upregulated transcription of *Slc7a11* induced by *C. tropicalis*. However, inhibition of Raf-1 had no effect on *Slc7a11* transcription. We also measured the protein level of SLC7A11 and phosphorylation of PKCδ. MDSCs stimulated with *C. tropicalis* indicated elevated protein level of SLC7A11 and phosphorylation of PKCδ compared with the untreated group (Fig. S3B). *Clec4d* gene knockout and inhibiting Dectin-1, Syk and PKCδ abolished the expression of SLC7A11 and phosphorylation of PKCδ induced by *C. tropicalis*(Fig. S3B-E). Consistently, Raf-1 inhibitor did not influence the expression of SLC7A11 (Fig. S3F).

**Fig. 3:**
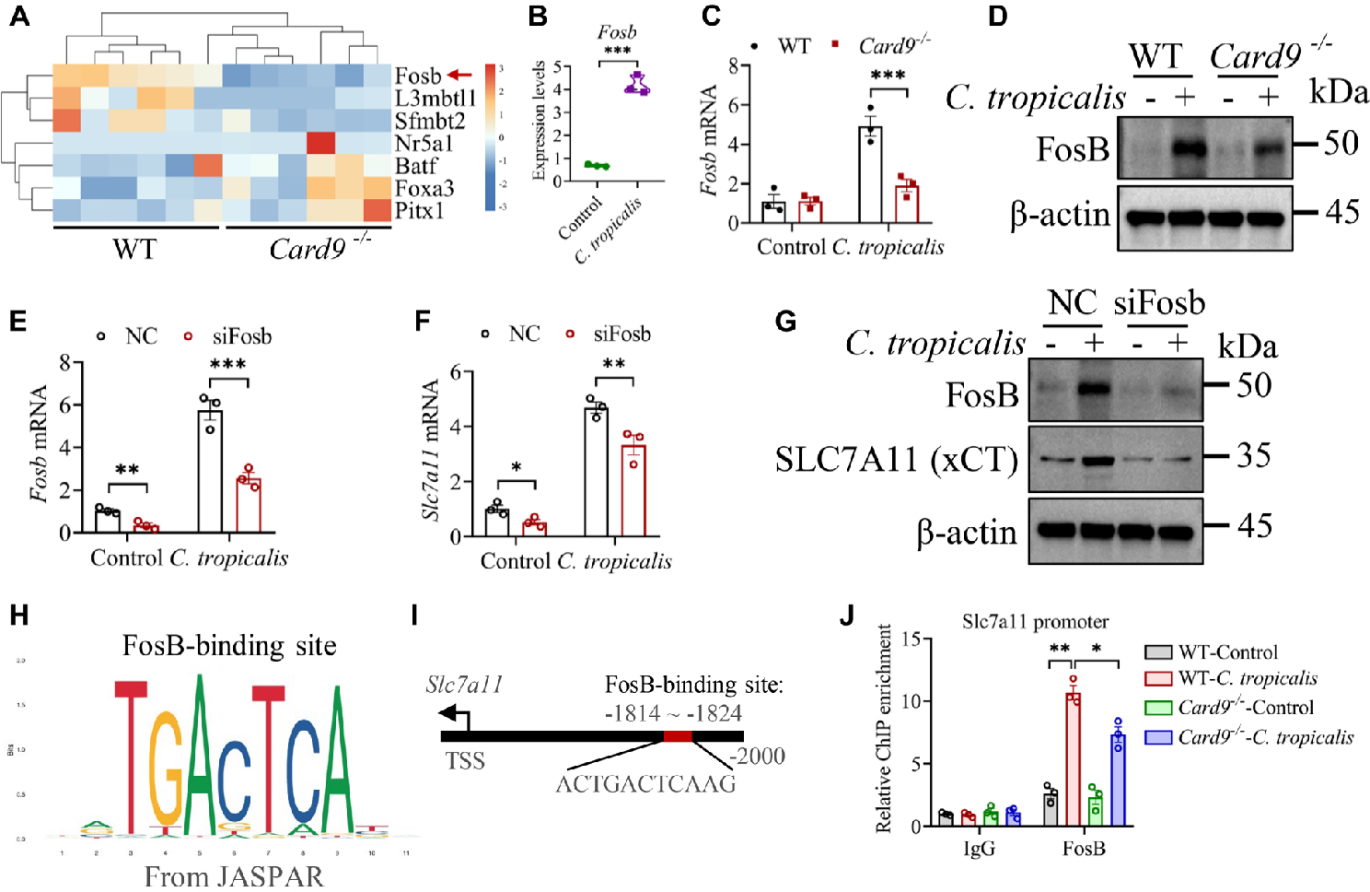
FosB controls*Slc7a11* transcription in MDSCs during *C. tropicalis*stimulation. (A) Heat map demonstrating differentially expressed genes in the kidneys from *Card9*^−/−^ mice infected with *C. tropicalis* compared with WT mice. **(B)** The expression levels of *Fosb* gene in MDSCs treated with or without *C. tropicalis* for 6 h from RNA-sequencing analysis. **(C)** WT and *Card9*^−/−^ MDSCs were stimulated with *C. tropicalis* (MOI = 1) for 6 h. The expression of *Fosb* mRNA was detected by qPCR. **(D)** WT and *Card9*^−/−^ MDSCs were stimulated with *C. tropicalis* (MOI = 1) for 24 h. The protein level of FosB was measured by western blot. **(E-G)** WT MDSCs were transfected with Fosb specific siRNA for 24 h, then the cells were stimulated with or without *C. tropicalis* (MOI = 1) for 6 h **(E and F)** or 24 h **(G)**. The expression of FosB and SLC7A11 were detected by qPCR and western blot assays. **(H)** The logo of FosB-binding site from the JASPAR CORE database (https://jaspar.genereg.net/). **(I)** One potential FosB-binding site in the *Slc7a11* promoter were predicted by using the JASPAR CORE database. **(J)** WT and *Card9*^−/−^ MDSCs were stimulated with or without *C. tropicalis* (MOI = 1) for 6 h. ChIP-qPCR analysis of the interaction between FosB and *Slc7a11* promoter binding site. Data with error bars are expressed as mean ± SEM, **(A**, *n* = 6**) (B-G and J**, *n* = 3**)**. Each panel shows at least six or three independent biological replicates from a typical experiment. Statistical analysis was determined by the unpaired Student’s *t*-test and two-way ANOVA with Bonferroni’s multiple comparisons test for post-hoc test. ns (not significant), *P* > 0.05; *P < 0.05, **P < 0.01, ***P < 0.001

The above results suggest that *C. tropicalis* stimulates transcription of *Slc7a11* through CLRs-CARD9 signaling pathway. Therefore, we speculate that there may be transcription factors downstream of CARD9 that regulate *Slc7a11* transcription. To validate this speculation, we further analyzed our RNA-sequencing data. All differentially expressed transcription factors in the kidneys of *Card9*^−/−^ mice were screened by cluster analysis compared with WT mice. Interestingly, *Fosb* was significantly down-regulated in *Card9*^−/−^ mice compared with WT mice and displayed the most notable distinction (Fig. 3A). Moreover, we also found that *C. tropicalis* markedly upregulated the expression of *Fosb* in MDSCs by analyzing previously published data on RNA-sequencing (Fig. 3B). FosB is a member of the Fos family and commonly coupled with c-Jun to generate the AP-1 transcription factor complex [44]. FosB plays a key role in enhancing the ability of AP-1 to bind DNA [45]. To further confirm the above results, WT and CARD9-deficient MDSCs were stimulated with or without *C. tropicalis*. The qPCR and immunoblotting assay revealed that CARD9 deficiency markedly impaired *C. tropicalis*-induced expression of FosB (Fig. 3C and D). Next, we further determined whether FosB regulates the expression of SLC7A11 in MDSCs. First, the mRNA and protein expression of FosB in MDSCs stimulated with or without *C. tropicalis* was effectively knocked down by using siRNA (Fig. 3E and G). Then, we found that siRNA of Fosb caused considerable decrease of the mRNA and protein expression of SLC7A11 induced by *C. tropicalis* (Fig. 3F and G). Furthermore, bioinformatic analysis indicated that *Slc7a11* gene promoter exhibited one putative FosB-binding site (Fig. 3H and I). In consequence, ChIP-qPCR assays demonstrated a marked interaction between FosB and *Slc7a11* gene promoter, which was further enhanced by the stimulation of *C. tropicalis* (Fig. 3J). However, CARD9 deficiency significantly attenuated *C. tropicalis*-induced interaction between FosB and *Slc7a11* gene promoter (Fig. 3J). Hence, the above results unveil that CLRs-CARD9-FosB signaling axis manipulates SLC7A11 expression during *C. tropicalis* stimulation.

### CARD9 deficiency enhances oxidative phosphorylation in MDSCs during *C. tropicalis* stimulation

**Fig. 4:**
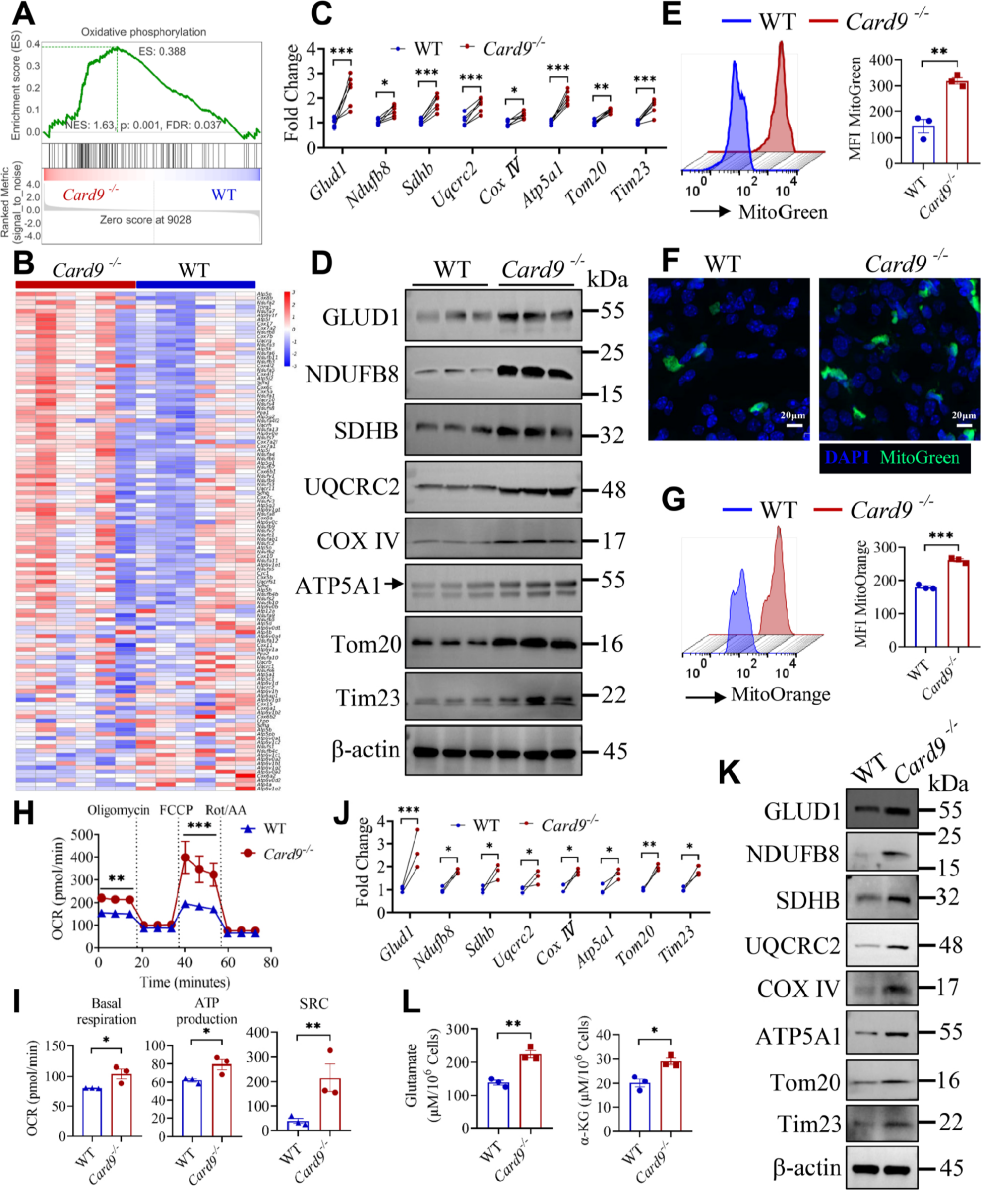
CARD9 controls OXPHOS and mitochondrial metabolism in MDSCs during *C. tropicalis* stimulation. **(A)** The Gene Set Enrichment Analysis (GSEA) diagram revealed oxidative phosphorylation pathway in infected kidneys. **(B)** Heat map showing the investigation of the gene expression for oxidative phosphorylation in infected kidneys of *Card9*^−/−^ and WT mice. **(C)** qPCR analysis of the mitochondrial gene in the kidneys of WT and *Card9*^−/−^ mice infected with *C. tropicalis*. **(D)** Western blot analysis of the mitochondrial proteins in the kidneys of WT and *Card9*^−/−^ mice infected with *C. tropicalis*. **(E-G)** WT and *Card9*^−/−^ MDSCs were stimulated with *C. tropicalis* (MOI = 1) for 24 h. Mitochondrial function and mass were measured by flow cytometry assay **(E)** and immunofluorescence, scale bar: 20 µM **(F)**. Mitochondrial membrane potential was assessed by a flow cytometry assay **(G)**. **(H)** Oxygen consumption rate (OCR) of WT and *Card9*^−/−^ MDSCs stimulated with *C. tropicalis* (MOI = 1) for 24 h was detected by Seahorse Cell Mito Stress Test. **(I)** Basal respiration, ATP production and spare respiratory capacity (SRC) were calculated based on Seahorse Cell Mito Stress Test. **(J)** qPCR analysis of the mitochondrial genes in WT and *Card9*^−/−^ MDSCs treated with *C. tropicalis* (MOI = 1) for 6 h. **(K and L)** WT and *Card9*^−/−^ MDSCs were stimulated with *C. tropicalis* (MOI = 1) for 24 h. The mitochondrial proteins were measured by Western blot analysis **(K)**. And the levels of intracellular glutamate and α-KG were detected by assay kit **(L)**. Data with error bars are expressed as mean ± SEM, **(A-D**, *n* = 6**) (E-L**, *n* = 3**)**. Each panel shows at least six or three independent biological replicates from a typical experiment. Statistical analysis was determined by two-way ANOVA with Bonferroni’s multiple comparisons test for post-hoc test and the unpaired Student’s *t*-test. ns (not significant), *P* > 0.05; *P < 0.05, **P < 0.01, ***P < 0.001

We then attempted to elucidate the molecular mechanism by which CARD9 deficiency promotes ferroptosis of MDSCs during *C. tropicalis* stimulation. Therefore, we further analyzed the RNA-sequencing data from infected kidneys. Surprisingly, Gene Set Enrichment Analysis (GSEA) revealed that genes associated with oxidative phosphorylation (OXPHOS) were markedly enriched in *Card9*^−/−^ mice compared to WT mice (Fig. 4A and B). Then we further verified the above results. Glutamate dehydrogenase 1 (GLUD1) converts intracellular glutamate into α-ketoglutarate (α-KG), a key intermediate in the TCA cycle, thereby fueling the TCA cycle. Consistently, qPCR and immunoblotting assay showed that the expression of GLUD1 and the mitochondrial proteins NDUFB8, SDHB, UQCRC2, COX IV, ATP5A1, Tom20, Tim23 were significantly increased in the kidneys of *Card9*^−/−^ mice (Fig. 4C and D). To decipher whether CARD9 plays a vital role in modulating mitochondrial metabolism of MDSCs upon stimulation with *C. tropicalis in vivo*, we detected mitochondrial function and mass by MitoTracker Green and assessed mitochondrial membrane potential by MitoTracker Orange. We discovered a considerable rise in mitochondrial mass (Fig. 4E and F) and mitochondrial membrane potential (Fig. 4G) in CARD9-deficient MDSCs compared with WT MDSCs during *C. tropicalis* stimulation. Furthermore, CARD9-deficient MDSCs exhibited higher Oxygen Consumption Rate (OCR) than WT MDSCs after stimulation with *C. tropicalis* (Fig. 4H). Basal respiration, spare respiratory capacity (SRC) and ATP production were significantly increased in CARD9-deficient MDSCs (Fig. 4I). We also observed that CARD9 deficiency markedly promoted the mRNA and protein expression of GLUD1 and the mitochondrial proteins in MDSCs after *C. tropicalis* stimulation (Fig. 4J and K). And CARD9-deficient MDSCs had higher intracellular levels of glutamate and α-KG in comparison to WT MDSCs upon *C. tropicalis* stimulation (Fig. 4L). These findings show conclusively that loss of CARD9 promotes OXPHOS and mitochondrial metabolism in MDSCs upon *C. tropicalis* stimulation.

### SLC7A11 controls OXPHOS of MDSCs upon *C. tropicalis* stimulation

**Fig. 5:**
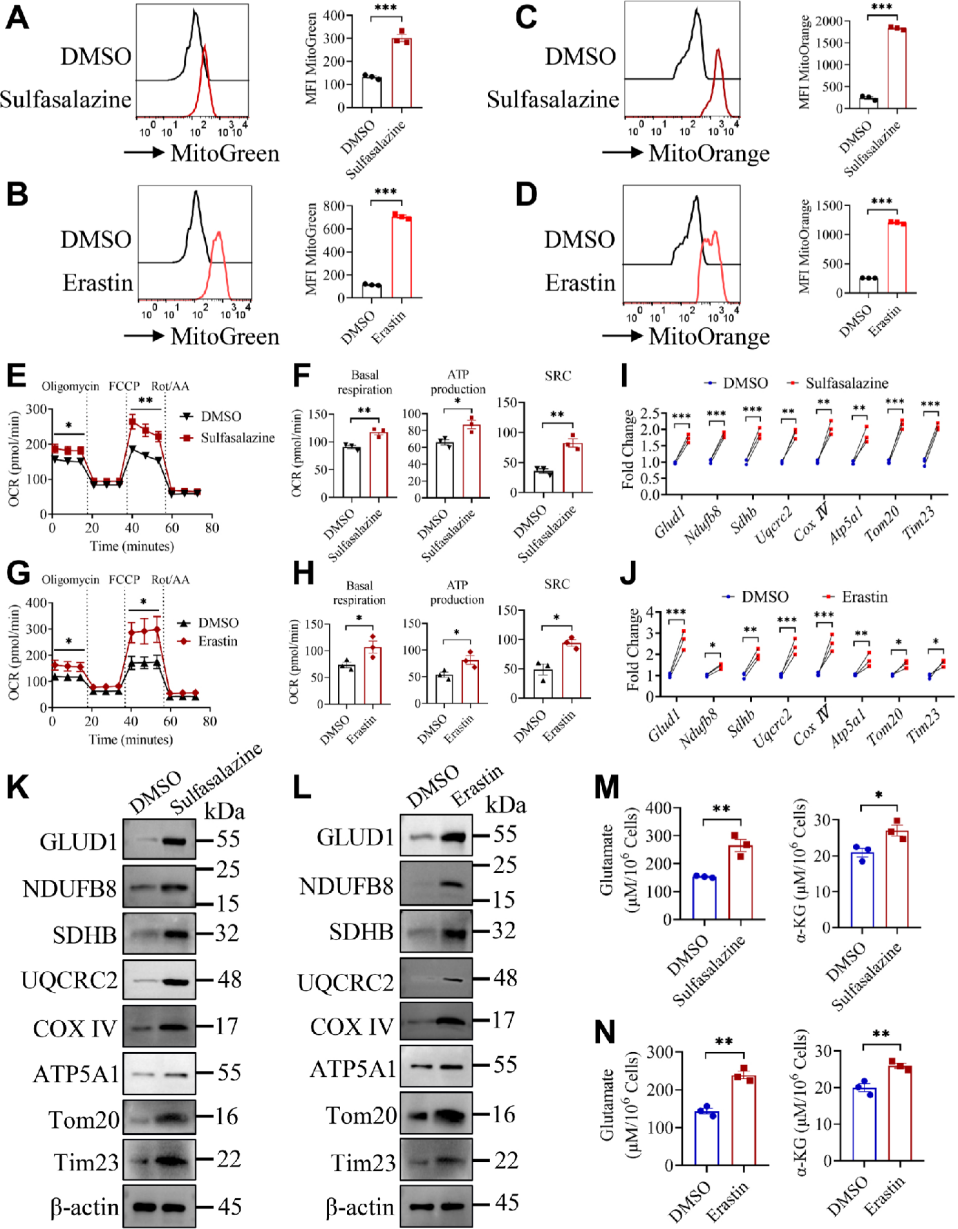
Inhibition of SLC7A11 promotes OXPHOS of MDSCs upon *C. tropicalis* stimulation. **(A-H, K-N)** WT MDSCs were stimulated with *C. tropicalis* (MOI = 1) in the presence or absence of Sulfasalazine (1mM) or Erastin (5 µM) for 24 h. Mitochondrial function and mass, mitochondrial membrane potential were assessed by a flow cytometry assay **(A-D)**. Oxygen consumption rate (OCR) of MDSCs was detected by Seahorse Cell Mito Stress Test **(E and G)**. Basal respiration, ATP production and spare respiratory capacity (SRC) were calculated based on Seahorse Cell Mito Stress Test **(F and H)**. The indicated proteins were measured by Western blot analysis **(K and L)**. The levels of intracellular glutamate and α-KG were detected by assay kit **(M and N)**. **(I and J)** qPCR analysis of the indicated genes in WT MDSCs treated with *C. tropicalis* (MOI = 1) in combination with or without Sulfasalazine (1mM), Erastin (5 µM) for 6 h. Data with error bars are expressed as mean ± SEM, *n* = 3. Each panel shows at least three independent biological replicates from a typical experiment. Statistical analysis was determined by two-way ANOVA with Bonferroni’s multiple comparisons test for post-hoc test and the unpaired Student’s *t*-test. ns (not significant), *P* > 0.05; *P < 0.05, **P < 0.01, ***P < 0.001

To investigate whether OXPHOS of MDSCs induced by CARD9 ablation is attributable to decreased expression of SLC7A11, we cultured WT MDSCs and treated them with *C. tropicalis* in combination with or without Sulfasalazine and Erastin, the SLC7A11 inhibitors. The SLC7A11 inhibitors significantly augmented mitochondrial mass and mitochondrial membrane potential of MDSCs (Fig. 5A-D). These findings indicate that SLC7A11 may modulate mitochondrial metabolism. Analysis of bioenergetic profiles in real-time suggested that OCR and basal respiration, SRC, ATP production were markedly elevated in sulfasalazine or Erastin-treated MDSCs (Fig. 5E-H). In accordance, SLC7A11 inhibitors-treated MDSCs presented higher mRNA and protein expression of mitochondrial proteins in comparison to control (Fig. 5I-L). Moreover, intracellular glutamate and α-KG levels were also significantly increased in MDSC after treatment with inhibitors of SLC7A11 (Fig. 5M and N). To further confirm that inhibition of SLC7A11 results in increased OXPHOS of MDSCs, WT MDSCs were pretransfected with Slc7a11 siRNA and then stimulated with *C. tropicalis*. We first verified that the siRNA of Slc7a11 could effectively knock down the expression of SLC7A11 by qPCR and western blot analysis (Fig. S4A and B). Consistent with the above results, we obtained similar results that Slc7a11 knockdown enhanced OXPHOS of MDSCs (Fig. S4C-I). Next, we used adenovirus to transfect Slc7a11-overexpressing plasmid into CARD9-deficient MDSCs. Then these cells were treated with *C. tropicalis*. We first examined the overexpression efficiency of Slc7a11 by immunoblotting and found that the expression of SLC7A11 was dramatically elevated in *Card9*^−/−^ MDSCs after transfection with Slc7a11-overexpressing adenovirus vector (Fig. S5A). Moreover, we discovered that SLC7A11 overexpression significantly blocked the increase of mitochondrial OXPHOS in CARD9-deficient MDSCs (Fig. S5B-I). Overall, these data indicate that SLC7A11 controls OXPHOS of MDSCs upon *C. tropicalis* stimulation.

### Mitochondrial OXPHOS promotes ferroptosis of MDSCs upon *C. tropicalis* stimulation

**Fig. 6:**
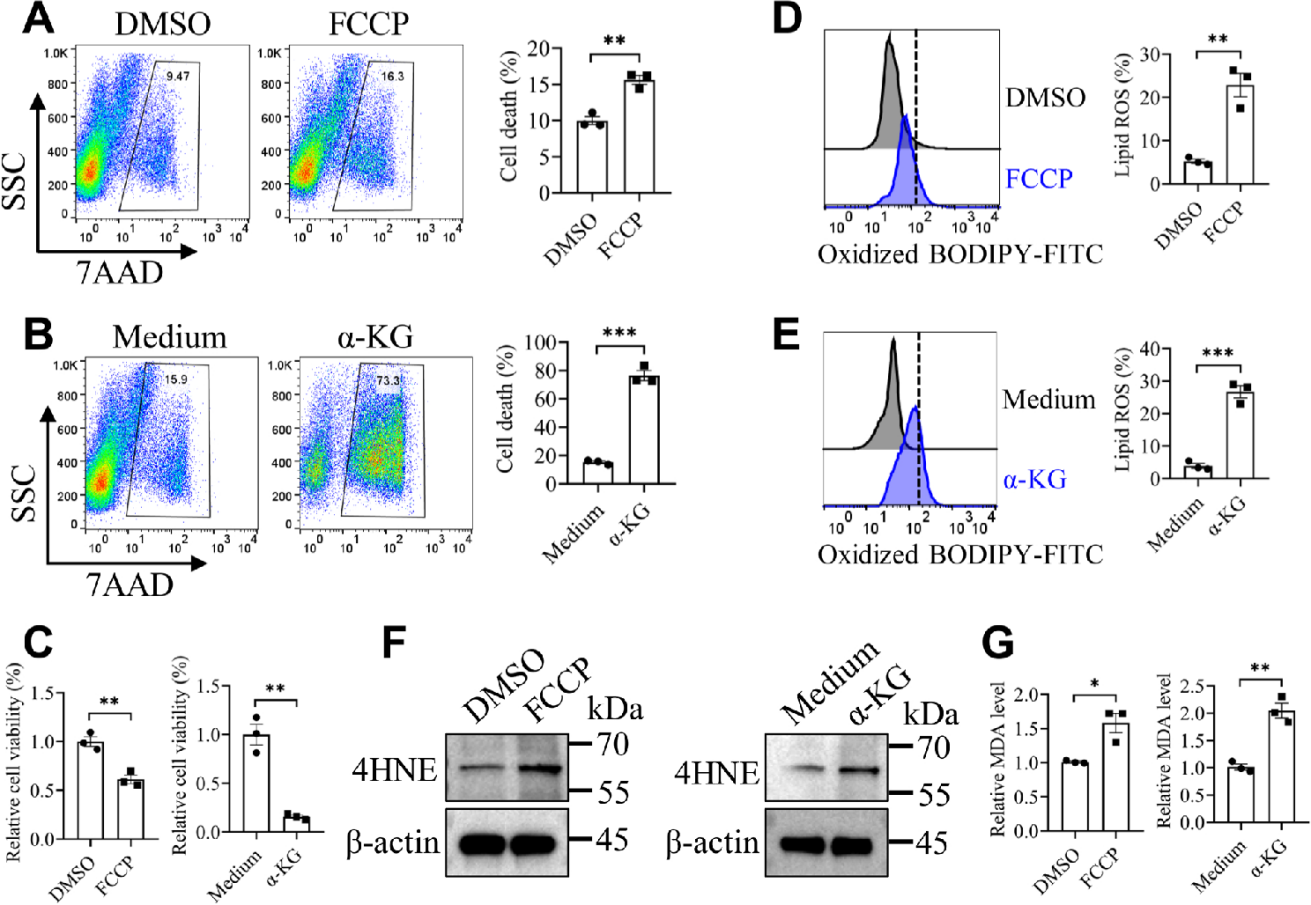
Mitochondrial OXPHOS facilitates ferroptotic cell death in *C. tropicalis*-stimulated MDSCs. WT MDSCs were stimulated with *C. tropicalis* (MOI = 1) in the presence or absence of FCCP (5 µM) or Dimethyl 2-oxoglutarate (α-KG) (8 mM) for 24 h. **(A and B)** The percentage of cell death was determined using 7AAD. **(C)** The relative cell viability was detected by CCK-8 kit. **(D and E)** The Lipid ROS production was measured by BODIPY 581/591 C11 staining followed by flow cytometry. **(F)** The protein level of 4HNE was determined by western blot. **(G)** The relative MDA level was detected by MDA Assay Kit. Data with error bars are expressed as mean ± SEM, *n* = 3. Each panel shows at least three independent biological replicates from a typical experiment. Statistical analysis was determined by the unpaired Student’s t-test. ns (not significant), *P* > 0.05; *P < 0.05, **P < 0.01, ***P < 0.001

Mitochondrial OXPHOS supported by the TCA and ETC has been reported to augment the production of ROS, involving ferroptosis-inducing lipid ROS. Hence, mitochondrial metabolic function is involved in ferroptosis during cysteine deprivation and SLC7A11 inhibition [46, 47]. To further clarify whether mitochondria play an essential role in ferroptosis of MDSCs stimulated by *C. tropicalis*, WT MDSCs were pretreated by mitochondrial OXPHOS uncoupler Carbonyl cyanide 4-(trifluoromethoxy) phenylhydrazone (FCCP) or α-KG, which facilitates mitochondrial OXPHOS. These cells were then further stimulated with *C. tropicalis*. First, we did find that α-KG-treated MDSCs exhibited higher mitochondrial OXPHOS activity evidenced by increased OCR, basal respiration, SRC and ATP production (Fig. S6A and B). The results showed that FCCP and α-KG significantly promoted the cell death of MDSCs and decreased the cell viability of MDSCs (Fig. 6A-C). Compared to the control group, lipid peroxidation was elevated in FCCP- and α-KG-pretreated MDSCs (Fig. 6D and E). In line with these findings, FCCP- and α-KG-pretreated MDSCs had greater amounts of 4HNE and MDA than the control group (Fig. 6F and G). These findings indicate that activation of mitochondrial OXPHOS augments ferroptotic cell death of MDSCs. Because we found that CARD9 ablation led to elevated ferroptosis and OXPHOS in MDSCs. Therefore, we hypothesized that CARD9 ablation exacerbated ferroptosis of MDSCs that could be attributed to the enhancement of OXPHOS. To confirm this hypothesis, CARD9-deficient MDSCs were pretreated with the ATP synthase inhibitor oligomycin or the mitochondrial ETC complex I inhibitor metformin [48–50], which could prevent OXPHOS and then stimulated with *C. tropicalis*. Indeed, metformin inhibited mitochondrial OXPHOS of CARD9-deficient MDSCs stimulated by *C. tropicalis* (Fig. S6C and D). Treatment with oligomycin and metformin attenuated CARD9 ablation-induced increases in cell death, lipid ROS, 4HNE and MDA levels of MDSCs (Fig. S6E-K). In summary, our results highlight the critical contribution of mitochondrial OXPHOS to the enhancement of ferroptosis in *C. tropicalis*-stimulated MDSCs.

### CARD9 ablation-aggravated renal injury and ferroptosis are attributed to mitochondrial OXPHOS during disseminated *C. tropicalis* infection

**Fig. 7:**
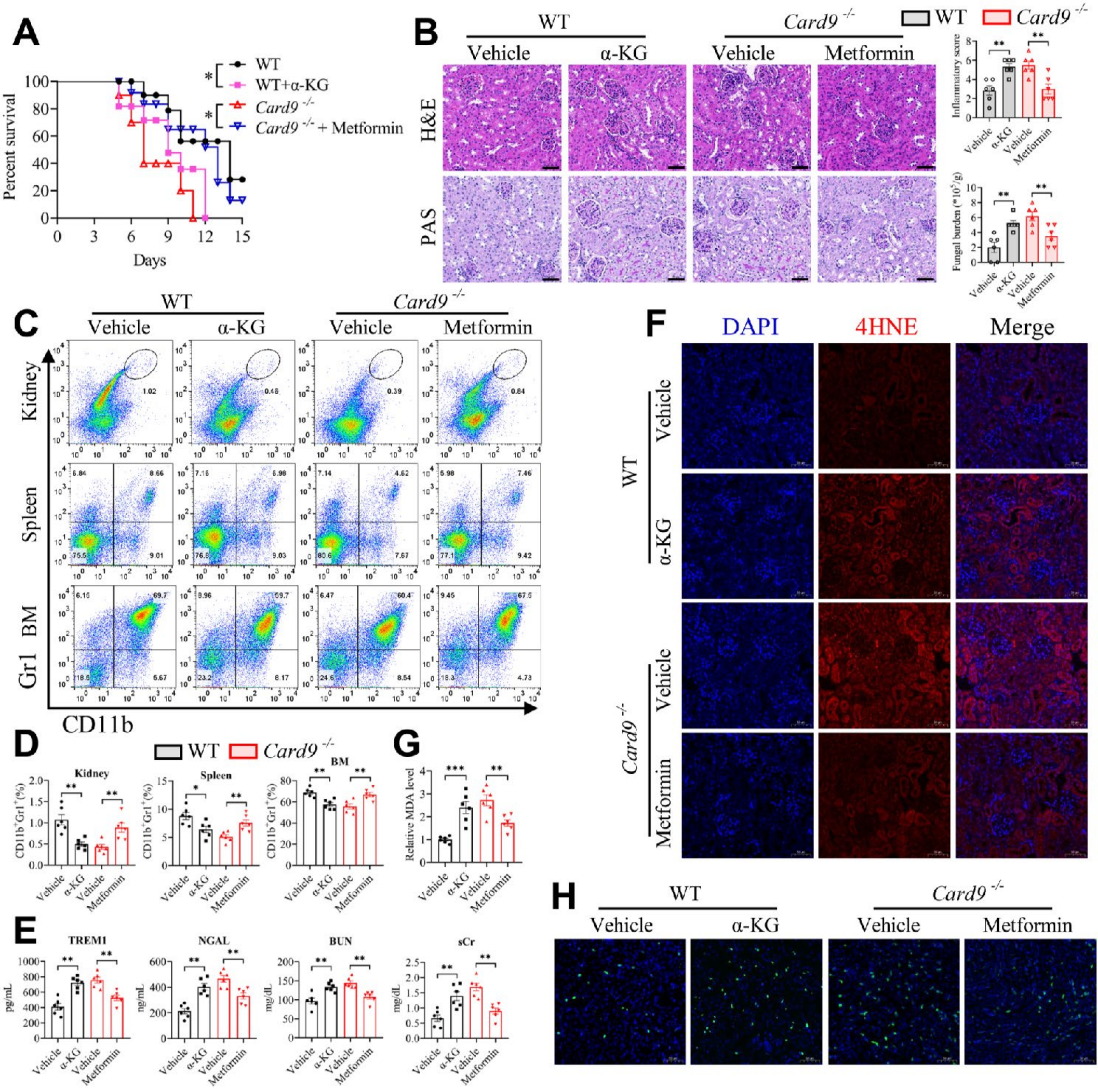
Renal injury and ferroptosis aggravated by CARD9 ablation are caused by OXPHOS during disseminated *C. tropicalis* infection. To create disseminated candidiasis, *Card9*^−/−^ and WT mice were intravenously infected with *C. tropicalis* (2×10^5^ CFU per mouse). In the meantime, WT mice were intraperitoneally injected with PBS (Vehicle) or α-KG (500 mg/kg) per day. And *Card9*^−/−^ mice were treated with PBS (Vehicle) or metformin (200 mg/kg) by oral gavage daily. **(A)** The mice were monitored daily for survival postinfection (*n* = 8). **(B-H)** Mice were euthanized at day 5 (*n* = 6). Representative photographs of kidney slices stained with hematoxylin and eosin (H&E) and Periodic Acid-Schiff (PAS). Scale bar: 50 µM. And the inflammatory score and fungal burden was also calculated **(B)**. The proportion of MDSCs (CD11b^+^Gr1^+^) in kidney, spleen and bone marrow (BM) were determined by flow cytometry **(C and D)**. The levels of serum TREM1, NGAL, BUN and sCr were measured by the corresponding assay kit **(E)**. The protein level of 4HNE in infected kidneys was detected by immunofluorescence. 4HNE shown in red. DAPI (blue) was used to stain the nuclei. Scale bar: 50 µM **(F)**. The relative renal MDA level was measured by MDA Assay Kit **(G)**. The cell death in infected kidneys was determined by TUNEL staining. TUNEL staining shown in green. DAPI (blue) was used to stain the nuclei. Scale bar: 50 µM **(H)**. Data with error bars are expressed as mean ± SEM. Each panel shows at least six independent biological replicates from a typical experiment. Statistical analysis was determined by the log-rank (Mantel-Cox) test **(A)** and one-way ANOVA with Bonferroni’s multiple comparisons test for post-hoc test **(B-G)**. ns (not significant), *P* > 0.05; *P < 0.05, **P < 0.01, ***P < 0.001

To elucidate whether mitochondrial OXPHOS is implicated in the enhancement of acute kidney injury and ferroptosis induced by CARD9 deletion during disseminated candidiasis, we established invasive *C. tropicalis* infection model by using WT mice and *Card9*^−/−^ mice. During invasive *C. tropicalis* infection, WT mice were treated with α-KG and *Card9*^−/−^ mice were treated with metformin daily. The results showed that α-KG treatment significantly reduced the survival rate of *C. tropicalis*-infected WT mice and metformin treatment markedly increased the survival rate of CARD9-deficient mice infected with *C. tropicalis* (Fig. 7A). Histological analysis indicated that renal inflammation, injury and fungal burden were elevated in α-KG-treated WT mice compared with vehicle control and metformin significantly reversed renal inflammation, injury, and fungal burden exacerbated by CARD9 deletion (Fig. 7B). Furthermore, the proportion of MDSCs in the spleen, kidney and BM were notably decreased in WT mice treated with α-KG in comparison with that in vehicle control, and metformin also restored the decline in MDSCs caused by CARD9 deletion (Fig. 7C and D). And the serum TREM1, NGAL, sCr and BUN levels were significantly augmented in α-KG-treated WT mice and attenuated in metformin-treated *Card9*^−/−^ mice (Fig. 7E). We then examined the effect of OXPHOS on renal ferroptosis. Therefore, we first detected the expression of SLC7A11 and GPX4 in the kidneys. We found that treatment with α-KG markedly reduced the mRNA and protein expression of SLC7A11 and GPX4 in WT mice, and treatment with metformin significantly enhanced the mRNA and protein expression of SLC7A11 and GPX4 in *Card9*^−/−^ mice, which was further verified by immunohistochemical staining (Fig. S7A-C). Moreover, the levels of 4HNE and MDA (the hallmarks of lipid peroxidation), cell death in the kidneys of α-KG-treated WT mice were higher than those of vehicle control (Fig. 7F-H). Additionally, metformin significantly attenuates these markers of ferroptosis raised by CARD9 ablation (Fig. 7F-H). Conclusively, these data confirm that mitochondrial OXPHOS is involved in the augmented acute kidney injury and ferroptosis induced by CARD9 deletion during disseminated candidiasis.

### Ferroptosis inhibition alleviates renal damages and ferroptosis exacerbated by CARD9 deficiency during *C. tropicalis* infection

**Fig. 8:**
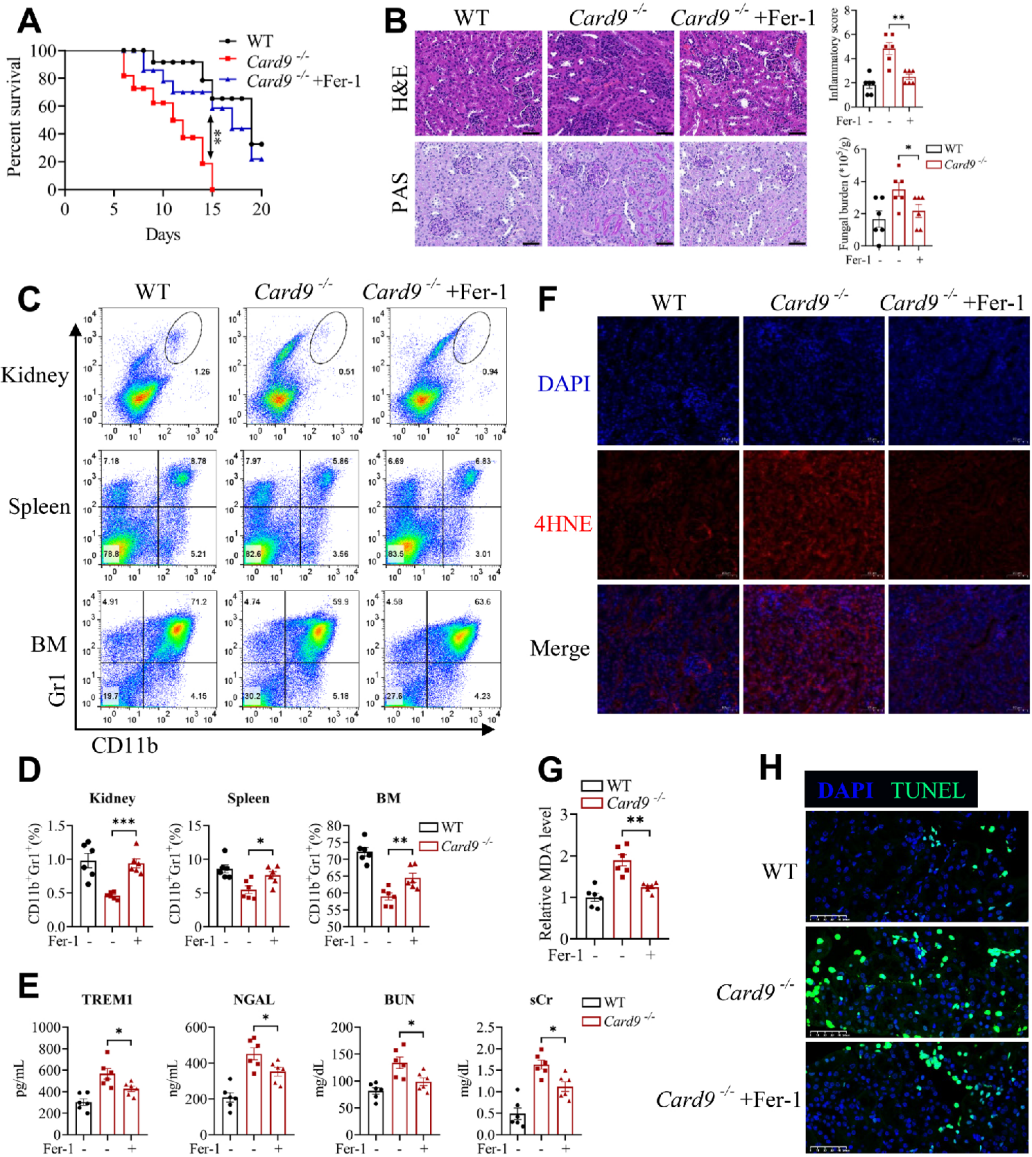
Pharmacological inhibition of ferroptosis mitigates CARD9 deficiency- exacerbated renal damages and ferroptosis during *C. tropicalis* infection. *Card9*^−/−^ and WT mice were injected with *C. tropicalis* (2×10^5^ CFU per mouse) via the lateral tail veins to establish disseminated candidiasis. Meanwhile, *Card9*^−/−^ mice were intraperitoneally injected with DMSO (Vehicle) or ferrostatin-1 (Fer-1, 10 mg/kg) every day. **(A)** The mice were monitored daily for survival postinfection (*n* = 8). **(B-H)** Mice were euthanized at day 5 (*n* = 6). Representative photographs of kidney slices stained with hematoxylin and eosin (H&E) and Periodic Acid-Schiff (PAS). Scale bar: 50 µM. And the inflammatory score and fungal burden was also calculated **(B)**. The percentage of MDSCs (CD11b^+^Gr1^+^) in kidney, spleen and bone marrow (BM) were analyzed by flow cytometry **(C and D)**. The levels of serum TREM1, NGAL, BUN and sCr were assessed by the corresponding assay kit **(E)**. The protein level of 4HNE in infected kidneys was detected by immunofluorescence. 4HNE shown in red. DAPI (blue) was used to stain the nuclei. Scale bar: 50 µM **(F)**. The relative renal MDA level was measured by MDA Assay Kit **(G)**. The cell death in infected kidneys was determined by TUNEL staining. TUNEL staining shown in green. DAPI (blue) was used to stain the nuclei. Scale bar: 50 µM **(H)**. Data with error bars are expressed as mean ± SEM. Each panel shows at least six independent biological replicates from a typical experiment. Statistical analysis was determined by the log-rank (Mantel-Cox) test **(A)** and one-way ANOVA with Bonferroni’s multiple comparisons test for post-hoc test **(B-H)**. ns (not significant), *P* > 0.05; *P < 0.05, **P < 0.01, ***P < 0.001

The above results highlighted that ferroptosis was implicated in reduced antifungal immunity and renal injury caused by CARD9 deficiency during disseminated candidiasis. Therefore, we further explored whether pharmacological inhibition of ferroptosis mitigates renal injury and ferroptotic cell death in CARD9-deficient mice infected with *C. tropicalis*. *Card9*^−/−^ mice and WT mice were infected intravenously with *C. tropicalis*. Meanwhile, *Card9*^−/−^ mice were intraperitoneally injected the ferroptosis inhibitor, ferrostatin-1 (Fer-1) every day. The mice were assessed for survival postinfection daily. We found that *Card9*^−/−^ mice treated with Fer-1 revealed greater resistance to *C. tropicalis* infection than vehicle control *Card9*^−/−^ mice (Fig. 8A). Histological analysis suggested less renal inflammation, injury and fungal burden in Fer-1-treated *Card9*^−/−^ mice in comparison with that in vehicle control *Card9*^−/−^ mice (Fig. 8B). In addition, treatment with Fer-1 recovered the proportion of MDSCs in the spleen, kidney and BM of CARD9-deficient mice (Fig. 8C and D). Compared with those in vehicle control *Card9*^−/−^ mice, serum TREM1, NGAL, sCr and BUN levels were decreased in Fer-1-treated *Card9*^−/−^ mice (Fig. 8E). We also detected 4HNE and MDA, the lipid peroxides, as well as cell death in the kidneys. As expected, we discovered that Fer-1 significantly reduced CARD9 ablation-caused increases in the levels of 4HNE, MDA and cell death in the kidneys (Fig. 8F-H). This further validated that CARD9 deficiency aggravated renal injury and reduced antifungal immunity by enhancing renal ferroptosis during *C. tropicalis* infection. In a word, these results imply that ferroptosis inhibition relieves renal damages and ferroptosis in CARD9-deficient mice infected with *C. tropicalis*.

## Discussion

CARD9 is known to play an essential role in antifungal immunity. Most studies have shown that CARD9 in myeloid cells participates in host resistance against invasive *Candida* infection [13, 17, 18, 22, 40, 51, 52]. CARD9 deletion results in impaired fungal killing ability of myeloid cells, causing a systemic fungal infection. Rieber et al. show that MDSCs defend against invasive *Candida* infection in 2015 [26]. However, few studies have identified whether CARD9 mediates antifungal immunity by regulating the expansion of MDSCs. The present study demonstrates that CARD9 deletion exacerbates acute kidney injury in invasive *C. tropicalis* infection due to a decrease in the number of MDSCs. However, the trigger for the reduction of MDSCs in the kidneys of CARD9-deficient mice infected with *C. tropicalis* remains largely unknown. Recent evidence has uncovered that ferroptotic myeloid cells are implicated in renal immunopathology of systemic candidiasis [36]. Therefore, our current study shows that CARD9 deficiency results in elevated ferroptosis in the kidneys during *C. tropicalis* infection, which is attributed to decreased SLC7A11 expression. Of note, our data also demonstrate that CARD9-deficient MDSCs exhibit a substantially lower expression of SLC7A11, resulting in significantly increased ferroptosis under the stimulation of *C. tropicalis*. However, the ferroptosis inhibitor, ferrostatin-1 inhibits ferroptosis of MDSCs caused by CARD9 deficiency. Furthermore, renal damages and ferroptosis are also suppressed by ferrostatin-1 during *C. tropicalis* infection, although the mice remain CARD9 deficient. And ferrostatin-1 also recovers the number of MDSCs in CARD9-deficient mice infected with *C. tropicalis*. This further elucidates that CARD9 deficiency promotes ferroptosis of MDSCs by decreasing SLC7A11 expression in the kidney, leading to aggravated kidney damage during *C. tropicalis* infection. Our previous studies have indicated that *C. tropicalis* treatment results in increased MDSCs, especially increased PMN-MDSCs in vitro [27]. In this study, by using IL-6 and GM-CSF, we induce the formation of MDSCs by bone marrow cells in vitro. And in our previous study, we have examined the percentage of M-MDSCs and PMN-MDSCs in the induced total MDSCs, showing that the percentage of PMN-MDSCs is much larger than the percentage of M-MDSCs [8]. A recent study has also revealed that PMN-MDSCs in the tumor microenvironment are susceptible to ferroptosis [37]. Therefore, the evidence is sufficient to suggest that PMN-MDSCs, but not M-MDSCs, can undergo ferroptosis in the present study. This deserves further detailed investigation. A previous study has shown that pathogenic fungi *C. albicans* induce the generation of PMN-MDSCs through the Dectin-1/CARD9 signaling pathway, thereby modulating immunity [26]. Thus, we infer that PMN-MDSCs may also undergo ferroptosis in *C. albicans* infection, which remains to be studied and is also the focus of our next research. This would be very important for the fields of medical mycology and immunology.

MDSCs not only play a crucial role in *C. tropicalis* infection, but also play a paradoxical effect in other microbial infections, such as tuberculosis (TB), cryptococcal or aspergillosis infections. Large amounts of MDSCs have been reported to accumulate in mouse models of TB, and targeting MDSCs is a promising immunotherapy strategy for TB [53, 54]. A recent study has suggested that *Cryptococcus neoformans* infection promotes the recruitment of PMN-MDSCs, to play a harmful role. And targeted inhibition of arginase-1 in MDSCs protects against *Cryptococcus neoformans* infection [55]. One study has reported that PMN-MDSCs are involved in *Aspergillus fumigatus* infection by inhibiting NK cells [56].

Several signaling pathways and transcription factors have been reported to be implicated in the regulation of SLC7A11 expression. Previous research has demonstrated that ferroptosis is primarily modulated by NRF2, which plays an antioxidant role by controlling SLC7A11 transcription [57]. Emerging evidence further clarifies that ATF4 and NRF2 coordinately promote transcription of SLC7A11, which block stress-related ferroptosis by controlling glutathione metabolism [58]. In contrast, ATF3 augments ferroptosis by transcriptionally negatively regulating SLC7A11 expression [59]. Here, we indicate that *C. tropicalis* recognition via CLRs signaling pathways is indispensable for SLC7A11expression in MDSCs. However, how CARD9 regulates the transcription of SLC7A11 has not been studied so far. Thus, for the first time, we find that CARD9 controls the transcription of SLC7A11 via the transcription factor FosB which can bind to the SLC7A11 gene promoter, thereby promoting SLC7A11 transcription in *C. tropicalis*-stimulated MDSCs. It has been reported that the oncogene FosB is able to interact with HIF-1α, thereby promoting HIF-1α binding to glycolytic targets [60]. Moreover, FosB interacts with c-Jun to form AP-1 transcription factor complex, promoting cell proliferation.

Metabolic reprogramming of immune cells is a fundamental factor of immune function, which has attracted great attention in recent years. So far, most immunometabolism studies have focused on neutrophils and macrophages. For instance, glycolysis of neutrophils is necessary for antifungal ability [13]. And the latest evidence suggests that the lack of CARD9 causes mitochondrial dysfunction to produce more mitochondrial reactive oxygen species (mtROS), promoting neutrophil apoptosis [61]. However, it is poorly understood how CARD9 regulates metabolic reprogramming of MDSCs and how metabolic reprogramming of MDSCs regulates antifungal activity. Indeed, we discover that CARD9 ablation results in mitochondria overactivation and elevated OXPHOS in MDSCs upon *C. tropicalis* stimulation, which is also attributable to the lower expression of SLC7A11. In our present study, the mechanism by which SLC7A11 inhibition leads to increased OXPHOS may be that SLC7A11 inhibition blocks the output of intracellular glutamate, resulting in the accumulation of intracellular glutamate. Intracellular glutamate is then converted into α-KG by GLUD1, thus promoting the TCA cycle and OXPHOS in mitochondria. More research is necessary to completely understand the effect of mitochondrial overactivation on MDSC function, particularly in vivo.

Mitochondrial respiration and OXPHOS produce excessive by-product mtROS, which are responsible for ferroptosis [62]. Here, we indicate that activation of OXPHOS contributes to ferroptosis, whereas inhibition of OXPHOS abolishes CARD9 ablation-induced ferroptosis in *C. tropicalis*-stimulated MDSCs. Consistent with our in vitro results, activation of OXPHOS by α-KG promotes acute kidney injury and ferroptosis, whereas inhibition of OXPHOS by metformin reverses acute kidney injury and ferroptosis exacerbated by CARD9 ablation during disseminated candidiasis. Our current study further complements the molecular mechanism by which inhibition of SLC7A11 results in ferroptosis. On the one hand, inhibition of SLC7A11 leads to mitochondrial OXPHOS overactivation, resulting in excessive production of intracellular mtROS; on the other hand, inhibition of SLC7A11 abolishes intracellular antioxidant reaction, and ultimately leads to excessive accumulation of intracellular ROS, inducing ferroptosis.

## Conclusions

In conclusion, our work demonstrates that CARD9 ablation exacerbates acute kidney injury in disseminated candidiasis by enhancing ferroptosis of MDSCs, which is attributed to the lower expression of SLC7A11. SLC7A11, which is transcriptionally controlled by the CLRs-CARD9-FosB axis, suppresses ferroptosis in CARD9-deficient MDSCs by negatively regulating mitochondrial OXPHOS upon *C. tropicalis* stimulation. Inhibitors of mitochondrial metabolism or ferroptosis have therapeutic efficacy on acute kidney injury exacerbated by CARD9 ablation during disseminated candidiasis (Fig. S8).

### Electronic supplementary material

Below is the link to the electronic supplementary material.

Supplementary Material 1

Supplementary Material 2

## Data Availability

The raw data for RNA sequencing in this work have been deposited in the NCBI Sequence Read Archive (SRA) database, which may be accessed using the accession number: PRJNA1053684.

## Abbreviations

CARD9: Caspase Recruitment Domain-containing protein 9
MDSCs: Myeloid-derived suppressor cells
SLC7A11: solute carrier family 7 member 11
OXPHOS: oxidative phosphorylation
CLRs: C-type lectin receptors
GSH: glutathione
GPX4: glutathione peroxidase 4
ROS: reactive oxygen species
4HNE: 4-hydroxynonenal
MDA: malondialdehyde
TCA: tricarboxylic acid cycle
ETC: electron transport chain
TUNEL: Terminal deoxynucleotidyl transferase dUTP nick-end labeling
TREM1: triggering receptor expressed on myeloid cells
NGAL: neutrophil gelatinase-associated lipocalin
BUN: Blood urea nitrogen
sCr: serum creatinine
α-KG: α-ketoglutarate
FCCP: Carbonyl cyanide 4-(trifluoromethoxy) phenylhydrazone
OCR: Oxygen Consumption Rate
Fer-1: ferrostatin-1
ChIP-qPCR: chromatin immunoprecipitation qPCR
