# Supplementary material for: Mitochondria-mediated ferroptosis induced by CARD9 ablation prevents MDSCs-dependent antifungal immunity
Source: Cell Commun Signal. 2024 Apr 2;22:210. doi: 10.1186/s12964-024-01581-2 (PMC10986078; doi:10.1186/s12964-024-01581-2)
Supplement: Supplementary file 1 — Supplementary Material 1 [file 12964_2024_1581_MOESM1_ESM.docx]

**Supplementary Information**

**Supplementary Figures**

**
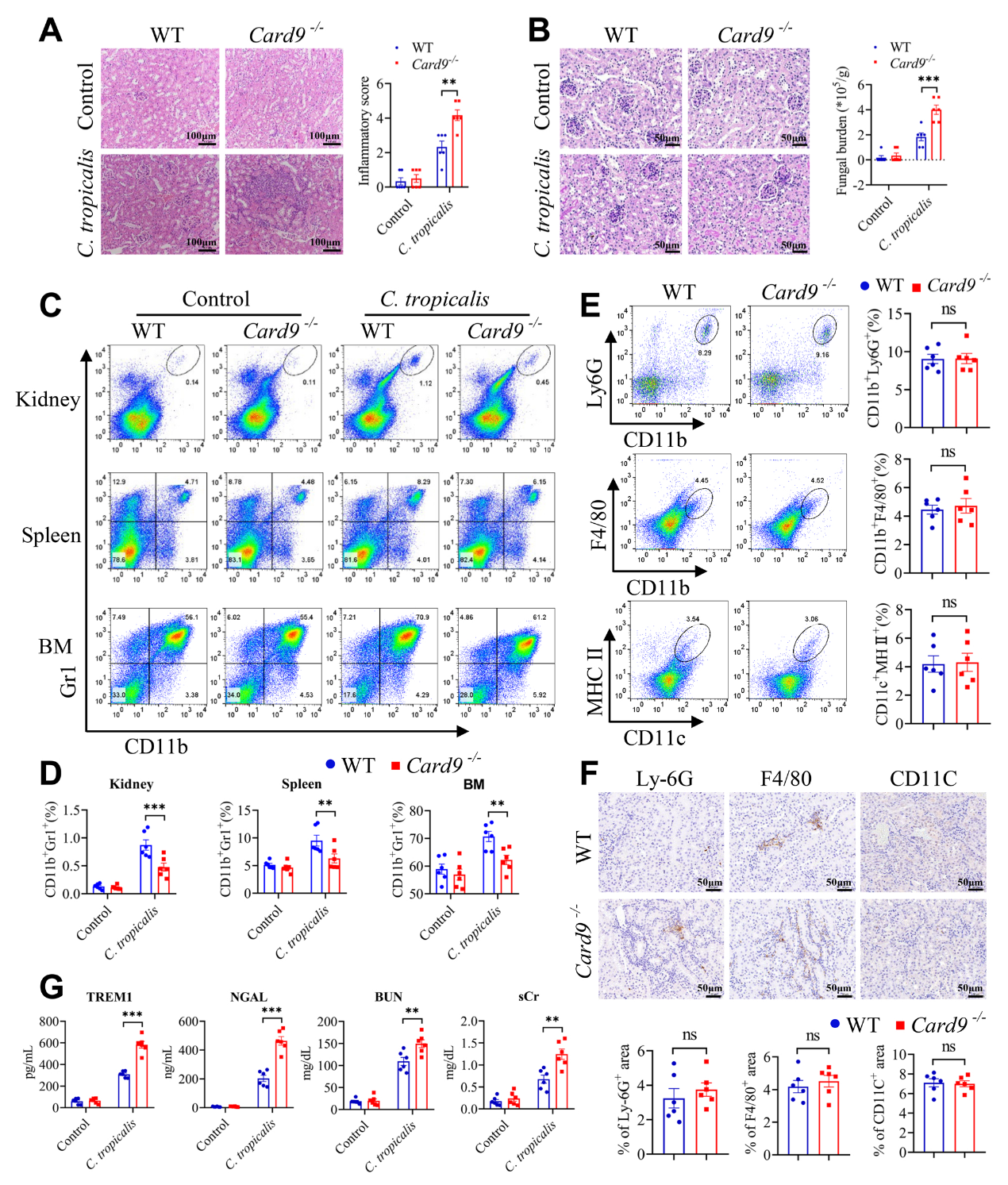
**

**Fig. S1. CARD9 ablation exacerbates acute kidney injury and renal ferroptosis by impairing the accumulation of MDSCs during disseminated candidiasis.** **Related to Fig. 1.** *Card9*^-/-^ (n = 6) and WT (n = 6) mice were injected with *C. tropicalis* (2🞨10^5^ CFU per mouse) via the lateral tail veins. Mice were euthanized at day 5. **(A)** Representative photographs of kidney slices stained with hematoxylin and eosin (H&E). Scale bar: 100 μM. And the inflammatory score was also calculated. **(B)** Representative photographs of kidney slices stained with Periodic Acid-Schiff (PAS). Scale bar: 50 μM. And the fungal burden was shown. **(C and D)** The percentage of MDSCs (CD11b^+^Gr1^+^) in kidney, spleen and bone marrow (BM) were analyzed by flow cytometry. **(E)** The proportion of neutrophils (CD11b^+^ Ly6G^+^), macrophages (CD11b^+^F4/80^+^) and DCs (CD11c^+^MHC Ⅱ^+^) in the kidney from *C. tropicalis*-infected WT and *Card9*^-/-^ mice were detected by flow cytometry analysis. **(F)** Representative images of kidney sections from *C. tropicalis*-infected WT and *Card9*^-/-^ mice stained with Ly-6G (neutrophils marker), F4/80 (macrophages marker) and CD11c (DCs marker). Scale bar: 50 μM. **(G)** The levels of serum TREM1, NGAL, BUN and sCr. Data with error bars are expressed as mean ± SEM, n = 6. Each panel shows six independent biological replicates from a typical experiment. Statistical analysis was determined by two-way ANOVA with Bonferroni's multiple comparisons test for post-hoc test and the unpaired Student’s *t*-test. ns (not significant), P > 0.05; *P < 0.05, **P < 0.01, ***P < 0.001.


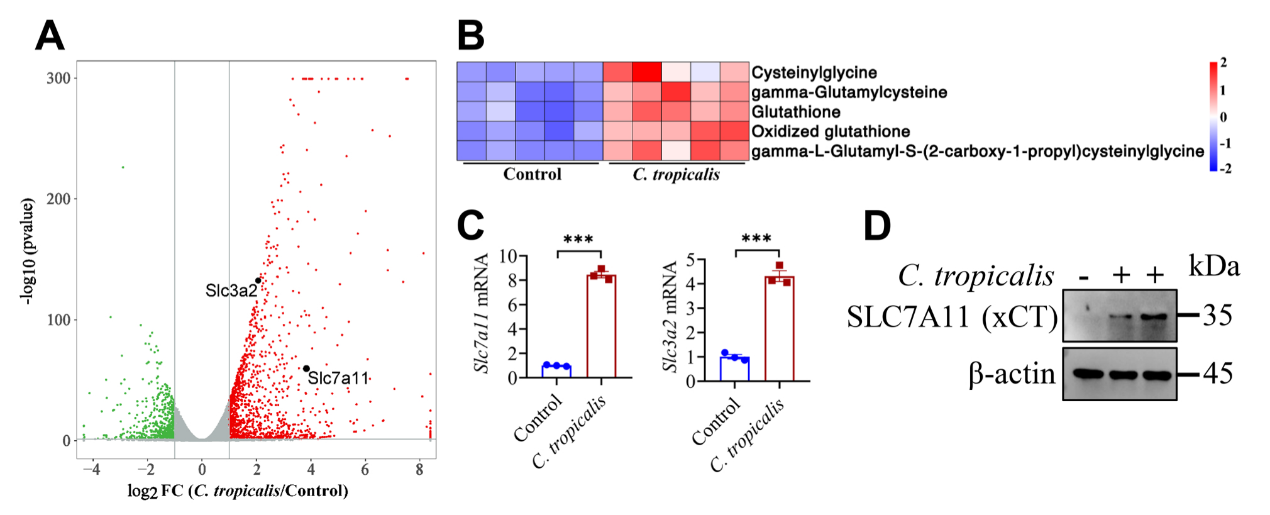


**Fig. S2.** **Decreased SLC7A11 expression augments the susceptibility of CARD9-deficient MDSCs to ferroptosis during *C. tropicalis* stimulation. Related to Fig. 2.** **(A)** BM-derived MDSCs from WT mice were stimulated *C. tropicalis* (MOI = 2) for 6 h. The volcano plot indicated differentially expressed genes (n = 3). **(B)** Unbiased metabolomics analysis of BM-derived MDSCs stimulated with or without *C. tropicalis* (MOI = 1) for 24h. The heat map revealed intermediate metabolites associated with glutathione metabolism (n = 5). **(C)** qPCR analysis of *Slc7a11* and *Slc3a2* mRNA in MDSCs stimulated with or without *C. tropicalis* (MOI = 1) for 6 h (n = 3). **(D)** Western blot analysis of SLC7A11 protein in MDSCs stimulated with or without *C. tropicalis* (MOI = 1, 2) for 24 h (n = 3). Data with error bars are expressed as mean ± SEM. Each panel shows at least three independent biological replicates from a typical experiment. Statistical analysis was determined by the unpaired Student’s t-test. ns (not significant), P > 0.05; *P < 0.05, **P < 0.01, ***P < 0.001.


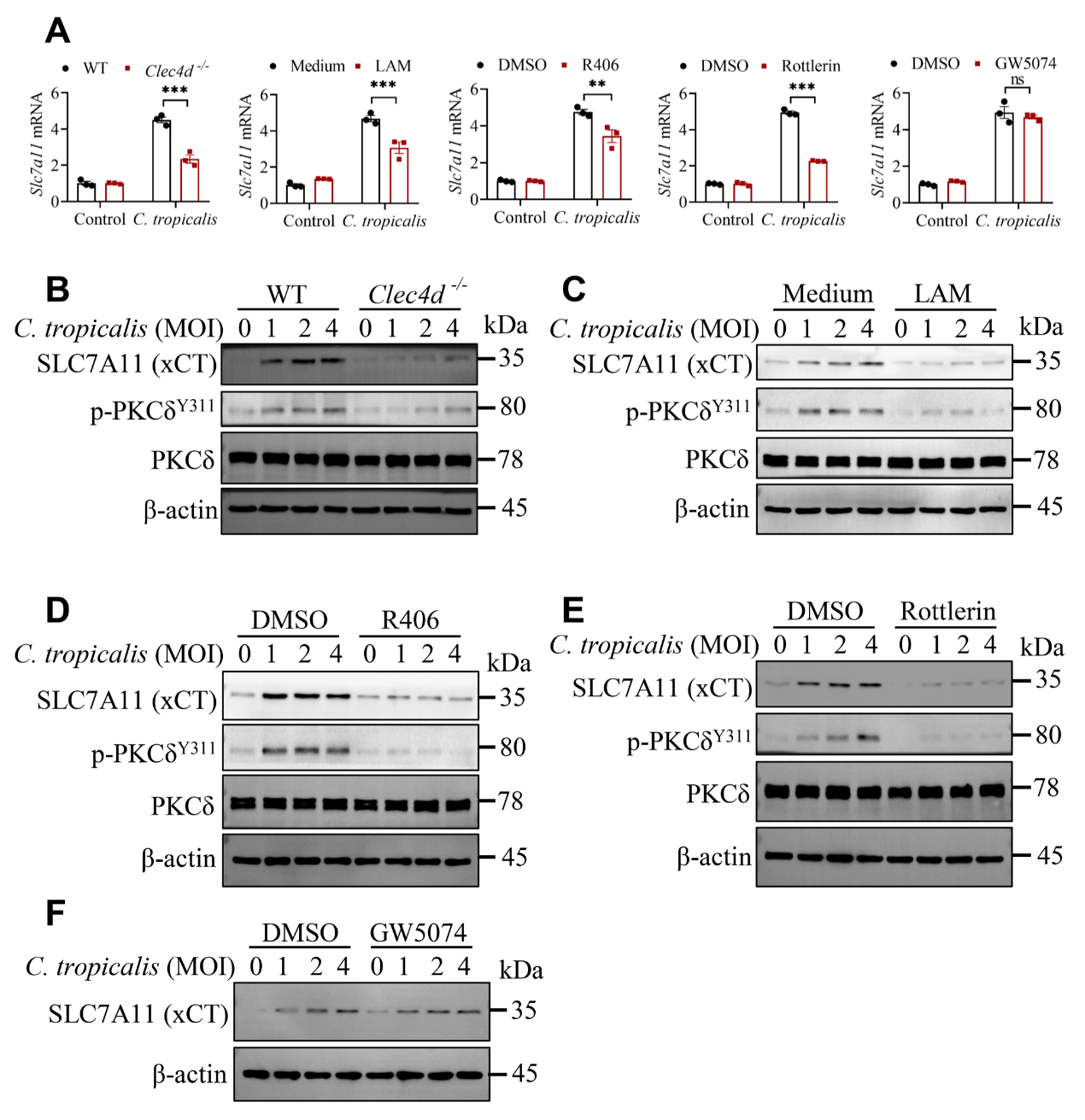


**Fig. S3.** **SLC7A11 regulation is manipulated by CLRs-Syk-PKCδ pathway in MDSCs. Related to Fig. 3. (A)** WT and *Clec4d*^-/-^ MDSCs were stimulated with *C. tropicalis* (MOI = 1) for 6 h. And WT MDSCs were stimulated with *C. tropicalis* (MOI = 1) in combination with or without indicated inhibitors, Laminarin (LAM, Dectin-1 inhibitor, 200 µg/ml), R406 (Syk inhibitor, 1 μM), Rottlerin (PKCδ inhibitor, 50 μM), GW5074 (Raf-1 inhibitor, 10 μM) for 6 h. The expression of *Slc7a11* mRNA was detected by qPCR. **(B-F)** WT and *Clec4d*^-/-^ MDSCs were stimulated with *C. tropicalis* (MOI = 1) for 24 h **(B)**. And WT MDSCs were stimulated with *C. tropicalis* (MOI = 1) in the presence or absence of indicated inhibitors for 6 h **(C-F)**. The expression of the indicated proteins were detected by immunoblotting assay. Data with error bars are expressed as mean ± SEM, n = 3. Each panel shows at least three independent biological replicates from a typical experiment. Statistical analysis was determined by one-way or two-way ANOVA with Bonferroni's multiple comparisons test for post-hoc test. ns (not significant), P > 0.05; *P < 0.05, **P < 0.01, ***P < 0.001.


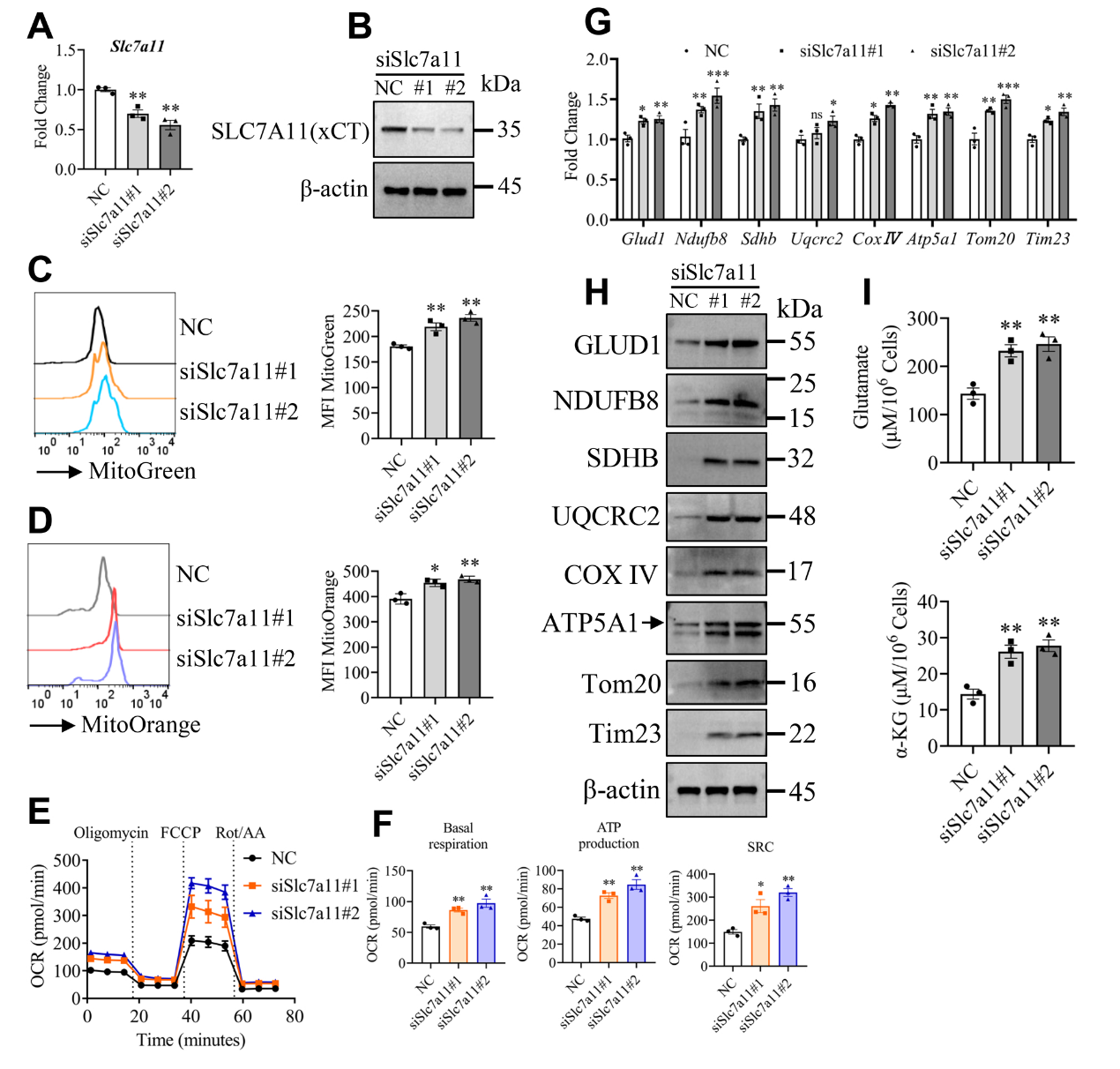


**Fig. S4.** **Inhibition of SLC7A11 promotes OXPHOS of MDSCs upon *C. tropicalis* stimulation. Related to Fig. 5. (A and B)** WT MDSCs were transfected with Slc7a11 siRNA for 24 h, the knockdown efficiency was determined by qPCR and immunoblotting assay. WT MDSCs were transfected with Slc7a11 siRNA or negative control (NC) for 24h, and then treated with *C. tropicalis* (MOI = 1) for 24 h. MitoTracker Green **(C)** and MitoTracker Orange **(D)** were assessed by a flow cytometry assay. Oxygen consumption rate (OCR) of MDSCs was detected by Seahorse Cell Mito Stress Test **(E and F)**. **(G and H)** After transfection with Slc7a11 siRNA, WT MDSCs were treated with *C. tropicalis* (MOI = 1) for 6 h **(G)** or 24 h **(H)**. The mitochondrial proteins were detected by qPCR and immunoblotting assay. **(I)** The levels of intracellular glutamate and α-KG were detected by assay kit. Data with error bars are expressed as mean ± SEM, n = 3. Each panel shows at least three independent biological replicates from a typical experiment. Statistical analysis was determined by one-way or two-way ANOVA with Bonferroni's multiple comparisons test for post-hoc test. ns (not significant), P > 0.05; *P < 0.05, **P < 0.01, ***P < 0.001.


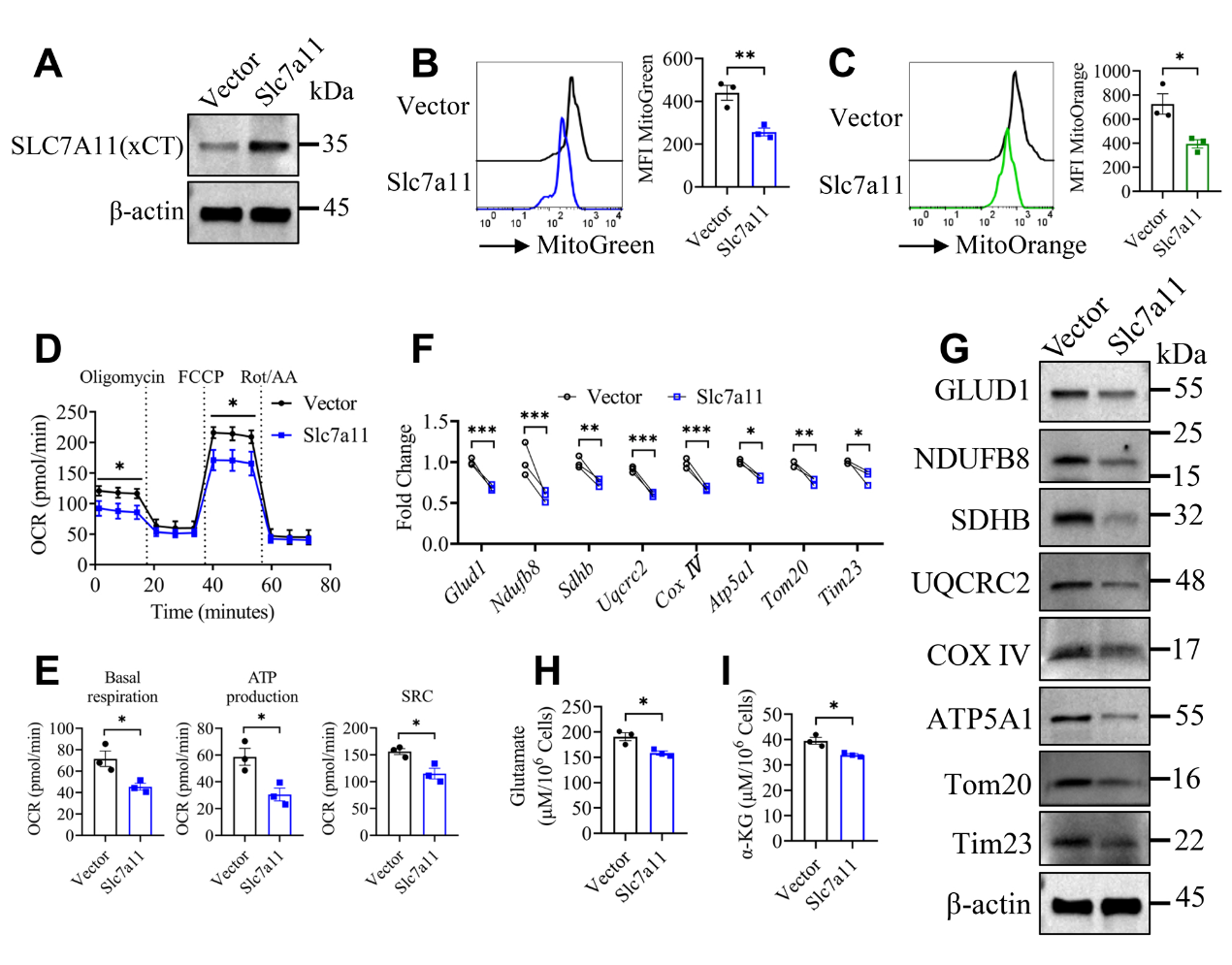


**Fig. S5.** **Forced SLC7A11 expression suppresses CARD9 ablation-induced OXPHOS.** **Related to Fig. 5. (A)** CARD9-deficient MDSCs were transfected with adenovirus containing Slc7a11-overexpressing plasmid. The overexpression efficiency of Slc7a11 was validated by immunoblotting assay. After transfection with Slc7a11-overexpressing adenovirus vector, cells were treated with *C. tropicalis* (MOI = 1) for 24 h. MitoTracker Green **(B)** and MitoTracker Orange **(C)** were assessed by a flow cytometry assay. Oxygen consumption rate (OCR) of MDSCs was detected by Seahorse Cell Mito Stress Test **(D and E)**. CARD9-deficient MDSCs were transfected with Slc7a11-overexpressing adenovirus vector, and then treated with *C. tropicalis* (MOI = 1) for 6 h **(F)** or 24 h **(G)**. The mitochondrial proteins were detected by qPCR and immunoblotting assay. **(H and I)** The levels of intracellular glutamate and α-KG were detected by assay kit. Data with error bars are expressed as mean ± SEM, n = 3. Each panel shows at least three independent biological replicates from a typical experiment. Statistical analysis was determined by two-way ANOVA with Bonferroni's multiple comparisons test for post-hoc test and the unpaired Student’s *t*-test. ns (not significant), P > 0.05; *P < 0.05, **P < 0.01, ***P < 0.001.


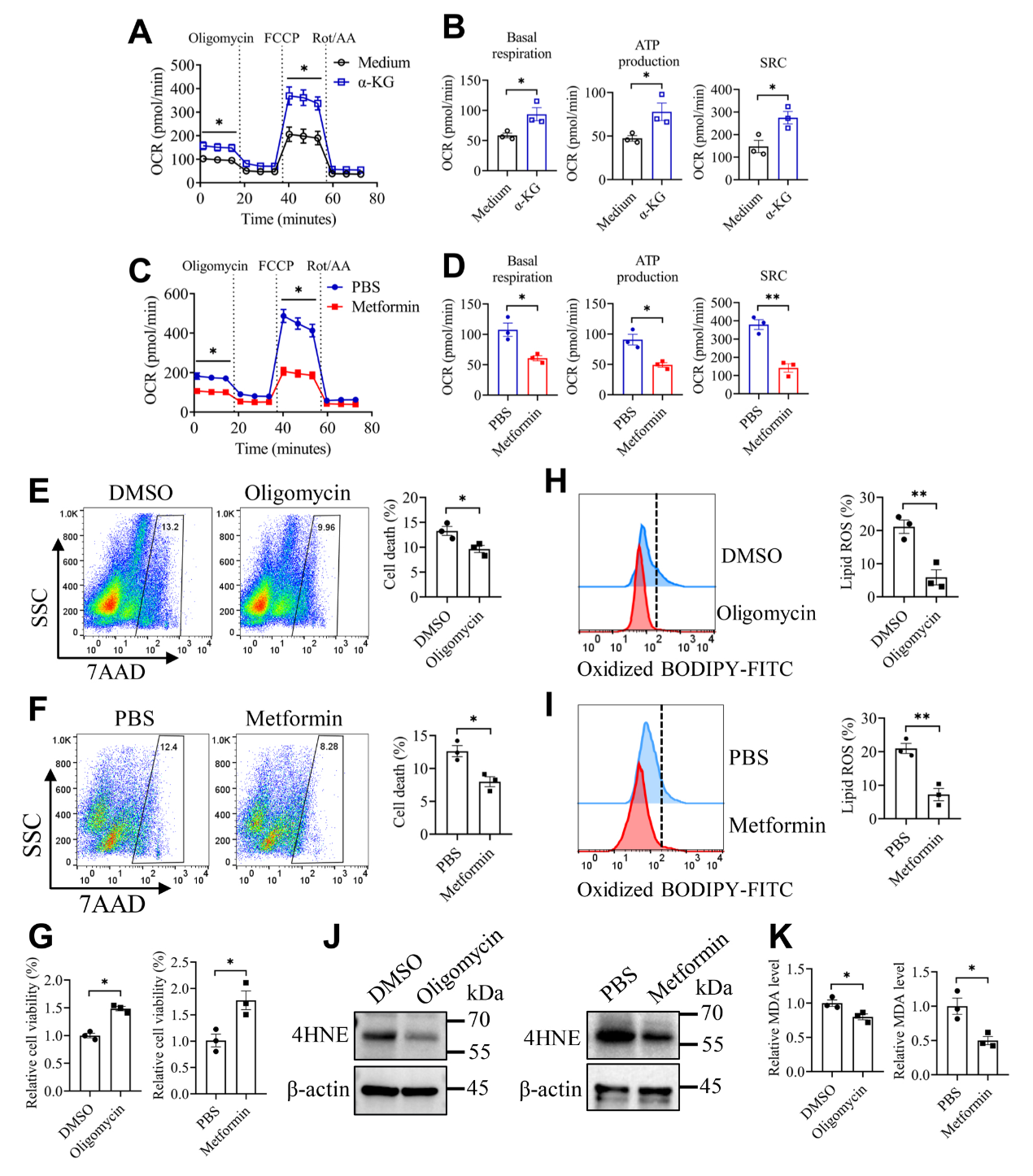


**Fig. S6. Ferroptosis of MDSCs promoted by** **CARD9 ablation is attributable to enhanced mitochondrial OXPHOS upon *C. tropicalis* stimulation.** **Related to Fig. 6. (A and B)** WT MDSCs were stimulated with *C. tropicalis* (MOI = 1) in combination with or without Dimethyl 2-oxoglutarate (α-KG) (8 mM) for 24 h. Oxygen consumption rate (OCR) of MDSCs was detected by Seahorse Cell Mito Stress Test **(A)**. Basal respiration, ATP production and spare respiratory capacity (SRC) were calculated based on Seahorse Cell Mito Stress Test **(B)**. **(C-K)** *Card9*^-/-^ MDSCs were stimulated with *C. tropicalis* (MOI = 1) in combination with or without oligomycin (5 μM) or metformin (1 mM) for 24 h. Oxygen consumption rate (OCR) of MDSCs was detected by Seahorse Cell Mito Stress Test **(C and D)**. **(E and F)** The percentage of cell death was determined using 7AAD. **(G)** The relative cell viability was detected by CCK-8 kit. **(H and I)** The Lipid ROS production was measured by BODIPY 581/591 C11 staining followed by flow cytometry. **(J)** The protein level of 4HNE was determined by western blot. **(K)** The relative MDA level was measured by MDA Assay Kit. Data with error bars are expressed as mean ± SEM, n = 3. Each panel shows at least three independent biological replicates from a typical experiment. Statistical analysis was determined by the unpaired Student’s t-test. ns (not significant), P > 0.05; *P < 0.05, **P < 0.01, ***P < 0.001.


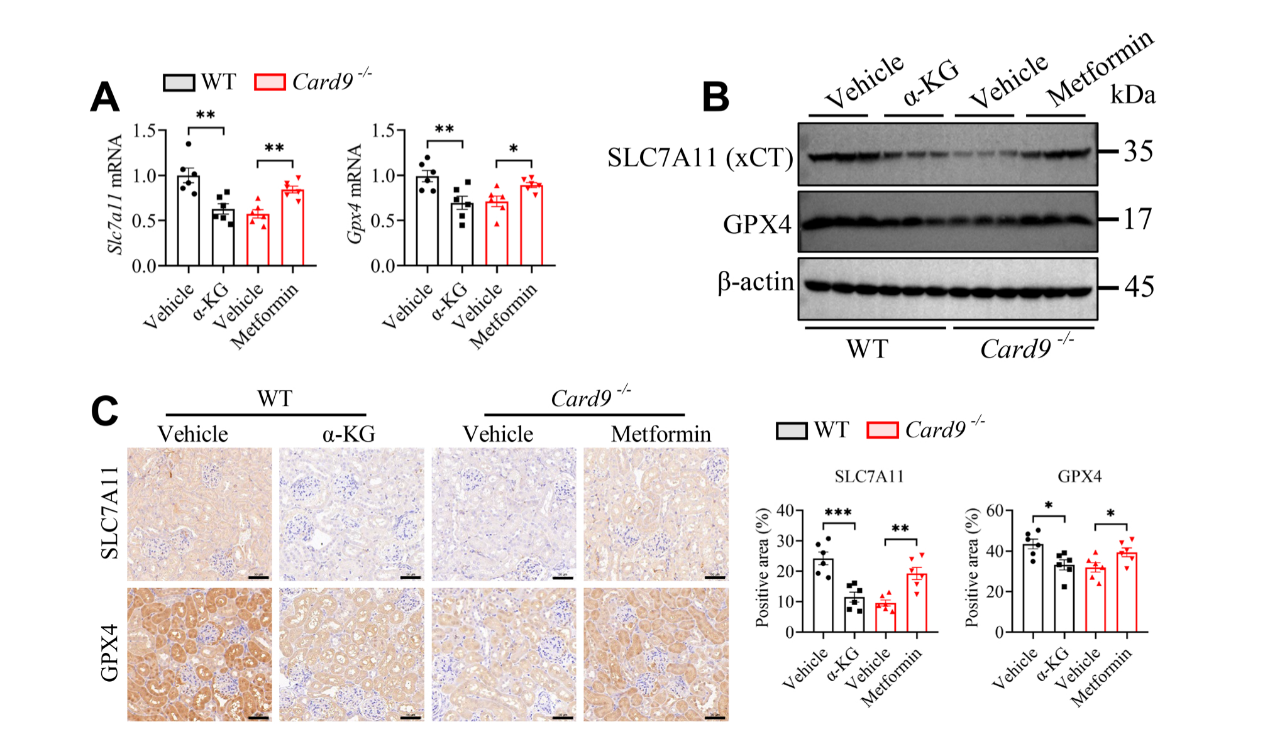


**Fig. S7. Renal injury and ferroptosis aggravated by CARD9 ablation are caused by OXPHOS during disseminated *C. tropicalis* infection. Related to Fig. 7.** **(A-C)** The expression of SLC7A11 and GPX4 in infected kidneys were determined by qPCR **(A)**, western blot **(B)** and IHC assay **(C)**. **(C)** Scale bar: 50 μM. The percentages of SLC7A11-positive and GPX4-positive were calculated. Data with error bars are expressed as mean ± SEM, n = 6. Each panel shows at least six independent biological replicates from a typical experiment. Statistical analysis was determined by one-way ANOVA with Bonferroni's multiple comparisons test for post-hoc test. ns (not significant), P > 0.05; *P < 0.05, **P < 0.01, ***P < 0.001.


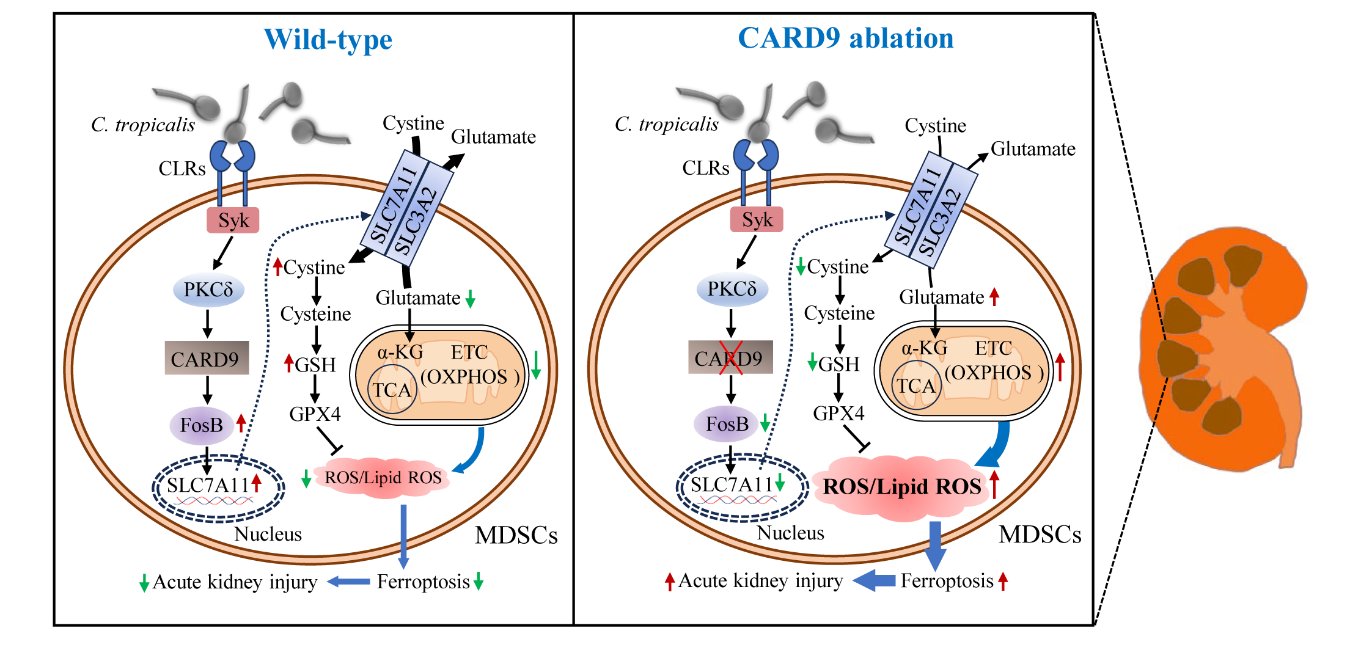


**Fig. S8. Graphical abstract demonstrates how CARD9 deficiency-induced ferroptosis negatively modulates antifungal immunity to exacerbate acute kidney injury**.

**Supplementary Tables**

**Table. S1 Primer sequences for qRT-PCR**

| **Gene (Mouse)** | **Forward Primer (5’→3’)** | **Reverse Primer (5’→3’)** |
| --- | --- | --- |
| *β-actin* | ATTGTTACCAACTGGGACGACATG | CTTCATGAGGTAGTCTGTCAGGTC |
| *Slc7a11* | TGGCGGTGACCTTCTCTGA | ACAAAGATCGGGACTGCTAATGA |
| *Slc3a2* | TGATGAATGCACCCTTGTACTTG | GCTCCCCAGTGAAAGTGGA |
| *Gpx4* | CCTCCCCAGTACTGCAACAG | GGCTGAGAATTCGTGCATGG |
| *Acsl4* | CTCACCATTATATTGCTGCCTGT | TCTCTTTGCCATAGCGTTTTTCT |
| *Nrf2* | TCTTGGAGTAAGTCGAGAAGTGT | GTTGAAACTGAGCGAAAAAGGC |
| *Ho1* | GCTGGTGATGGCTTCCTTGT | TTGTTGCGCTCTATCTCCTCTTC |
| *Atf3* | GGAGTCAGTTACCGTCAA | TGTATATCAAATGCTGTTTCTCA |
| *Gls2* | CAGAGGGACAGGAGCGTATC | TTCTTTCGGAATGCCTGAGTC |
| *Fth1* | CAAGTGCGCCAGAACTACCA | GCCACATCATCTCGGTCAAAA |
| *Tf* | GCTGTCCCTGACAAAACGGT | CGGAAGGACGGTCTTCATGTG |
| *Fosb* | TTTTCCCGGAGACTACGACTC | GTGATTGCGGTGACCGTTG |
| *Glud1* | CCCTGCAAGGGAGGTATCC | GACCACAGCGCACTTGTATG |
| *Ndufb8* | TGTTGCCGGGGTCATATCCTA | AGCATCGGGTAGTCGCCATA |
| *Sdhb* | AATTTGCCATTTACCGATGGGA | AGCATCCAACACCATAGGTCC |
| *Uqcrc2* | AAAGTTGCCCCGAAGGTTAAA | GAGCATAGTTTTCCAGAGAAGCA |
| *Cox* *Ⅳ* | ATTGGCAAGAGAGCCATTTCTAC | CACGCCGATCAGCGTAAGT |
| *Atp5a1* | TCTCCATGCCTCTAACACTCG | CCAGGTCAACAGACGTGTCAG |
| *Tom20* | GCCCTCTTCATCGGGTACTG | ACCAAGCTGTATCTCTTCAAGGA |
| *Tim23* | GAAGGTGGCGGAAGAAGTAGC | GGGGGTTCATACCAGTCAGC |

**Table. S2 siRNA Sequence**

| Gene | siRNA | Sequence |
| --- | --- | --- |
| Mouse *Fosb* | siRNA | CCAGGAACCAGCTACTCAA |
| Mouse *Slc7a11* | siRNA#1 | CTGGAGTTATACAGCTAAT |
|  | siRNA#2 | CGATACAAACGCCCAGATA |
