# Supplementary material for: Mitochondria-mediated ferroptosis induced by CARD9 ablation prevents MDSCs-dependent antifungal immunity
Source: Cell Commun Signal. 2024 Apr 2;22:210. doi: 10.1186/s12964-024-01581-2 (PMC10986078; doi:10.1186/s12964-024-01581-2)

Fig. 1E

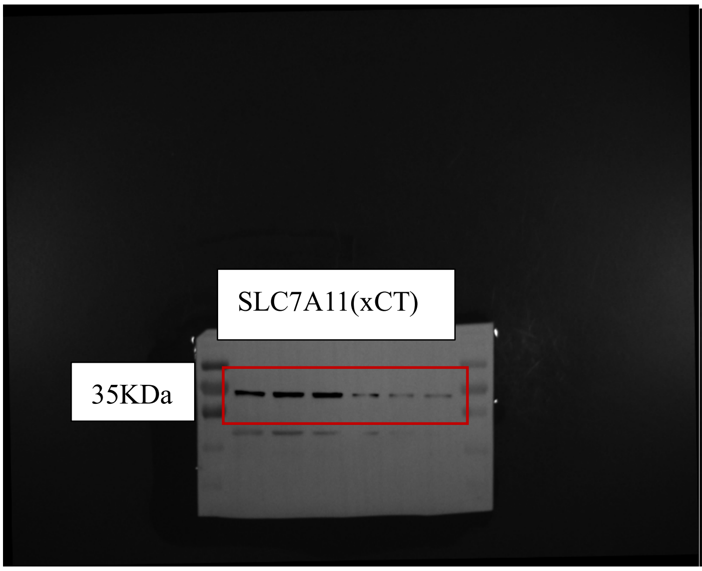
SLC7A11(xCT)
35KDa

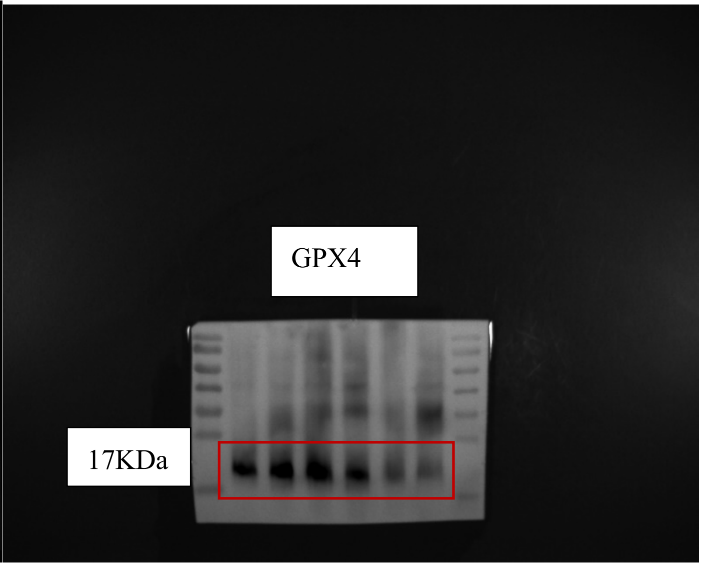
GPX4
17KDa

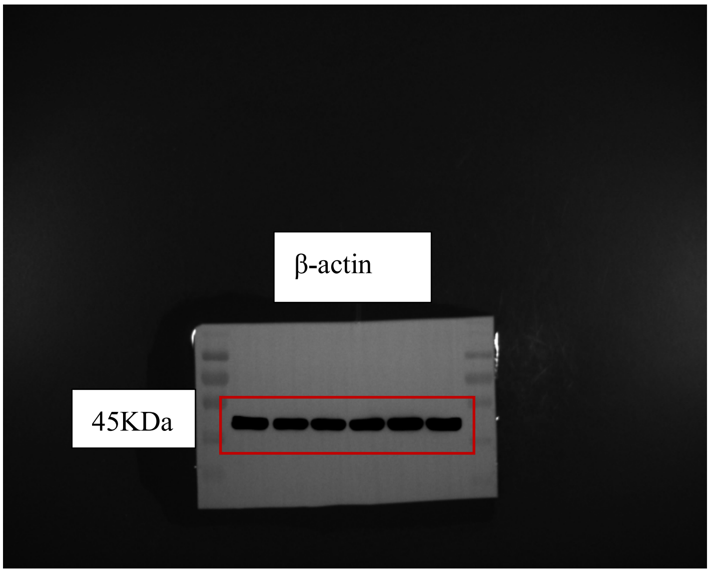
β-actin
45KDa

Fig. 2C

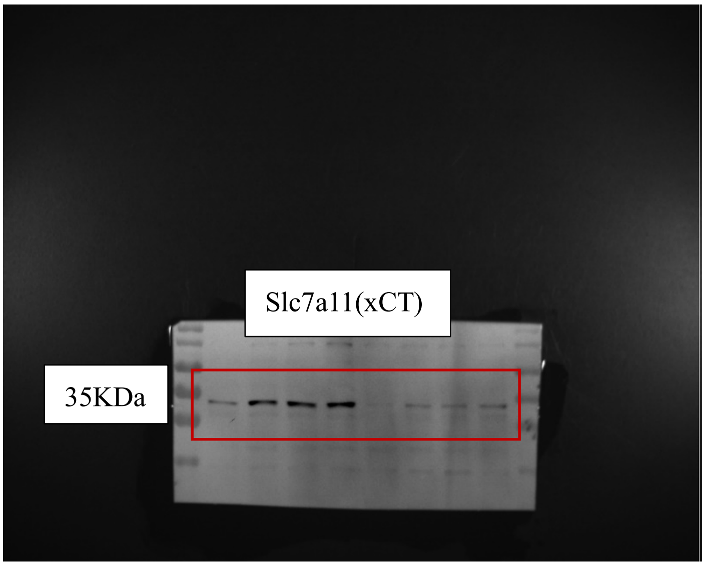
Slc7a11(xCT)
35KDa

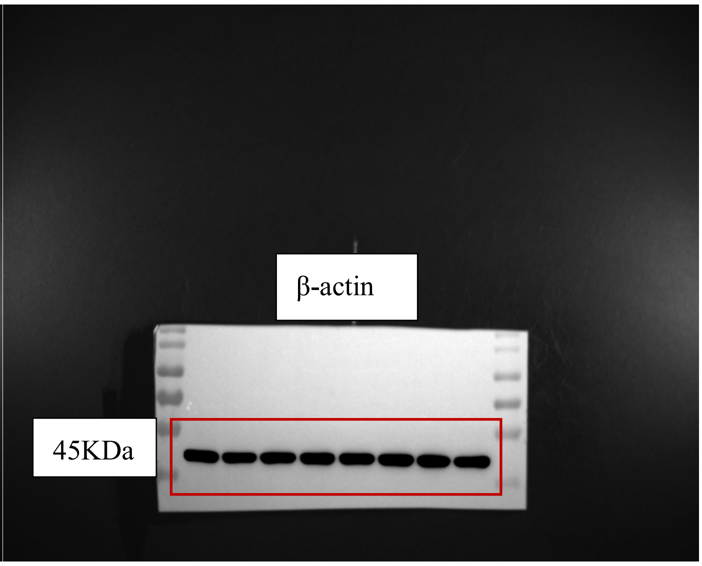
β-actin
45KDa

Fig. 2D

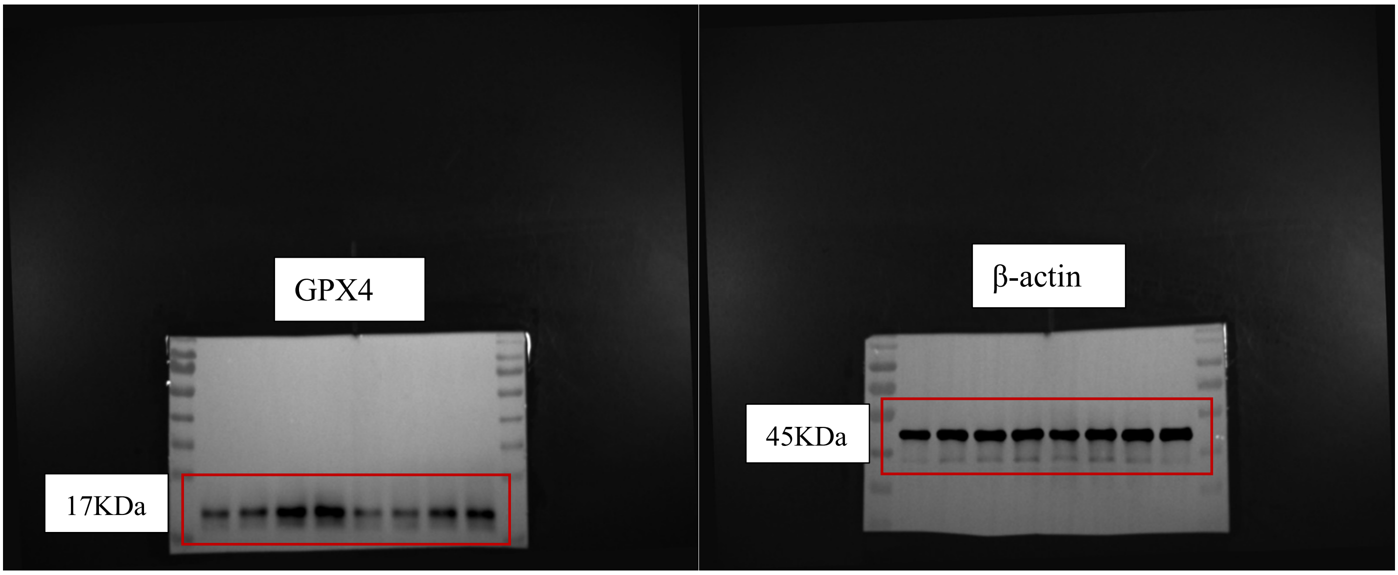

Fig. 2K

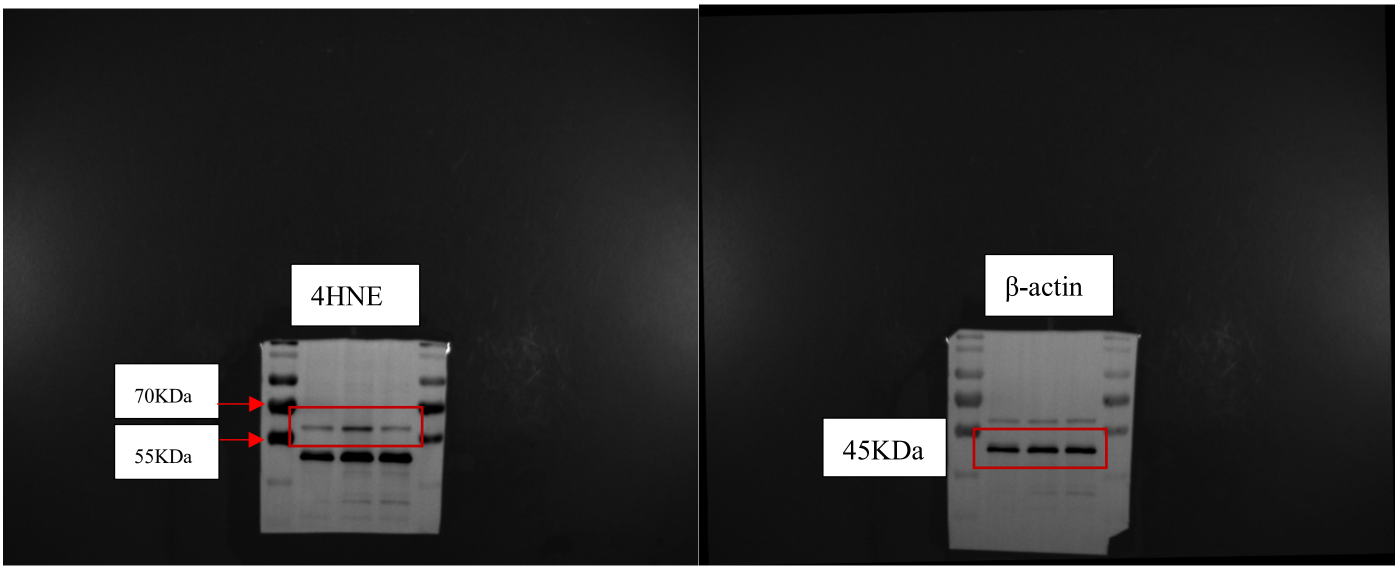

Fig. 3D

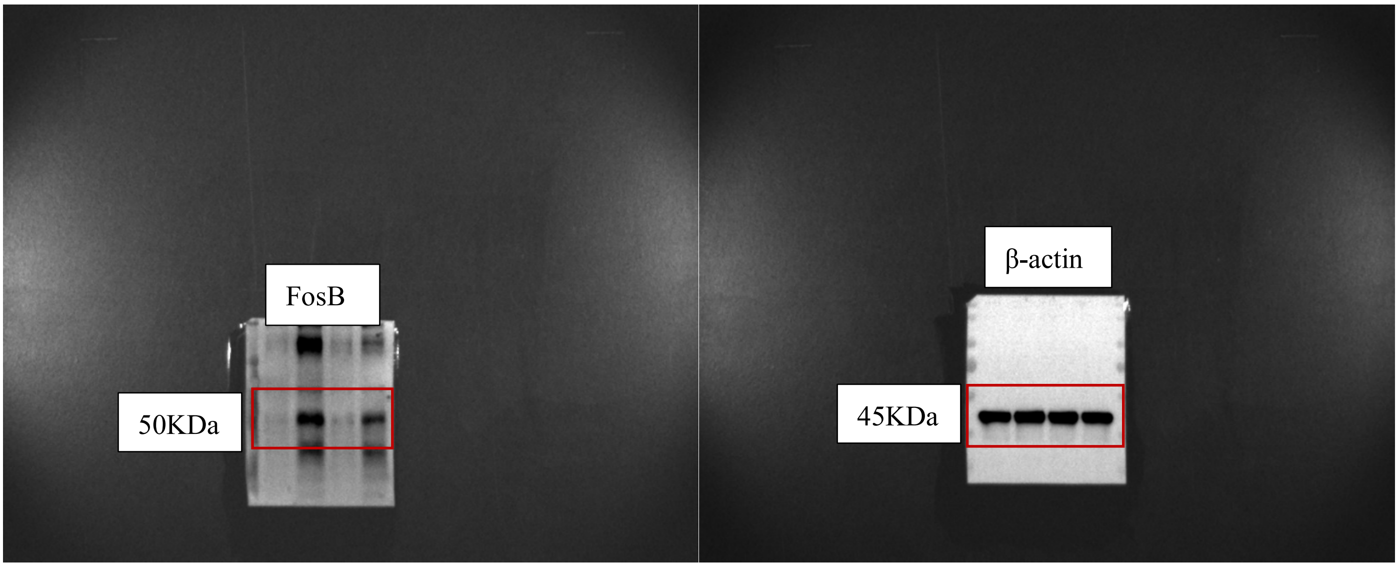

Fig. 3G

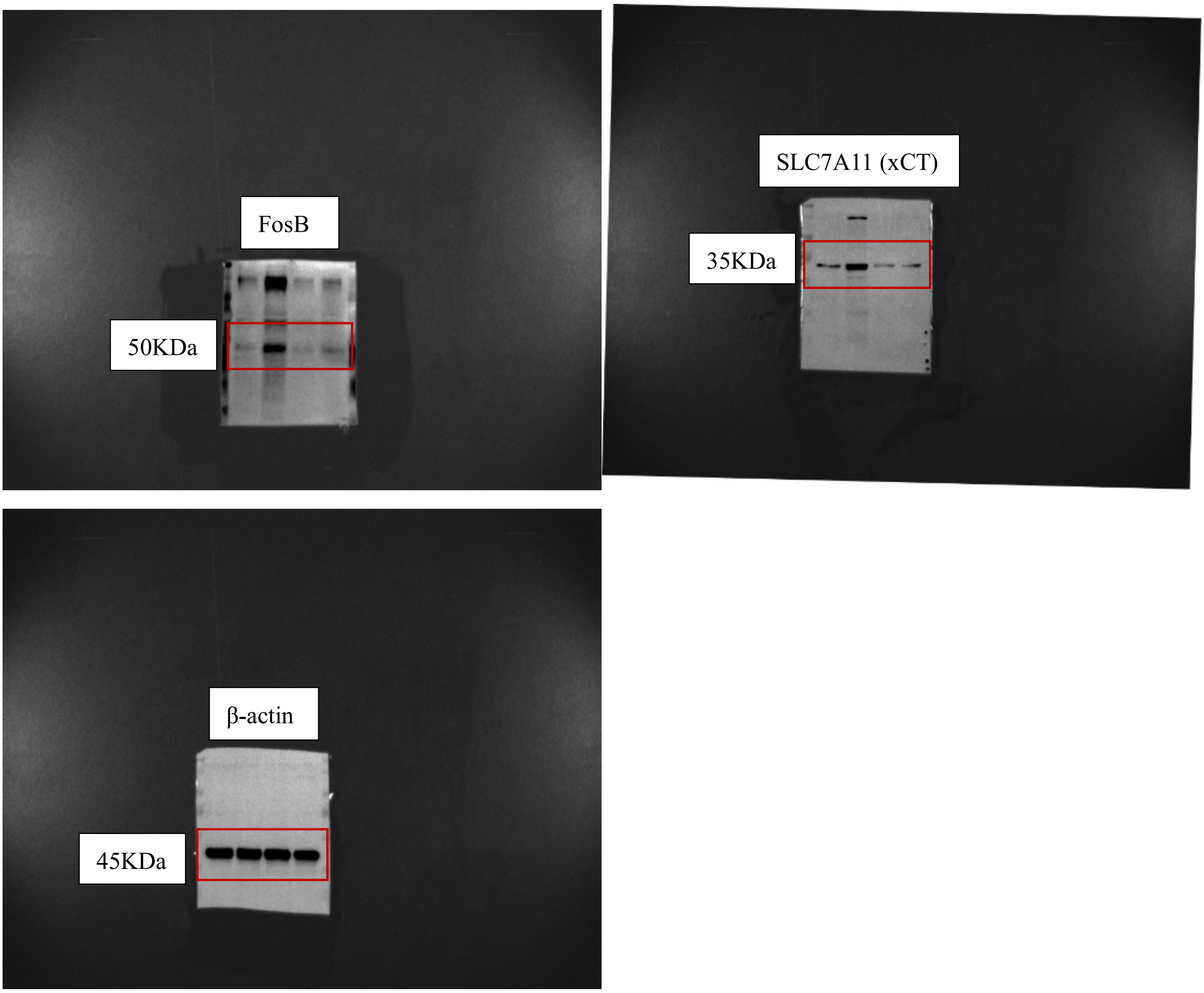
FosB
50KDa
SLC7A11 (xCT)
35KDa
β-actin
45KDa

Fig. 4D

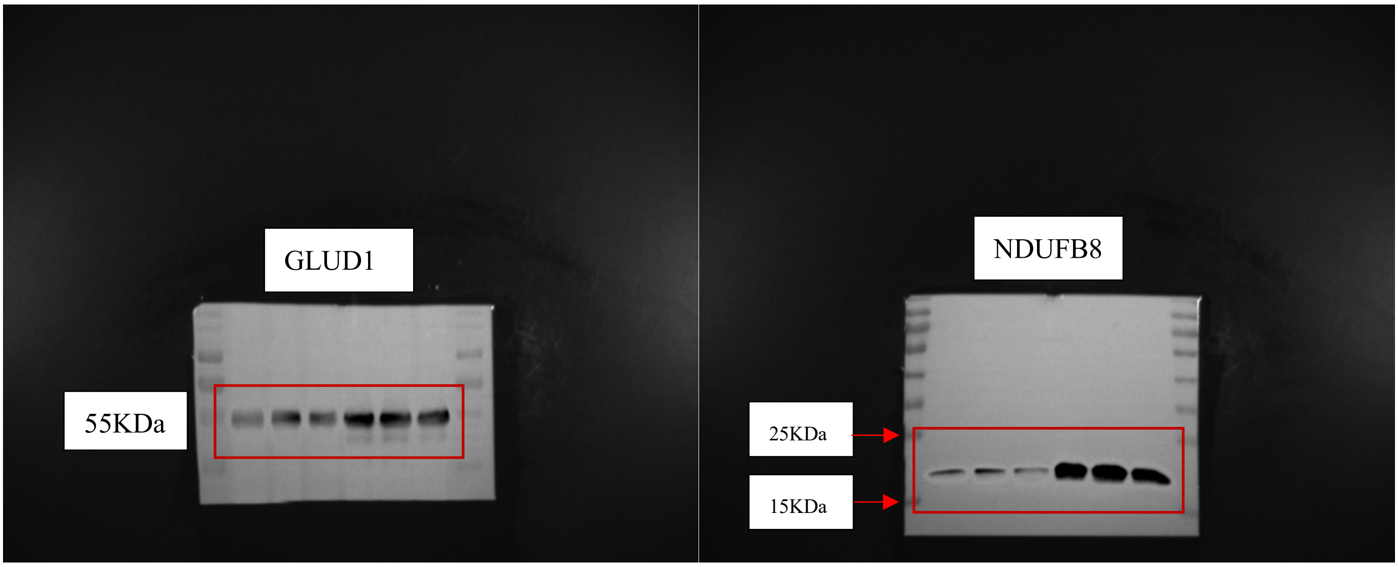
GLUD1
55KDa
NDUFB8
25KDa
15KDa

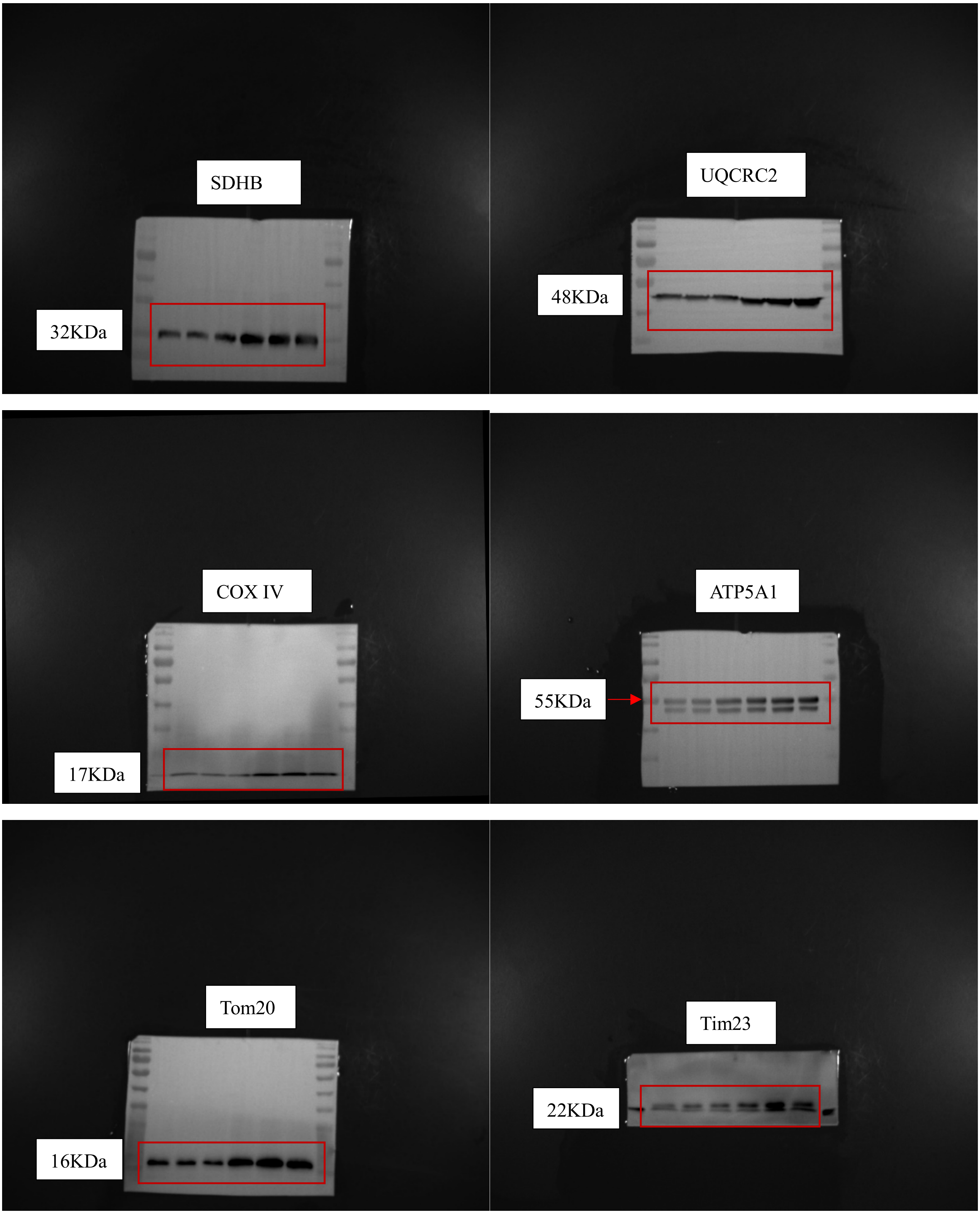
SDHB
32KDa
UQCRC2
48KDa
COX IV
17KDa
ATP5A1
55KDa
Tom20
16KDa
Tim23
22KDa

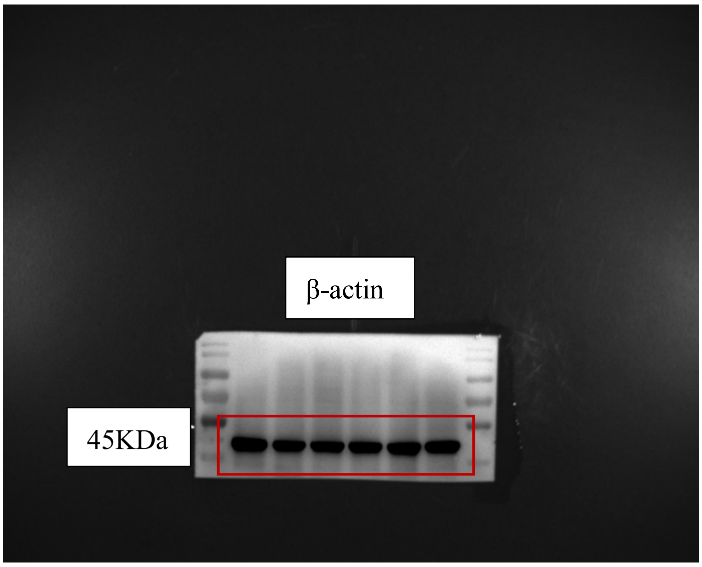
β-actin
45KDa

Fig. 4K

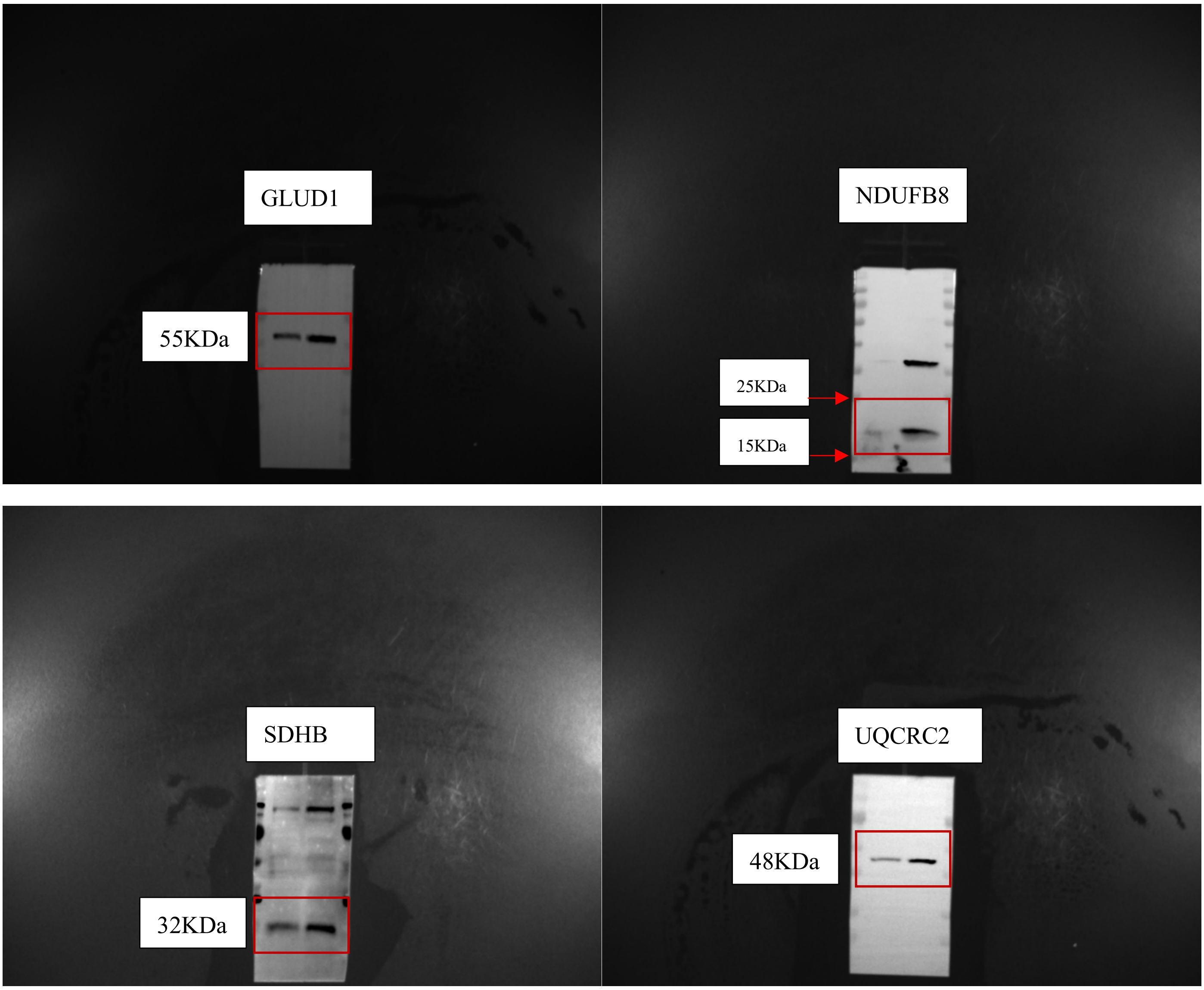
GLUD1
55KDa
NDUFB8
25KDa
15KDa
SDHB
32KDa
UQCRC2
48KDa

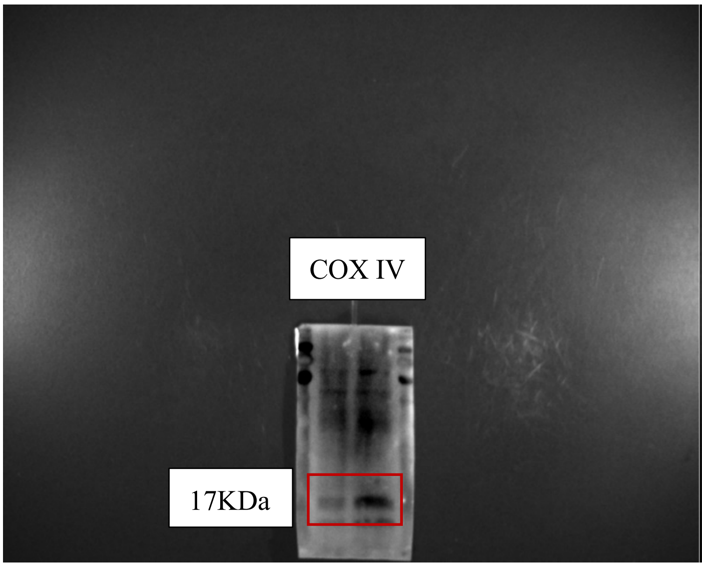
COX IV
17KDa

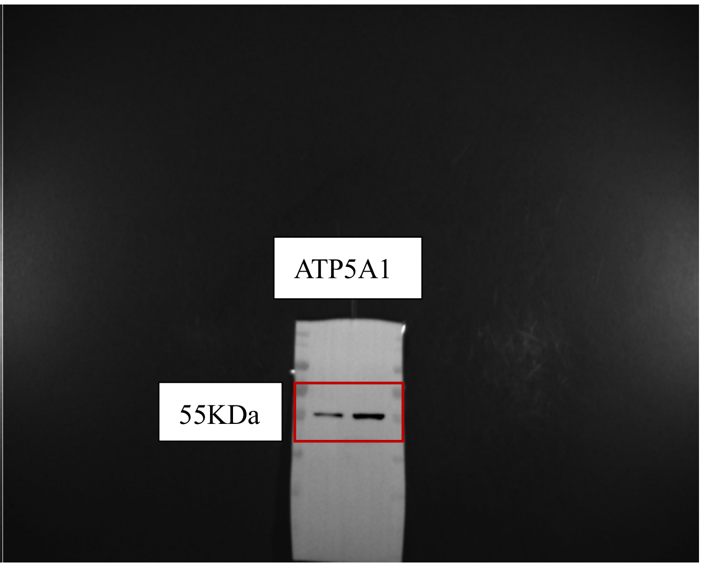
ATP5A1
55KDa

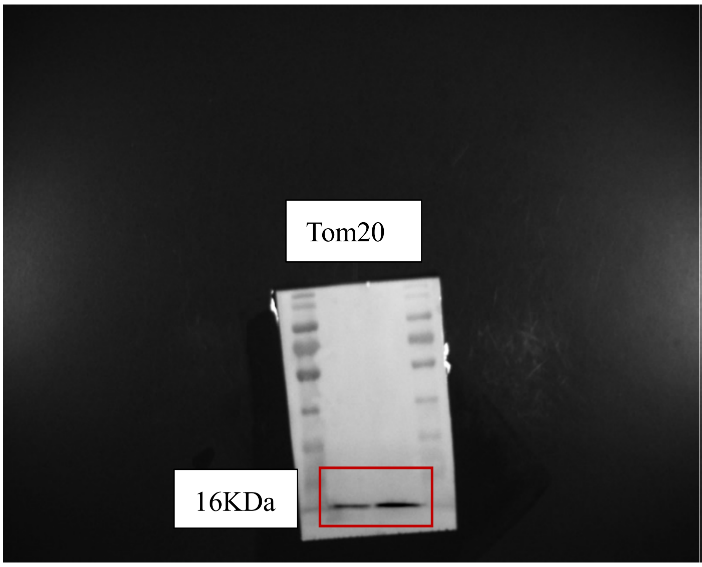
Tom20
16KDa

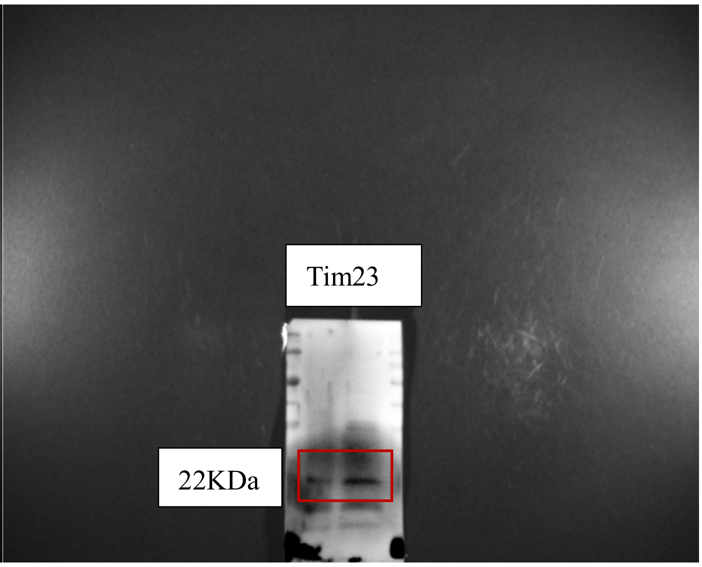
Tim23
22KDa

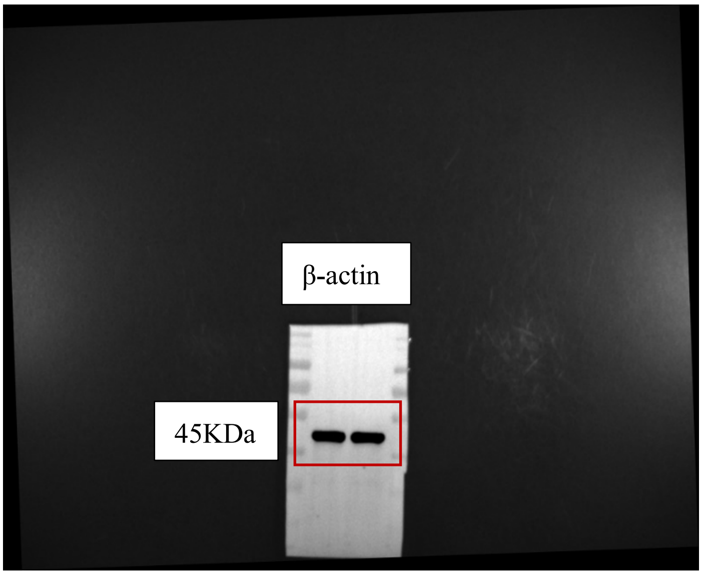
β-actin
45KDa

Fig. 5K

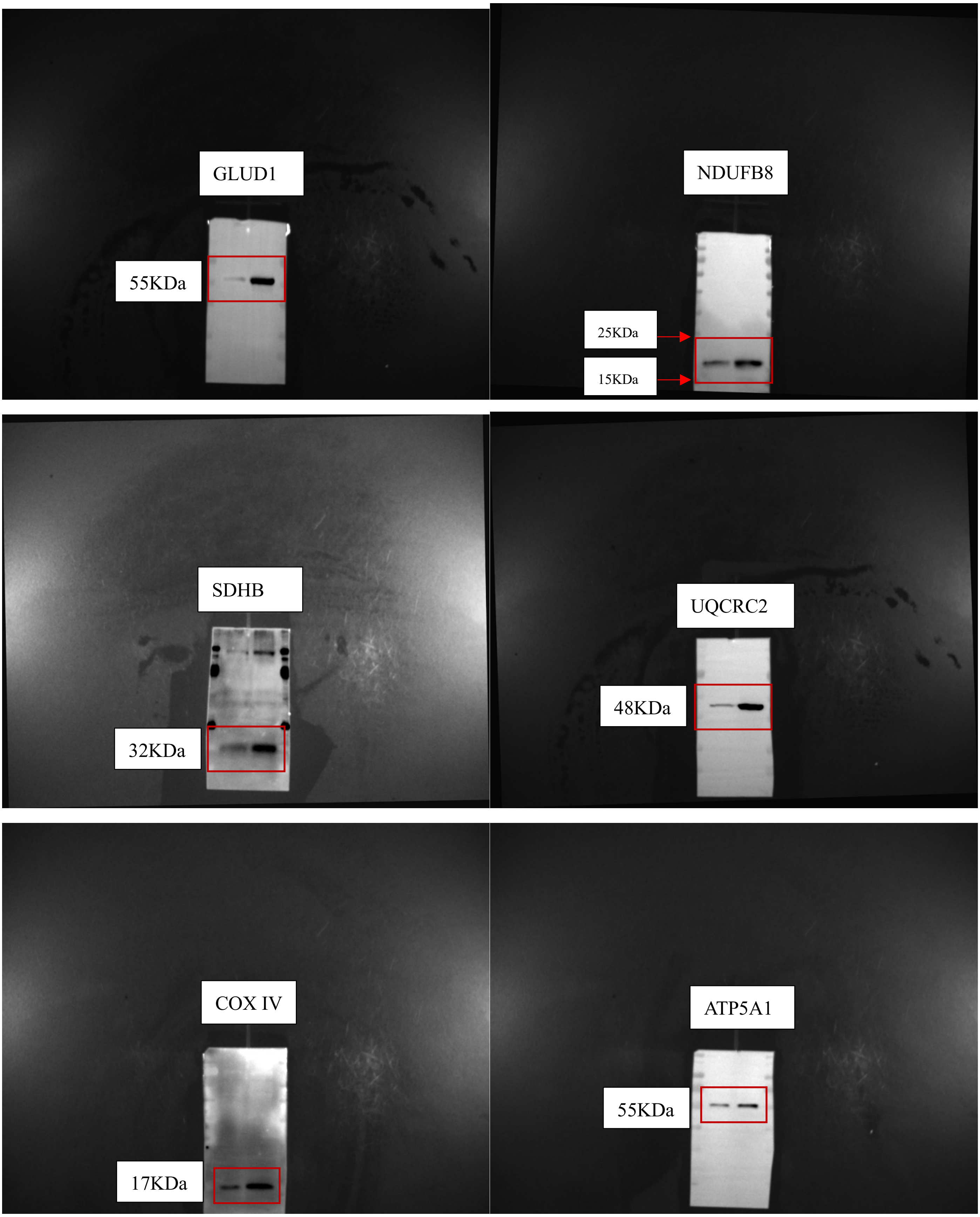
GLUD1
55KDa
NDUFB8
25KDa
15KDa
SDHB
32KDa
UQCRC2
48KDa
COX IV
17KDa
ATP5A1
55KDa

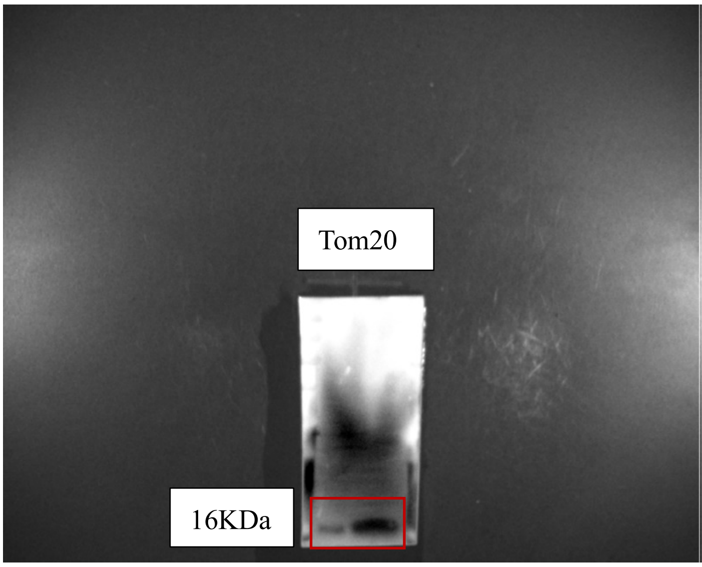
Tom20
16KDa

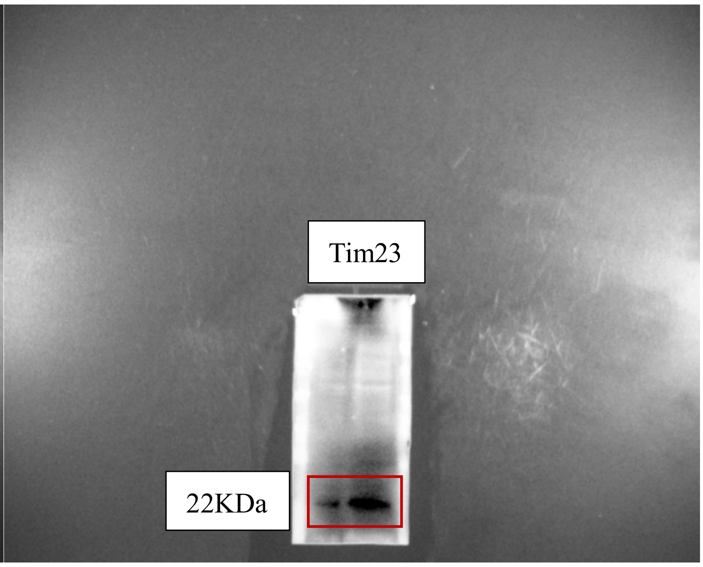
Tim23
22KDa

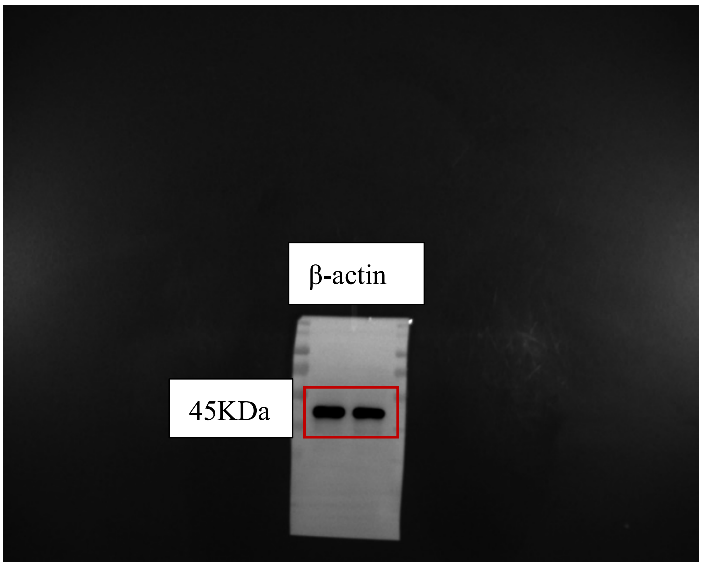
β-actin
45KDa

Fig. 5L

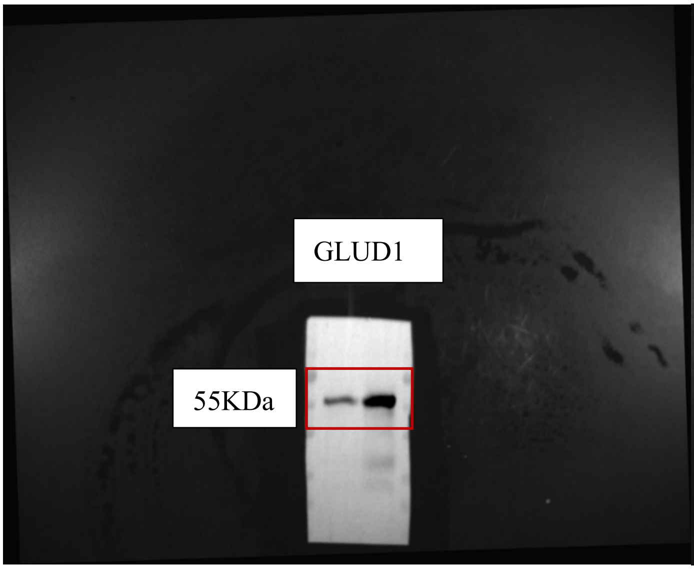
GLUD1
55KDa

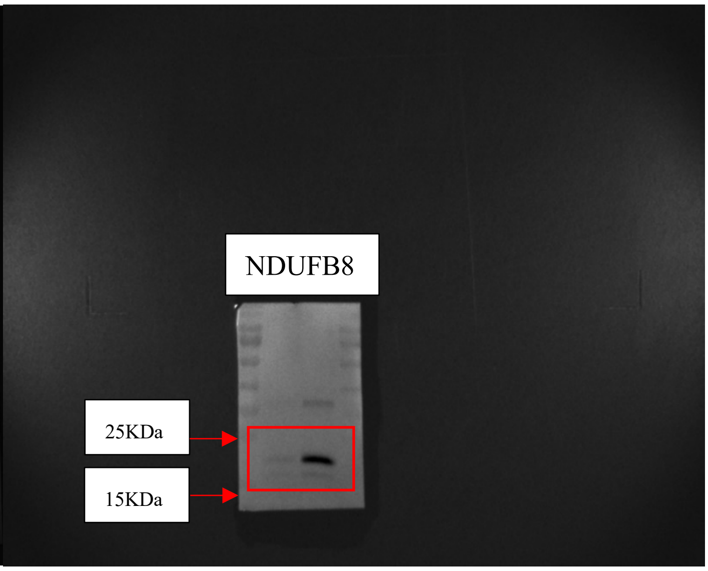
NDUFB8
25KDa
15KDa

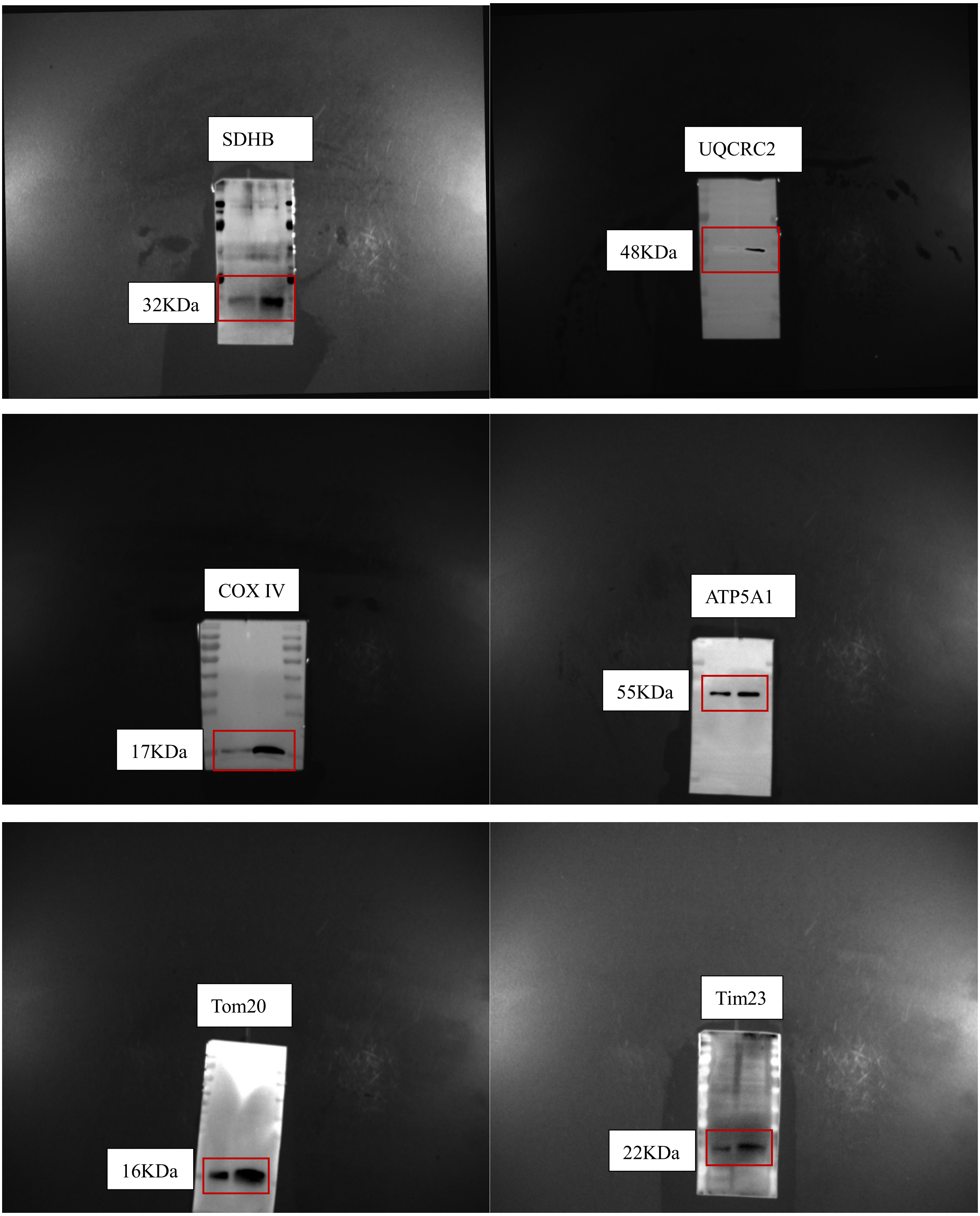
SDHB
32KDa
UQCRC2
48KDa
COX IV
17KDa
ATP5A1
55KDa
Tom20
16KDa
Tim23
22KDa

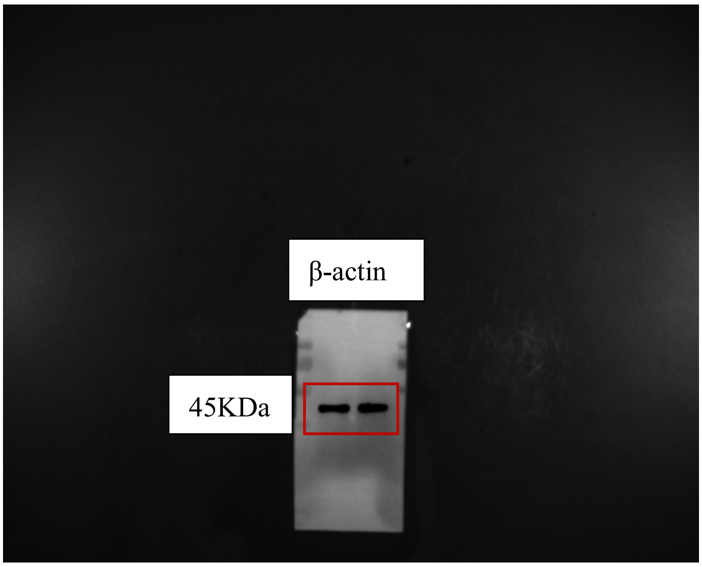
β-actin
45KDa

Fig. 6F

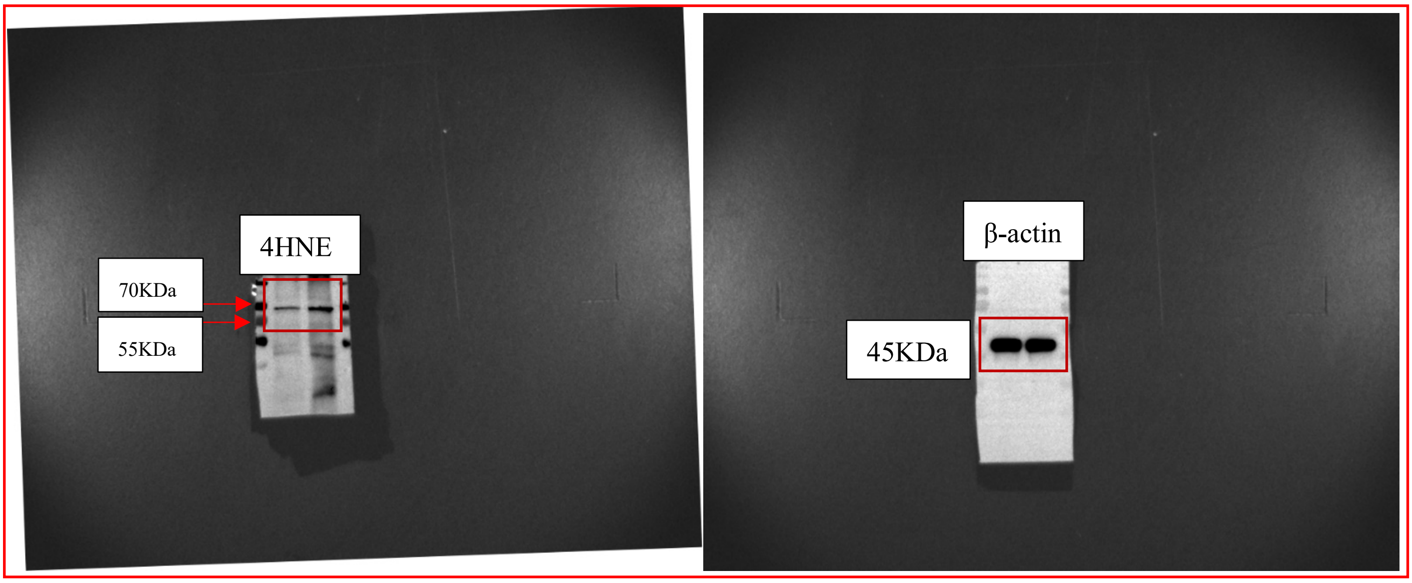
4HNE
70KDa
55KDa
β-actin
45KDa

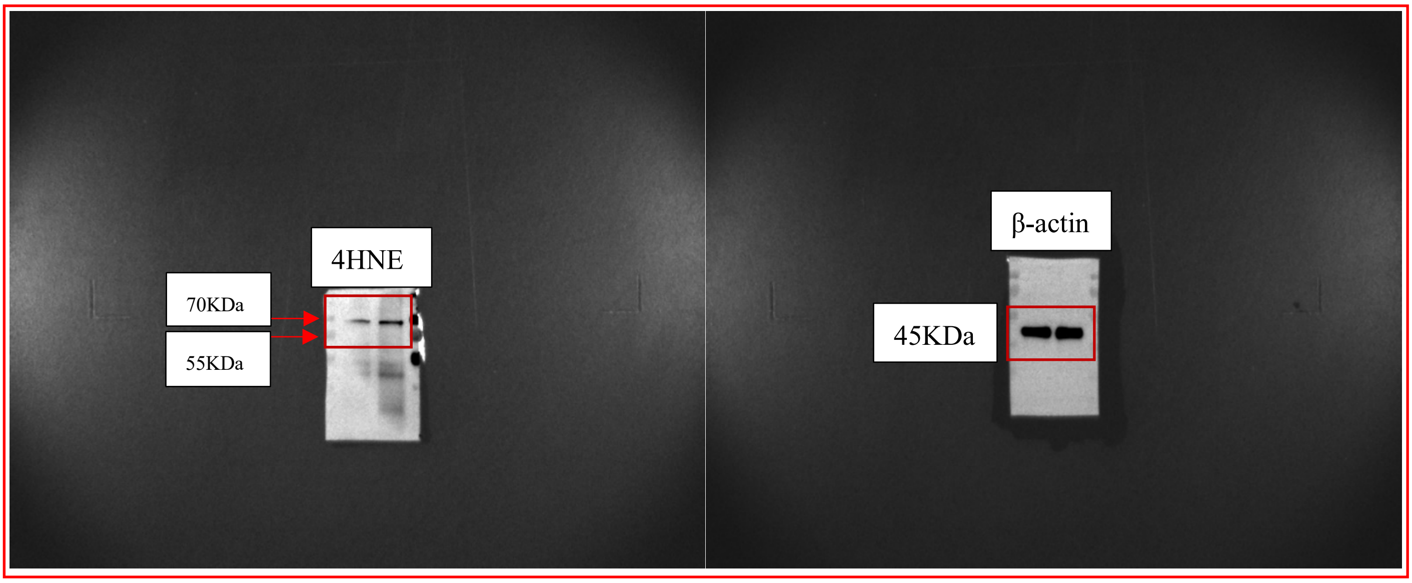
4HNE
70KDa
55KDa
β-actin
45KDa

Fig. S2D

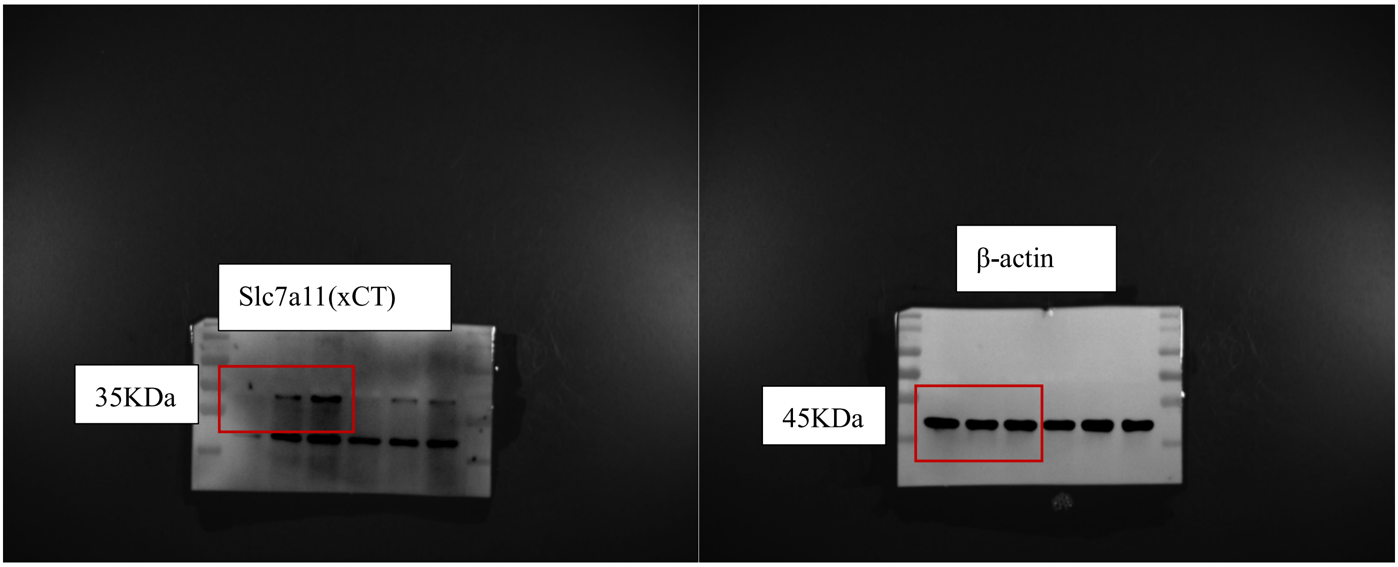

Fig. S3B

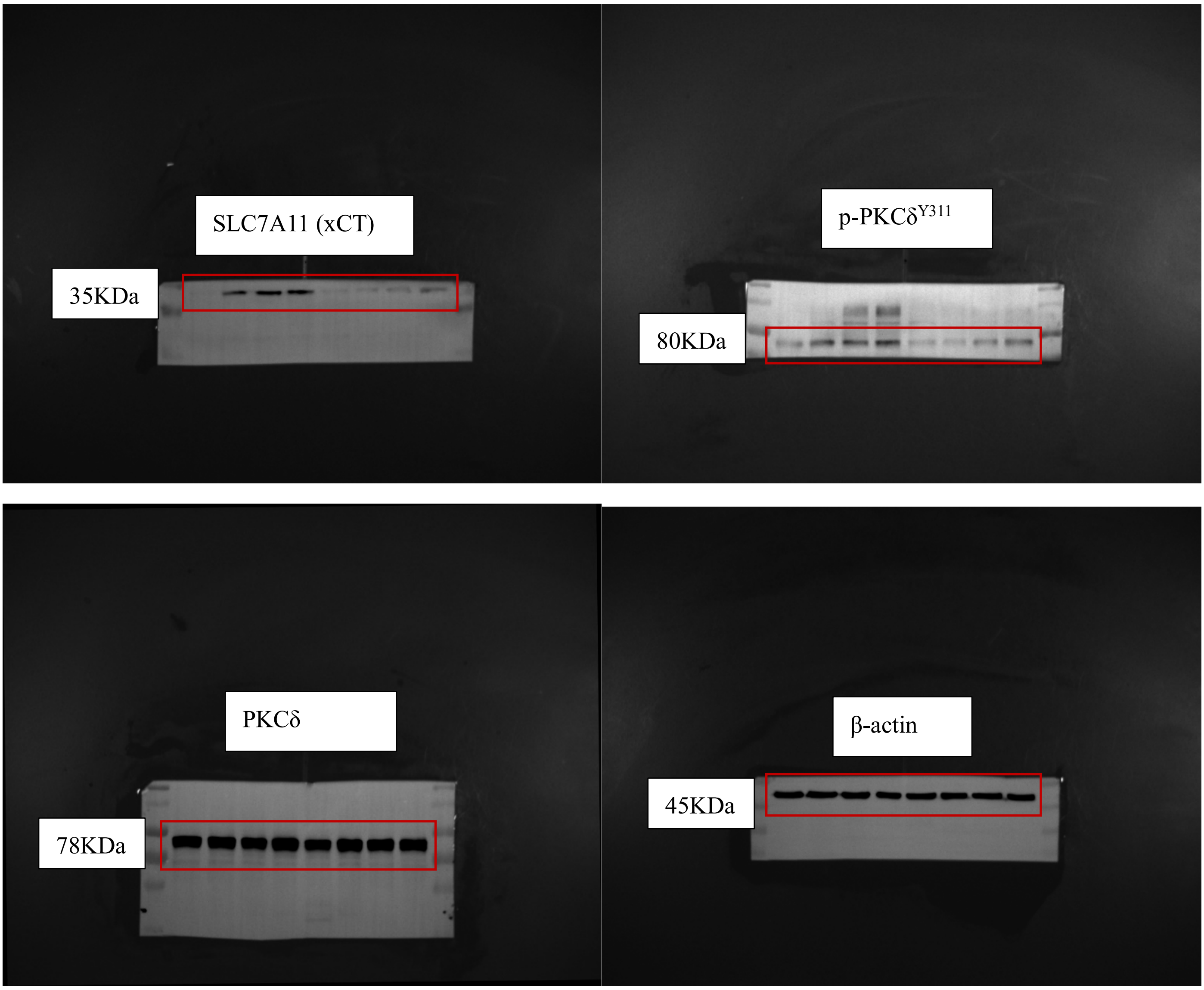

Fig. S3C

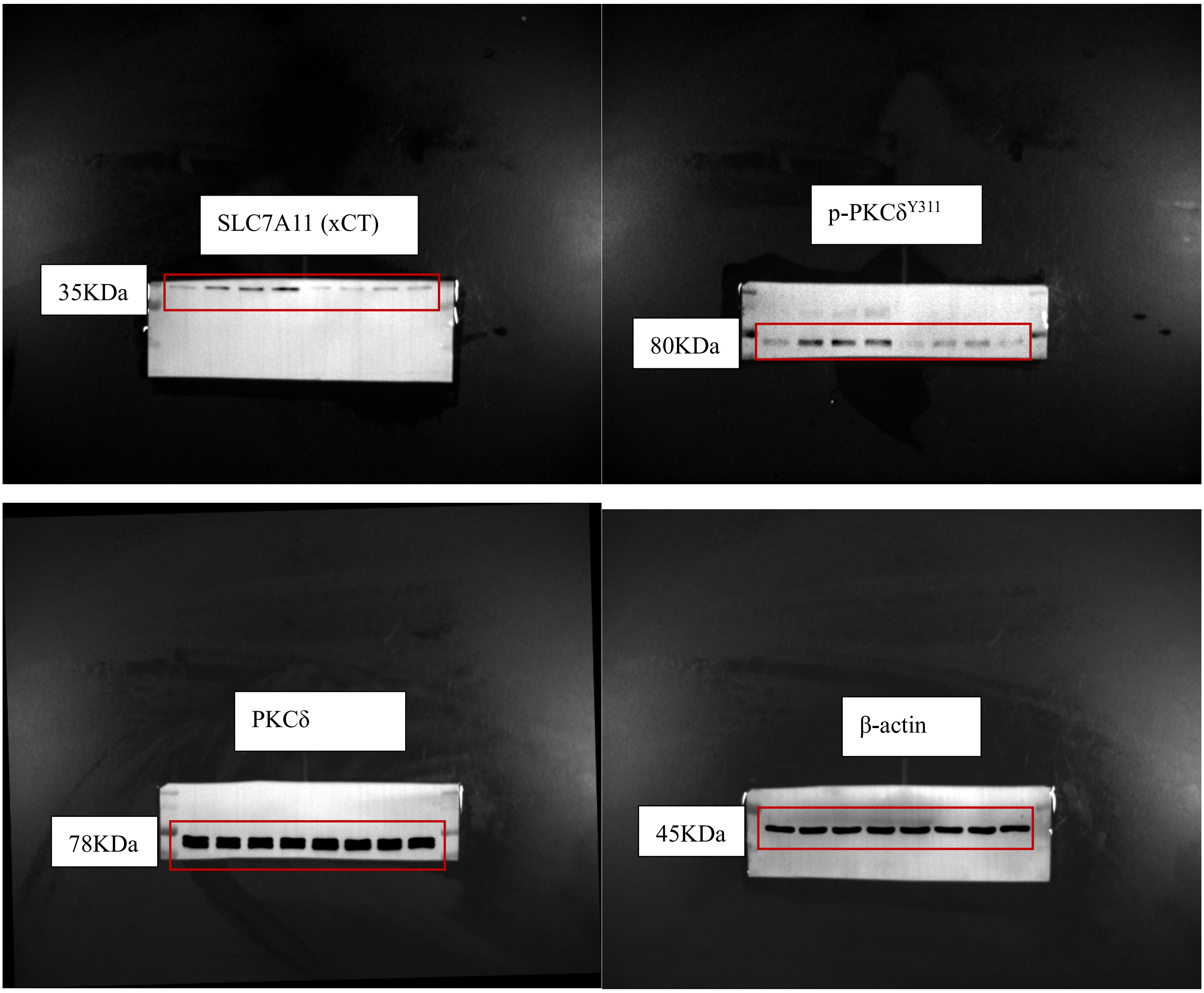

Fig. S3D

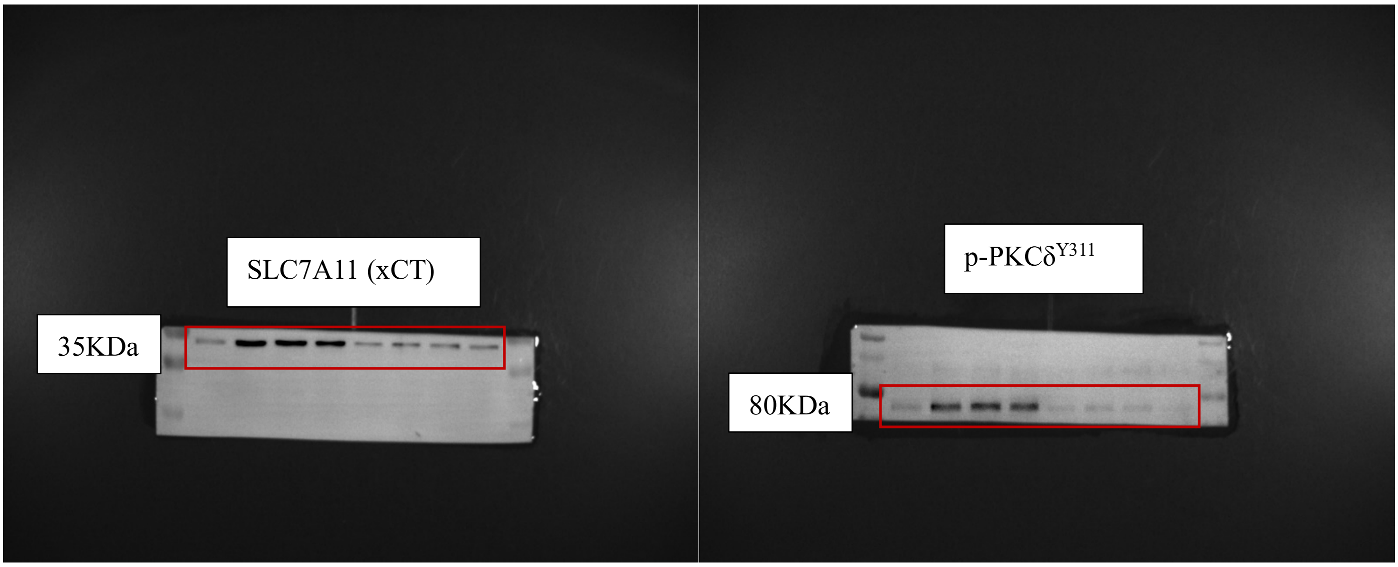

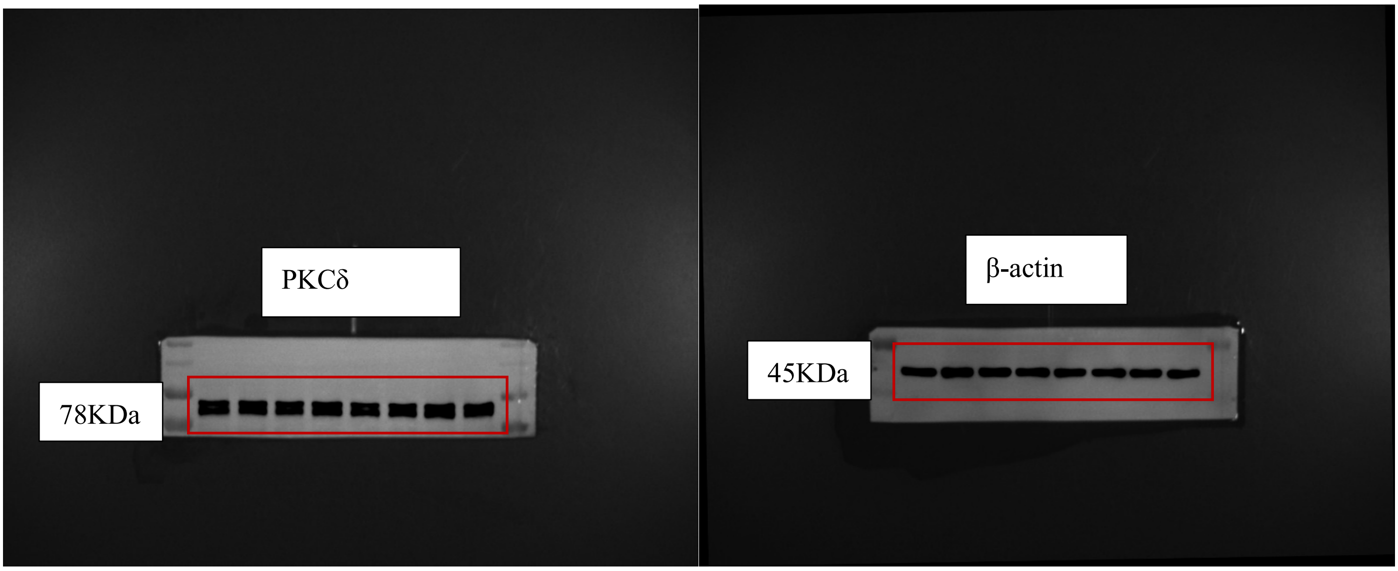
PKCδ
78KDa
β-actin
45KDa

Fig. S3E

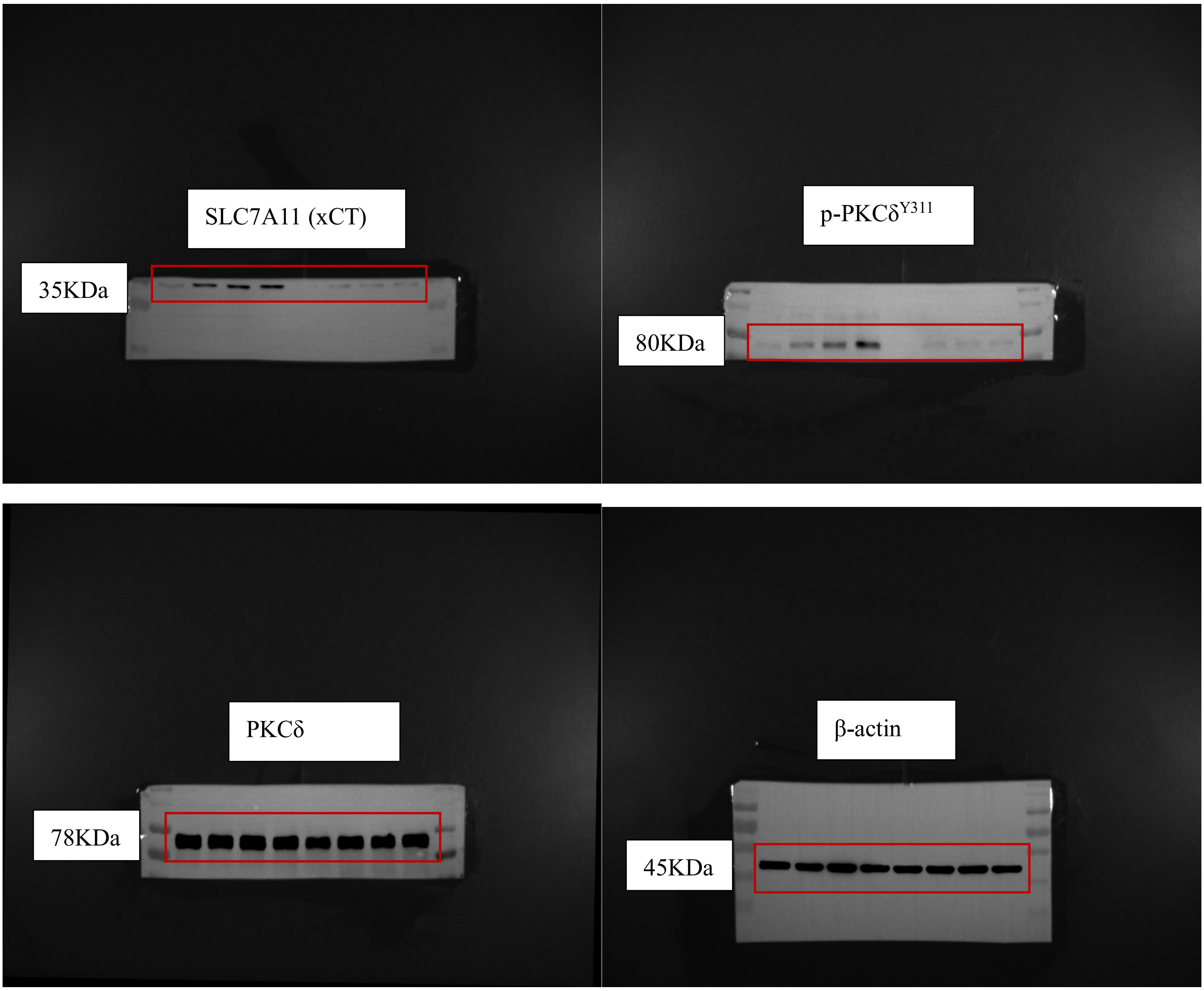
SLC7A11 (xCT)
35KDa
p-PKCδ Y311
80KDa
PKCδ
78KDa
β-actin
45KDa

Fig. S3F

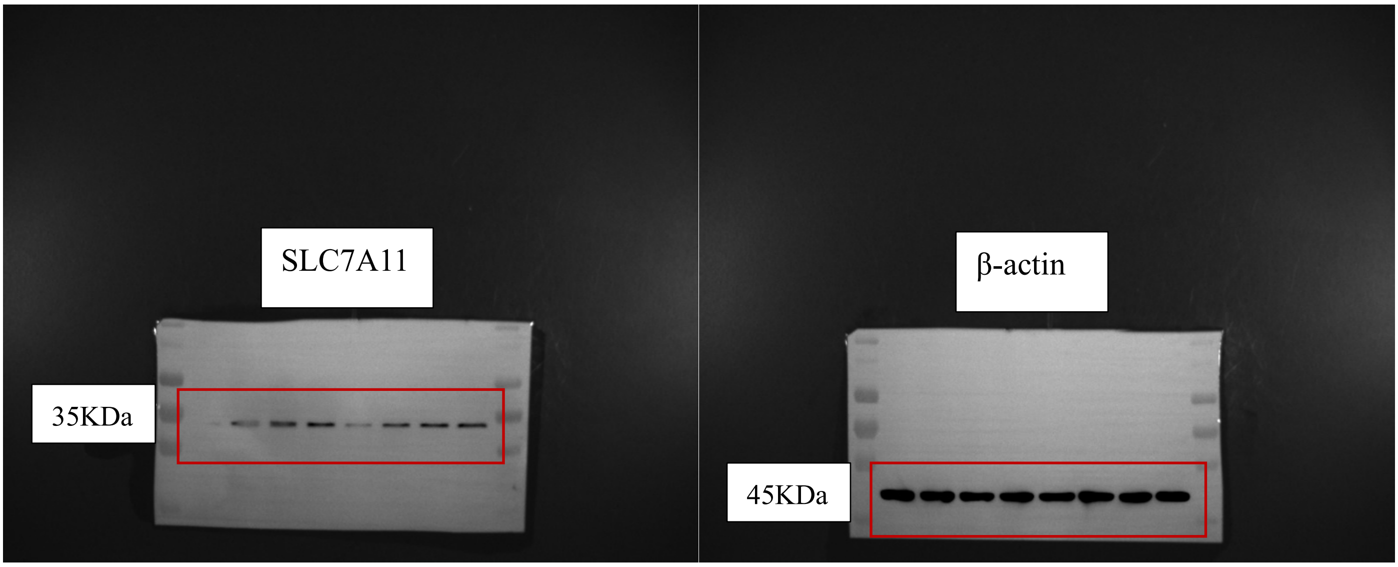

Fig. S4B

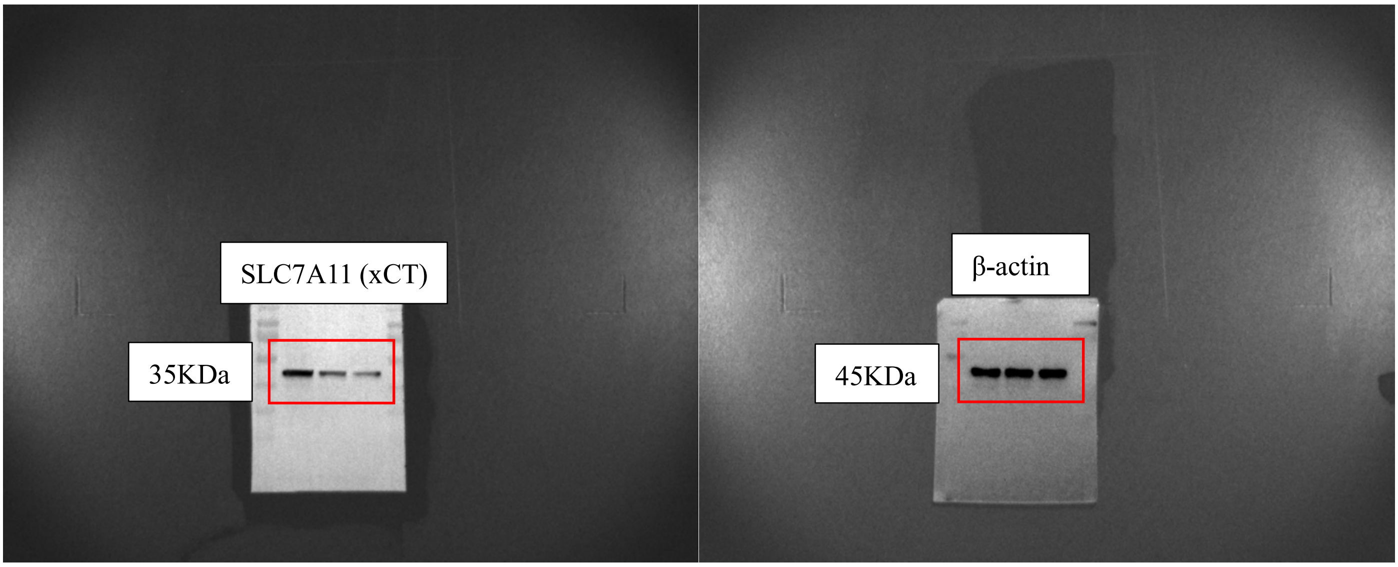

Fig. S4H

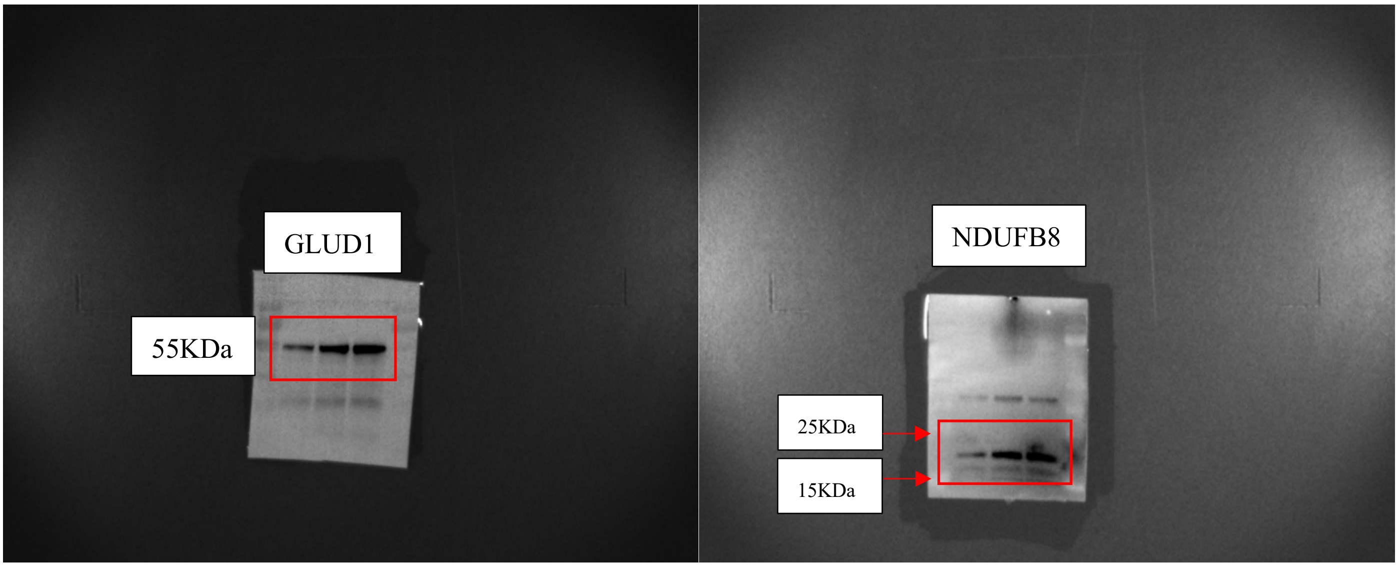

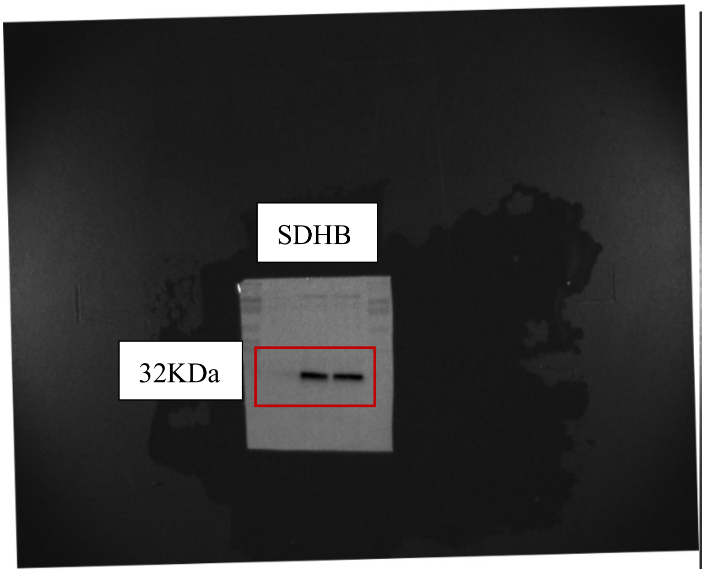
SDHB
32KDa

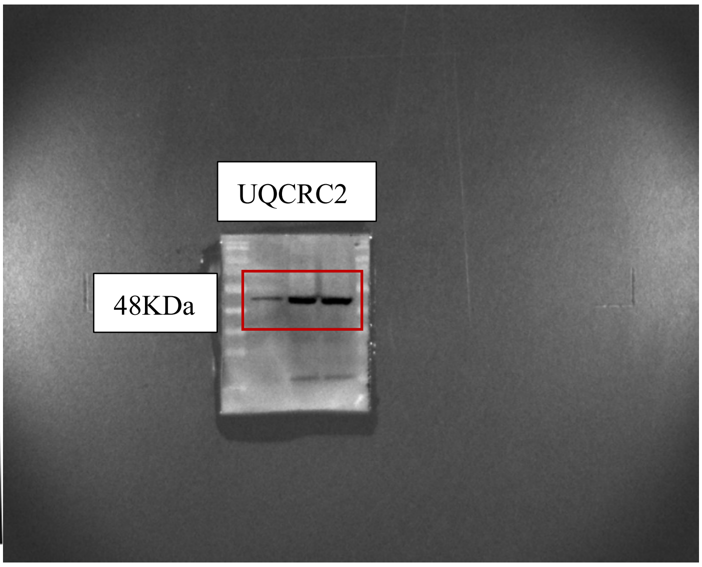
UQCRC2
48KDa

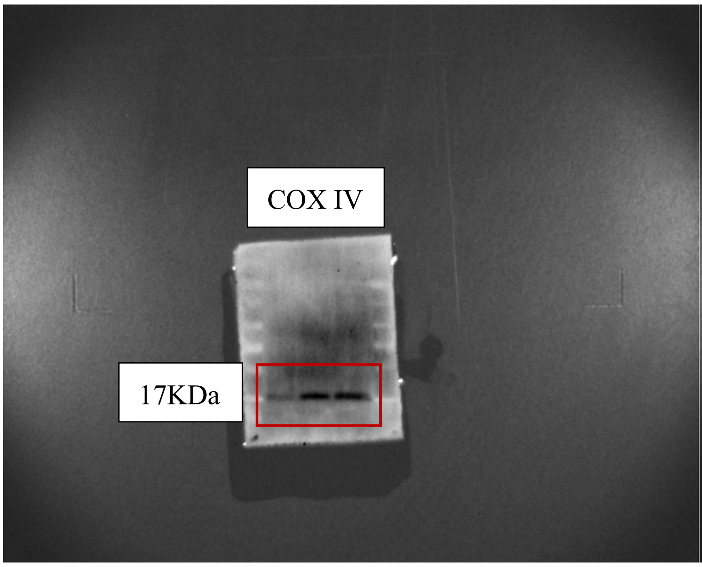
COX IV
17KDa

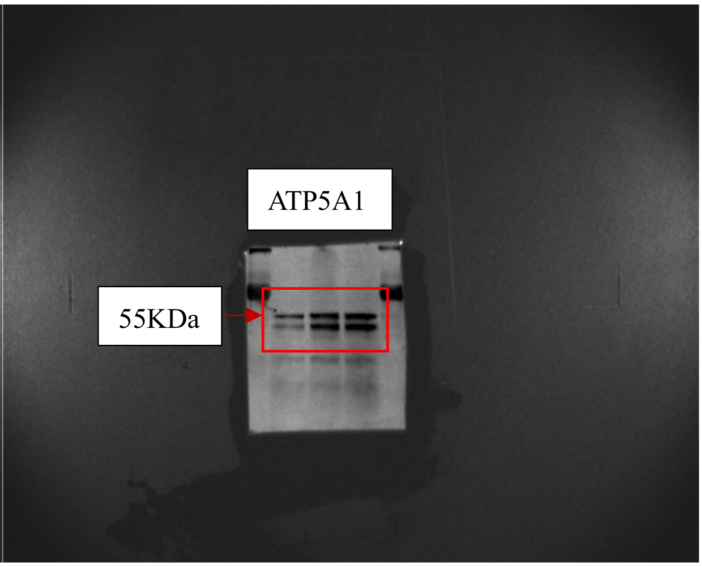
ATP5A1
55KDa

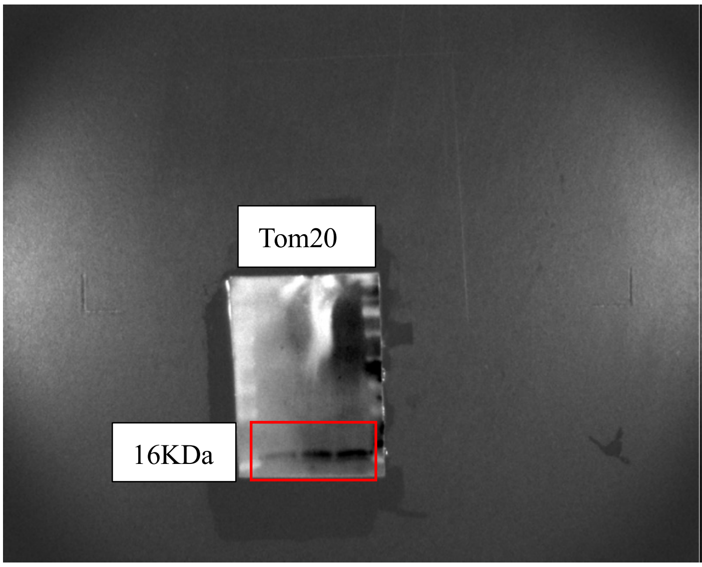
Tom20
16KDa

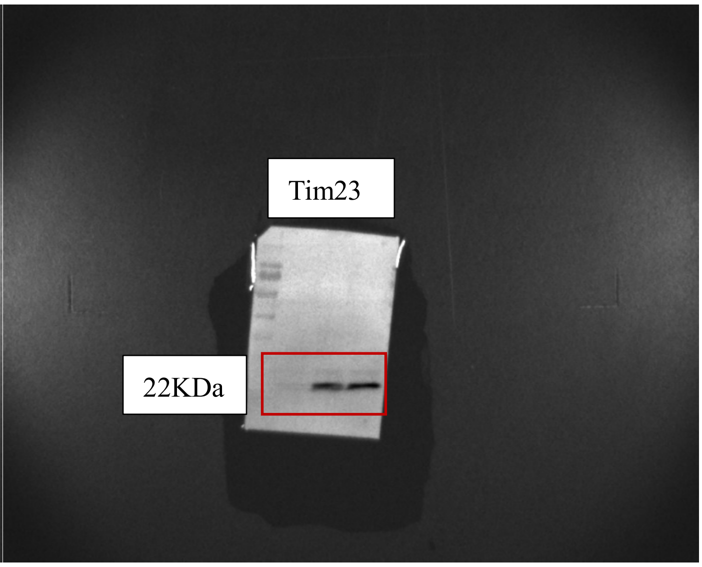
Tim23
22KDa

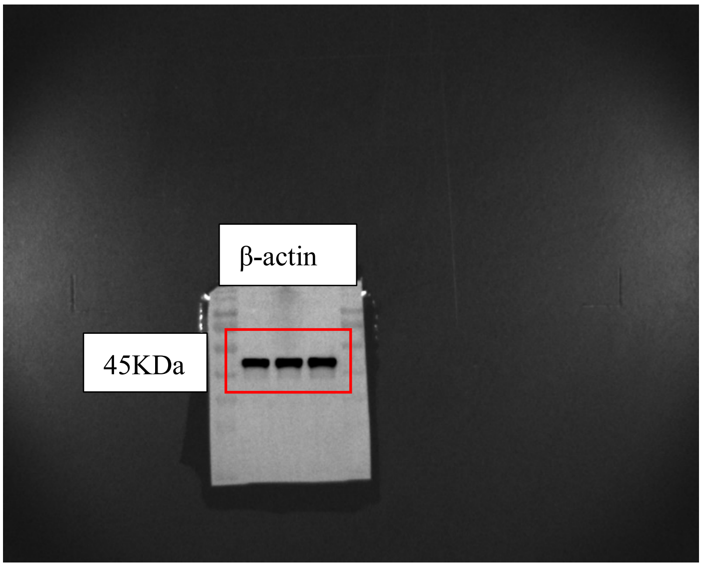

Fig. S5A

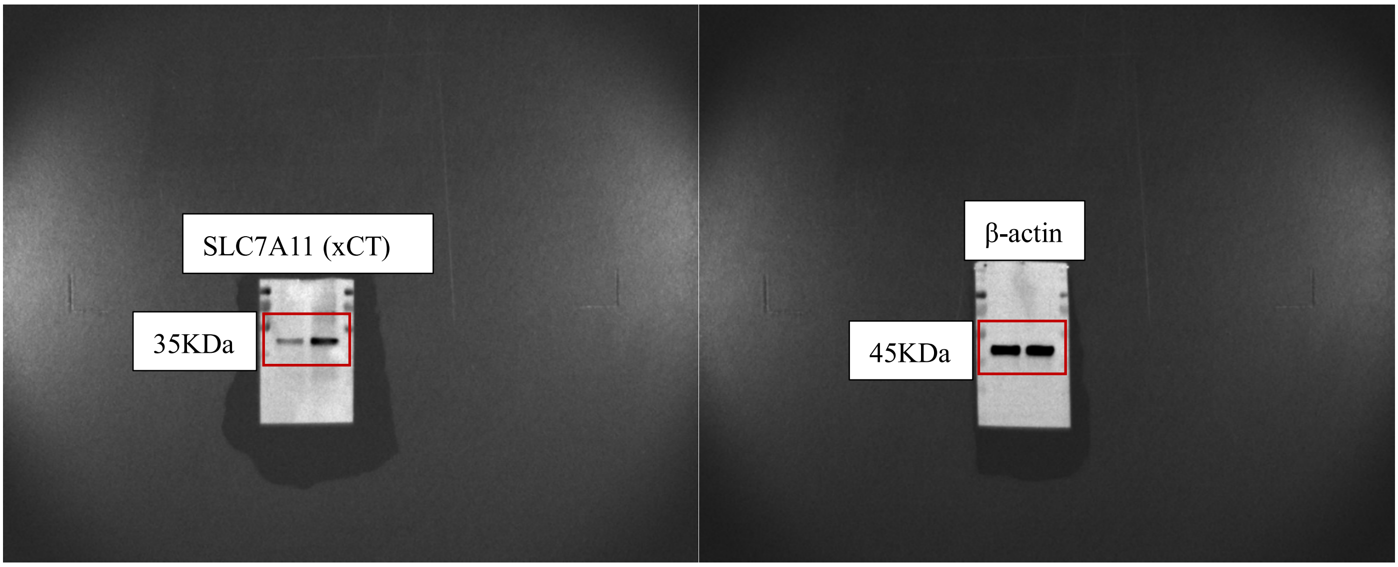

Fig. S5G

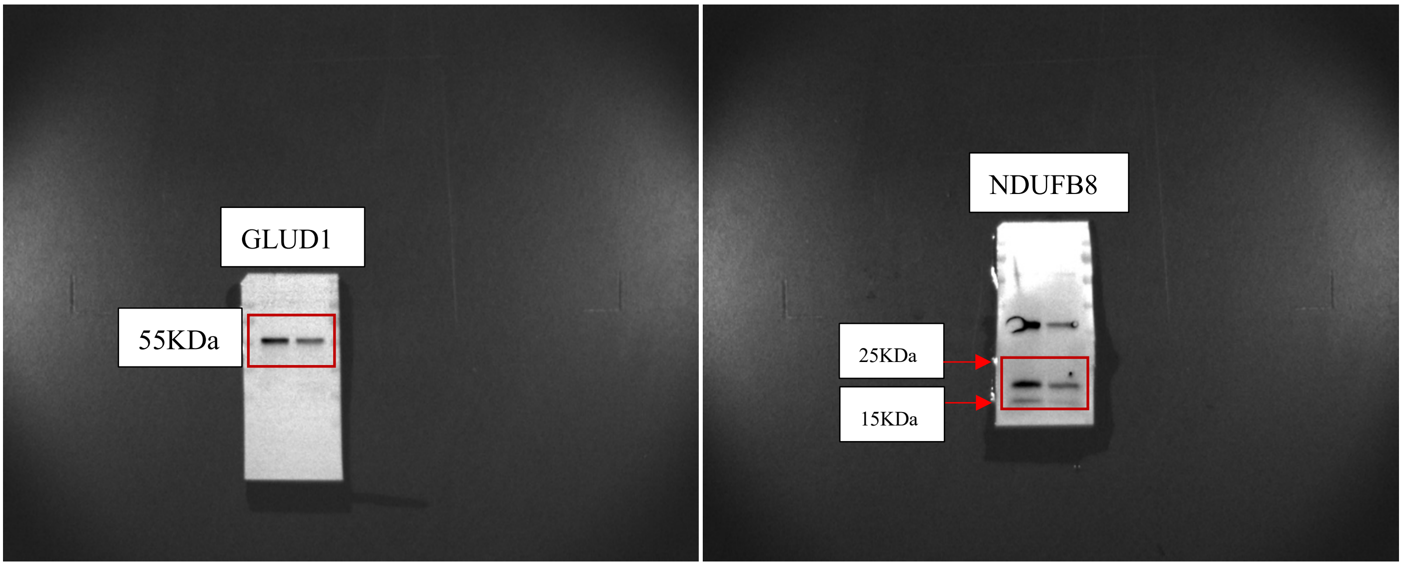

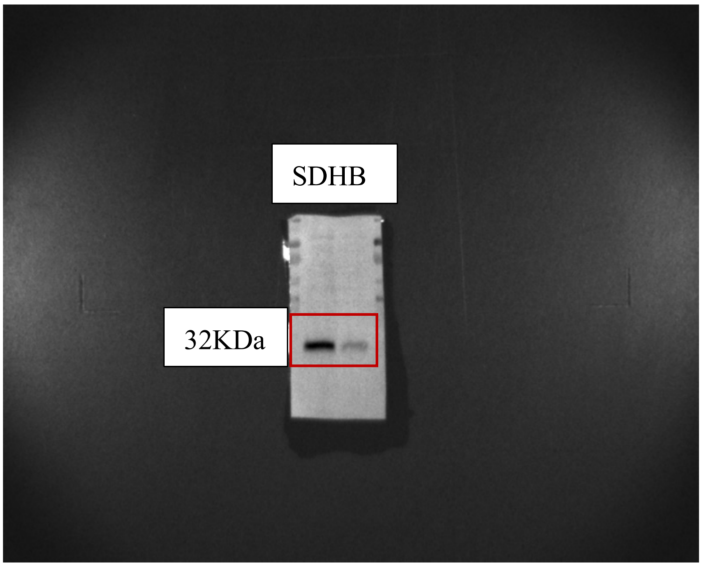
SDHB
32KDa

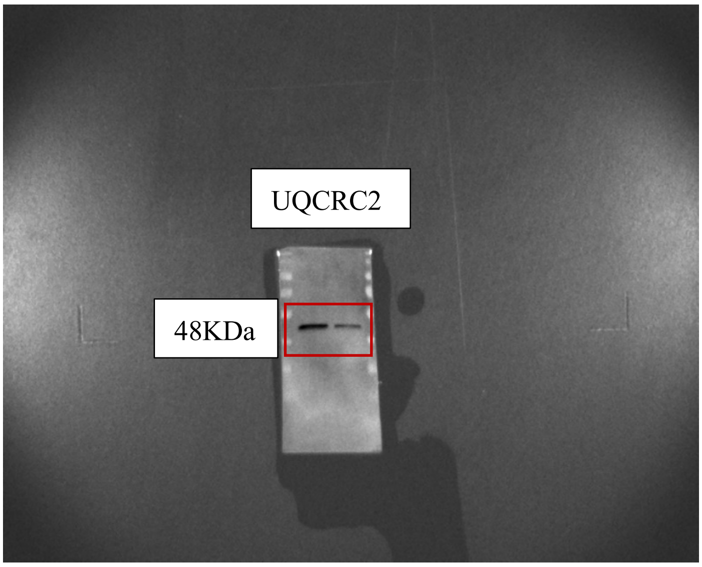
UQCRC2
48KDa

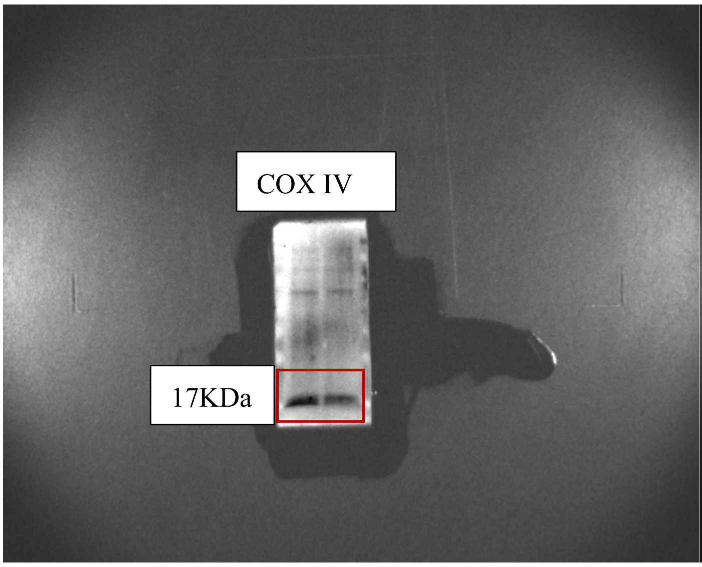
COX IV
17KDa

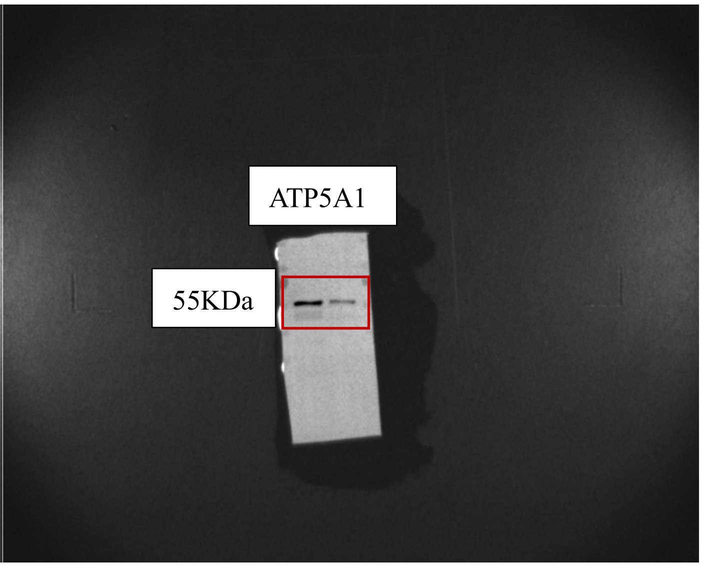
ATP5A1
55KDa

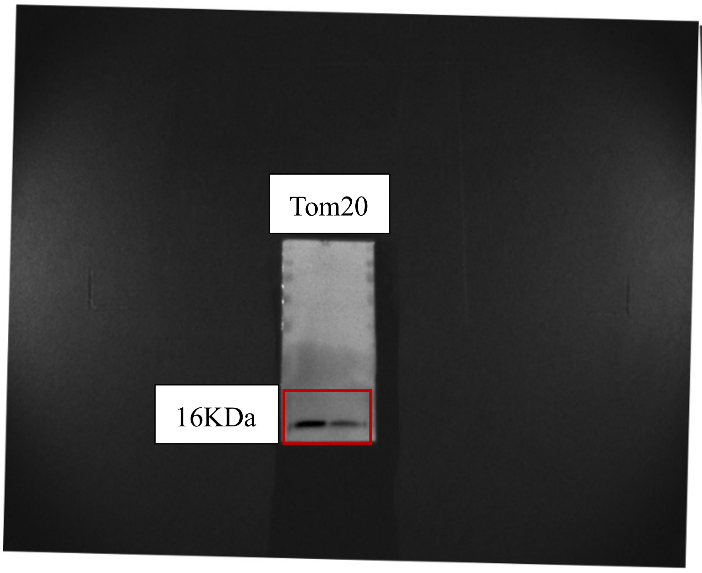
Tom20
16KDa

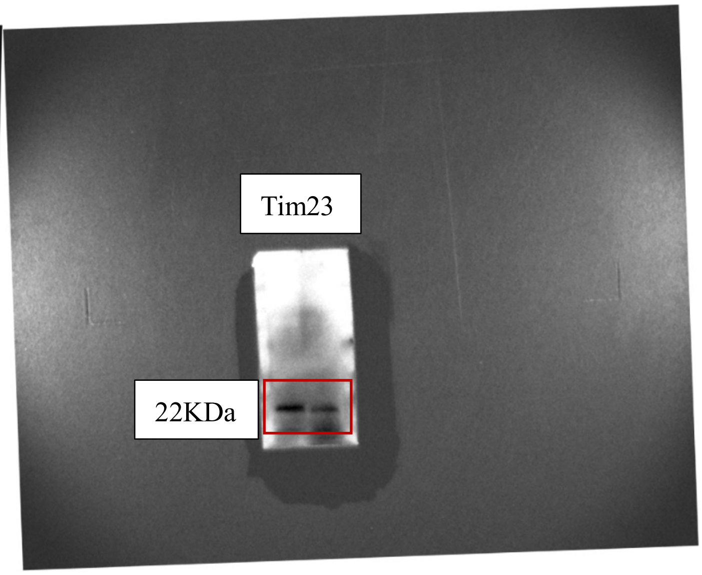
Tim23
22KDa

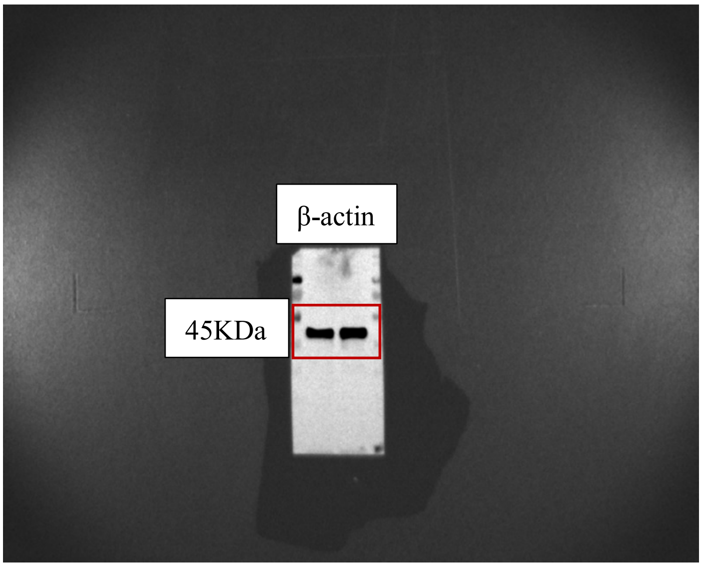
β-actin
45KDa

Fig. S6J

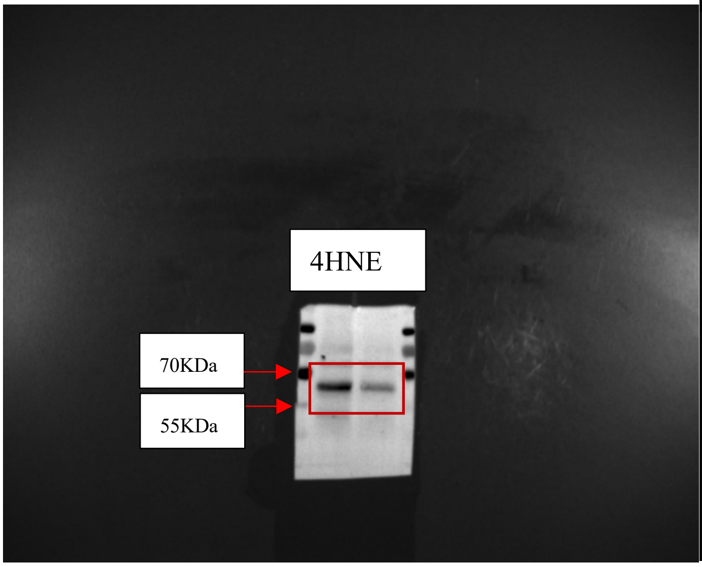
4HNE
70KDa
55KDa

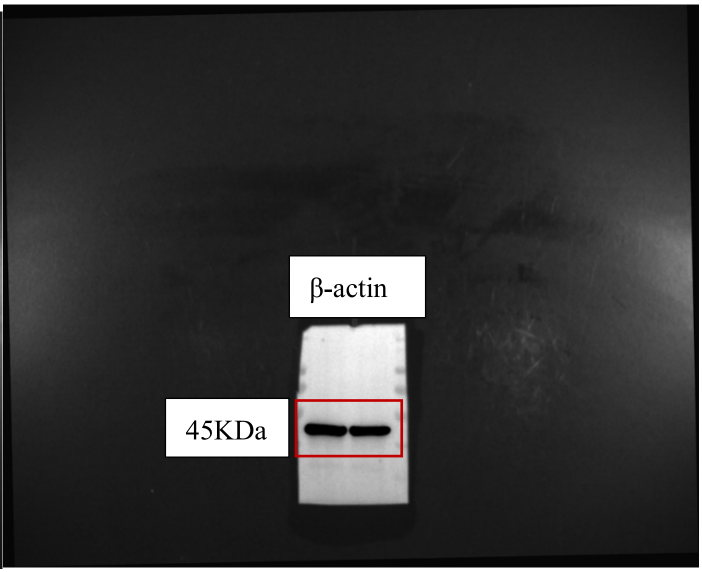
β-actin
45KDa

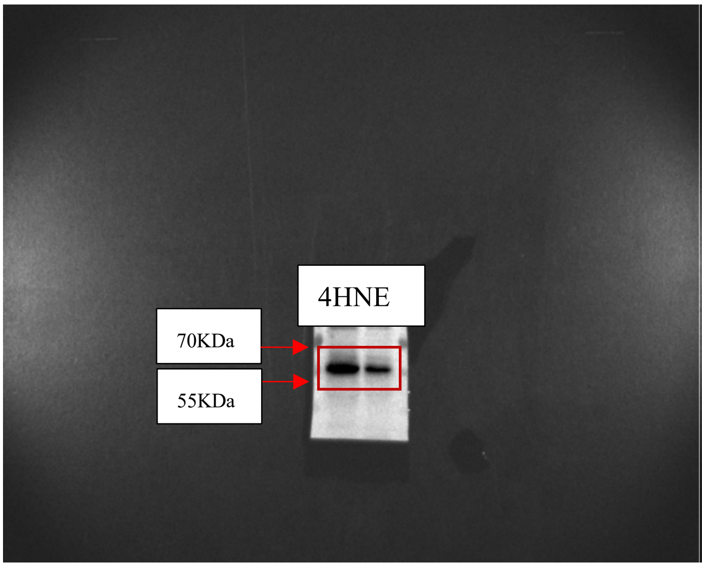
4HNE
70KDa
55KDa

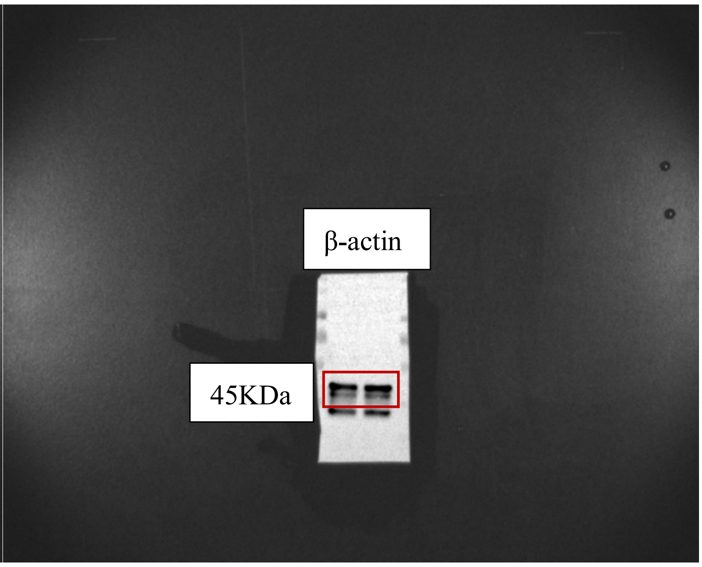
β-actin
45KDa

Fig. S7B

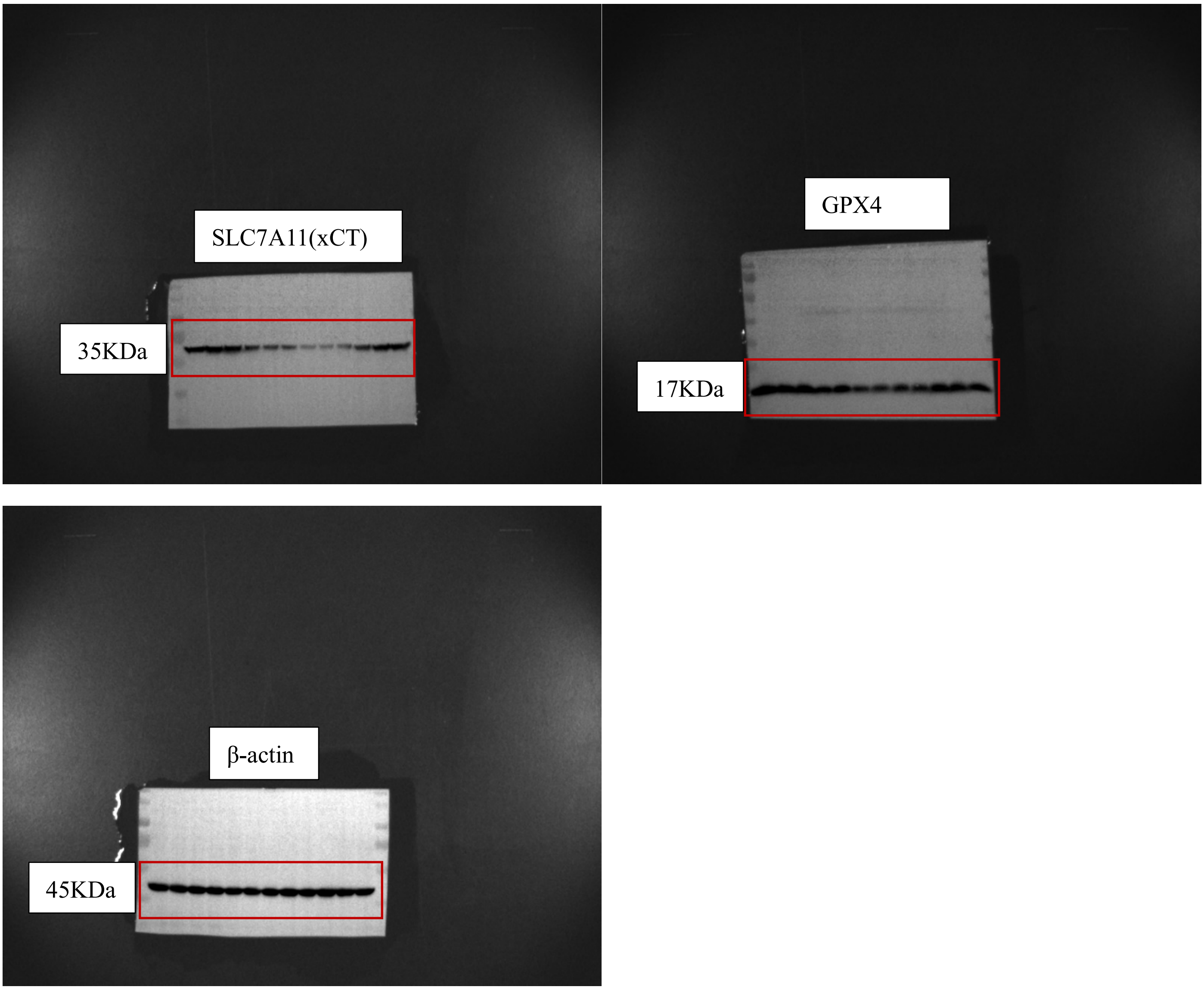

Supplement: Supplementary file 2 — Supplementary Material 2 [file 12964_2024_1581_MOESM2_ESM.pdf]
